# Nanostructuration of Thin Metal Films by Pulsed Laser Irradiations: A Review

**DOI:** 10.3390/nano9081133

**Published:** 2019-08-06

**Authors:** Francesco Ruffino, Maria Grazia Grimaldi

**Affiliations:** Dipartimento di Fisica e Astronomia “Ettore Majorana”-Università di Catania and MATIS CNR-IMM, via S. Sofia 64, 95123 Catania, Italy

**Keywords:** thin metal films, nanostructuration, pulsed laser irradiation, nanosecond, picosecond, femtosecond, dewetting, ablation, deformation, metal nanostructures

## Abstract

Metal nanostructures are, nowadays, extensively used in applications such as catalysis, electronics, sensing, optoelectronics and others. These applications require the possibility to design and fabricate metal nanostructures directly on functional substrates, with specifically controlled shapes, sizes, structures and reduced costs. A promising route towards the controlled fabrication of surface-supported metal nanostructures is the processing of substrate-deposited thin metal films by fast and ultrafast pulsed lasers. In fact, the processes occurring for laser-irradiated metal films (melting, ablation, deformation) can be exploited and controlled on the nanoscale to produce metal nanostructures with the desired shape, size, and surface order. The present paper aims to overview the results concerning the use of fast and ultrafast laser-based fabrication methodologies to obtain metal nanostructures on surfaces from the processing of deposited metal films. The paper aims to focus on the correlation between the process parameter, physical parameters and the morphological/structural properties of the obtained nanostructures. We begin with a review of the basic concepts on the laser-metal films interaction to clarify the main laser, metal film, and substrate parameters governing the metal film evolution under the laser irradiation. The review then aims to provide a comprehensive schematization of some notable classes of metal nanostructures which can be fabricated and establishes general frameworks connecting the processes parameters to the characteristics of the nanostructures. To simplify the discussion, the laser types under considerations are classified into three classes on the basis of the range of the pulse duration: nanosecond-, picosecond-, femtosecond-pulsed lasers. These lasers induce different structuring mechanisms for an irradiated metal film. By discussing these mechanisms, the basic formation processes of micro- and nano-structures is illustrated and justified. A short discussion on the notable applications for the produced metal nanostructures is carried out so as to outline the strengths of the laser-based fabrication processes. Finally, the review shows the innovative contributions that can be proposed in this research field by illustrating the challenges and perspectives.

## 1. Introduction

Researchers working in the nanotechnology field aim to exploit the multitude of functional chemical and physical properties of nanostructures assembled in complex nanodevices [1,2,3]. It is, nowadays, fully established that the chemical and physical properties of nanostructures are significantly different from the corresponding bulk materials due to effects arising from the atomic-scale behavior of matter: surface effects, energy quantization, interference effects, single electron effects, etc. [1,2,3]. The successful development of nanotechnology is dependent on the full exploitation of the “nanofabrication” of shape-, size-, structure-designed nanostructures [4,5,6]. These nanostructures can be, then, integrated into the final functional electronic, optical, magnetic, sensing devices. The problems connected to nanofabrication are the basic ones to be solved in order to reach the full development and exploitation of nanodevices. We can generally define nanofabrication as the collection of processes and methodologies, both physical and chemical, to assemble single atoms or molecules in a controlled manner (top-down approach) in a nanostructure with the desired size, shape, and structure or to “sculpt” (bottom-up approach) massive materials to obtain nanostructures or nanostructured materials with controlled sizes and geometries [4,5,6]. However, to be industrially attractive, these processes and methodologies need to be simple, versatile, and cost-effective, with a high throughput.

In this sense, nowadays, the interaction processes of ions, electrons and photons with matter are, commonly, used both in the “bottom-up” and “top-down” approaches for the fabrication of nanostructures [4,5,6]. These interaction processes lead to energy transfer to the irradiated material so as to induce, for example, the controlled self-assembly of the material atoms and molecules in nanostructures. In particular, in this review, we focus the attention on the use of the laser-matter interaction to structure matter at the nanoscale. In fact, recent developments in the area of fast and ultrafast pulsed lasers (on the range of nano-, pico-, femto-second) have drawn new and fascinating perspectives in the field of nanofabrication: a multitude of nanostructures can be, currently, produced by exploiting the interaction of lasers with thin films deposited on functional substrates allowing a fine control of shape, size, structure on the basis of the process parameters [7,8,9,10]. The main advantages of the laser-based nanofabrication approach include the ability to manipulate materials with dimensions from the micrometer range to the nanometer one, minimize thermal damage to the substrate and neighboring regions, non-contact nature, non-planar manipulations and the possibility of combining this technique with other fabrication steps such as surface chemical treatments [7,8,9,10,11]. Moreover, a great advantage over other techniques is given by the versatility: by simply choosing the laser characteristics (energy density, wavelength, duration of the pulse, number of pulses), a wide “range” of nanostructures can be generated. Finally, by exploring the interference phenomena obtained from the simultaneous use of two or more lasers, complex periodic arrangements of nanostructures can be fabricated [7,8,9,10,11].

**Figure 1:**
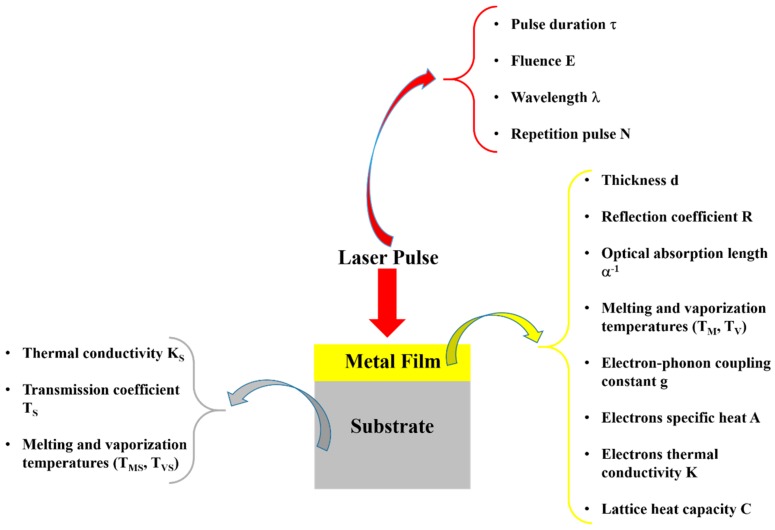
The schematic representation of a metal film deposited on a substrate and processed by a laser pulse to induce the film nanostructuration. In the figure, some critical parameters concerning the laser, the film, and the substrate affecting the nanostructuration process are indicated.

Among the wide-range class of nanostructures, those of metallic nature are particularly interesting and promising [12,13,14,15,16,17,18,19,20,21,22]: in fact, metal nanostructures are the subject of numerous studies for their unique electronic, catalytic, sensing, and optical properties [12,13,14,15]. The production of nanodevices exploiting the characteristics of metal nanostructures is, now, well-established and, in more recent years, this topic has received a new impetus from the plasmonic research field [12,13,14,15,22,23,24,25]. The study of the optical properties of noble metals (especially Au and Ag) represents, to date, a research field of particular interest, as a result of their potential applications in new photonic and sensing devices [12,13,14,15,22,23]. In these applications, the size and shape of the metal nanostructures are crucial in determining the device’s optical response. Complex-morphology metal nanostructures are highly desirable in order to enhance the incident electromagnetic radiation by the so-called hot-spots effect, which is of paramount importance, for example, in Surface-Enhanced Raman Spectroscopy. So, the extraordinary interest in laser-based structuring approaches of thin metal films deposited on substrates arises from the possibility to produce large arrays of tunable surface-supported metal nanostructures. In this case, the size, shape and structure can be widely tunable by the properties of the nano-second, pico-second, and femto-second pulsed lasers used to process the starting metal films by the optical and thermal properties of the metal film and by the optical and thermal properties of the film supporting substrate; see Figure 1.

Depending on the nature of the properties of the metal film, of the substrate, and of the type laser, metal nanoparticles [26,27,28,29,30,31,32,33,34,35,36,37,38,39,40,41,42,43,44,45,46,47,48,49,50,51,52,53], metal microbumps [54], spatially ordered metal nanostructures such as spikes and ripples (laser-induced periodic surface structures) [55,56], metal nanobumps and nanojets [57,58,59,60,61,62,63,64,65,66,67] can be produced; the study of which has continued until very recent times [68,69,70,71,72,73,74,75,76,77,78,79,80,81,82,83].

From a general point of view, a first classification on the pulsed laser effect on a metal film can be made on the basis of the pulse duration: typically, in a metal film subjected to irradiation with nanosecond laser pulses, the dominant dynamics is the material melting; irradiation with femtosecond laser pulses causes more complicated dynamics, including film ablation and deformation [7,8,9,10].

**Figure 2:**
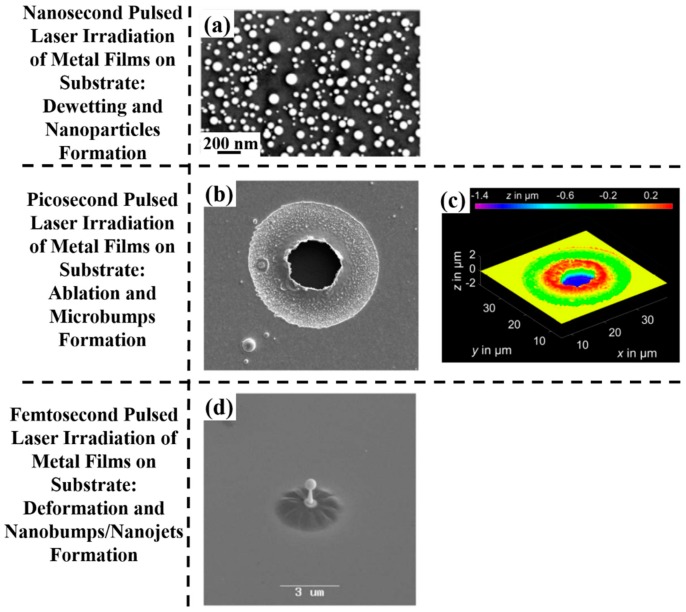
The schematic representation, in terms of structuration, of the effect of nano-, pico-, femto-second pulsed laser irradiations on thin metal films deposited on a substrate: (**a**) a 5 nm-thick Au film deposited on SiO_2_ and processed by a single pulse of a 12 ns-pulsed laser with a wavelength of 532 nm and a fluence of 750 mJ/cm^2^ (scanning electron microscopy image). Reproduced with permission from [45]. Copyright Elsevier, 2012; (**b**,**c**) a 1 μm-thick Au film deposited on glass and processed by a single pulse of a 10 ps-pulsed laser with a wavelength of 1030 nm and energy of 53 μJ ((**a**) an optical microscopy in reflection mode and (**b**) a confocal microscopy). Reproduced with permission from [54]. Copyright Elsevier, 2016; (**d**) a 60 nm-thick Au film deposited on quartz glass and processed by a single pulse of a 30-fs pulsed laser with a wavelength of 800 nm and energy of 78 nJ (scanning electron microscopy image). Reproduced with permission from [63]. Copyright Springer, 2009.

So, for example, arrays of spherical metal nanoparticles on the surface can be easily prepared by exploiting the molten-phase dewetting process of the deposited metal film when irradiated by nanosecond pulsed laser, while complex-morphology metal nanostructures can be obtained by using a pico-second or femto-second pulsed laser, see the schematic examples in Figure 2. Obviously, the optical, thermal, and elastic properties of the metal film also play a key role in the laser-induced structuring process; and, also, the role of the substrate in the formation of the metal nanostructures is significant due to its effect on the propagation of the laser-generated heat (for example, in the case of an oxide substrate, by having a low thermal conductivity, a relatively small amount of laser-generated heat is dispersed through the substrate. As a result, the heat is almost completely confined within the metal film. A substrate having a higher conductivity allows a more efficient heat dispersion through the substrate itself).

The recent literature has mainly focused on the study of the effects, in terms of nano- and micro-structuration, as a result of irradiation with nano-second, pico-second, femto-second pulsed lasers on metal films (Au, Ag, Ni, Mo, Co, Cr, Fe, Ti, Zn, Al, Pt, Cu) deposited on oxide (SiO_2_, Al_2_O_3_, borosilicate glasses, quartz), semiconductor (Si, GaN), transparent and conductive (ITO, FTO) substrates. In this sense, Table 1 summarizes the recent literature concerning the type of used laser (nano-second, pico-second, femto-second) on various metal films deposited on specific substrates with the indication of the typology of the obtained nanostructures. This study shows the growing importance of fast and ultrafast pulsed lasers for nanostructuring metal films on surfaces in view of functional applications. Due to the extension, complexity, and importance of this research field, in the present review, we attempt, by starting from the discussion of specific cases, to focus our attention on the basic microscopic mechanisms and processes and on the general physical concepts suitable for the establishment of the properties of the materials. Starting from the discussion of the characteristics of the laser-metals interaction, the review aims to comprehensively schematize the main classes of metal nanostructures which can be fabricated by exploiting the processing of the deposited metal films with fast and ultrafast pulsed lasers.

In each discussed specific example, the main involved thermal, optical, mechanical parameters and processes governing the metal film structuration are elucidated. In this way, the review aims at establishing a general framework connecting the processes parameters to the characteristics (shape, size, etc.) of the produced nanostructures. This could be a step ahead towards the use of the pulsed laser approach for the controlled design and fabrication of metal nanostructures with desired morphological and structural properties in view of their engineering in real devices. In particular, the review is organized as follows:

The first part (Section 2) is devoted to a synthetic discussion of the phenomena, processes and parameters occurring when metals are irradiated by laser pulses. Starting form general concepts and descriptions, the attention is then focused on the fast and ultrafast pulsed laser interaction with metal films. The roles of the laser pulse duration and the optical, electronic, and thermal properties of the films are particularly highlighted so as to establish general working ranges for the film nanostructuration process.

The second part (Section 3, Section 4 and Section 5) focuses on the illustration and discussion of some seminal literature experimental works on the use of nanosecond (Section 3), picosecond (Section 4), femtosecond (Section 5) pulsed lasers for nanostructuring thin metal films on functional substrates. Starting from the discussions of these examples, the general classes of metal nanostructures which can be produced are highlighted. In addition, in each case, the effect of the film thickness of the film, the optical, thermal and mechanical properties of the supporting substrate, and the optical and thermal properties of the laser energy are discussed. So, as a consequence, the methods to control the obtained nanostructures shape and size are established as a function of the process parameters such as the laser pulse duration and energy, metal film thickness, nature of the substrate. A short part (Section 6) illustrates some notable technological applications for the laser-produced metal nanostructures. In particular, it outlines the strengths of the laser-based fabrication process on the basis of such applications. Finally, the concluding part (Section 7) summarizes the main conclusions and discusses the open points, future perspectives and challenges.

## 2. Laser-Metal Films Interaction: General Considerations

The first and fundamental step in assessing the laser-matter interaction as a nanofabrication tool is understanding the effect of the laser beam on the irradiated material in terms of the laser generated heat in the solid. The laser photons which are absorbed by the irradiated solid give place to an energy source inside the material [7,8,9,10]. So, the response of the electrons and lattice dynamics in the material to this energy source need to be considered. The interaction of the photons with matter occurs mainly, through electronic excitations. In the range of energies of photons with wavelengths from IR (infrared) to UV (ultraviolet), only the conduction and valence electrons contribute in the excitation processes. Then, for typical laser wavelengths ranging from the near IR to the near UV, the laser energy is absorbed by the electrons through inter- and intra-band energy transitions [7]. This results in a non-equilibrium electronic distribution. Then, the electrons thermalization occurs through electron-electron and electron-phonon scatterings. In semiconductors and insulators, the laser photons absorption results in electrons transitions from the valence band to the conduction band and the subsequent thermalization process occurs by electron-hole recombination which tends to re-establish the equilibrium condition. Therefore, the thermalization process is dependent on the specific electronic band structure of the material [7,8,9,10]. However, this recombination process typically occurs in the nanosecond time range scale [7]. The situation is completely different for laser irradiated metals. In this case, the electrons thermalization process is faster (in the femtosecond time range scale) since it is due to the intraband scattering events since the laser photons are absorbed, mainly, by the free electrons of the metal. A first rough approach to describe the metal heating under laser irradiation can be drawn on the basis of the Drude model [7]. Within this model, the electron scattering mean time is connected to the free electrons gas conductivity by σ = ne^2^τ_D_/m, with σ being the conductivity, n being the electron density, m being the electron mass, and τ_D_ being the electron scattering mean time, typically a few femtoseconds [7,8,9,10,11]. More rigorous treatments, however, need to consider the specific characteristics of the non-equilibrium electronic distribution [7]. In these approaches, for example, the lifetime τ_ee_ of the excited electrons, due to electron-electron scattering events, is taken into account and connected to the Fermi energy E_F_ (within a Fermi liquid treatment [7]) by τ_ee_ = τ_0_(E_F_/E-E_F_)^2^ with E-E_F_ being the excited electron energy referred to as the Fermi energy and τ_0_ being a characteristic time in the order of few femtoseconds. Typical values of the laser wavelength in the UV range establish τ_ee_ in the 1–10 fs range. Therefore, the thermalization process occurring by electron-electron interaction is very fast and can be detected by only using laser pulses with a duration comparable to τ_ee_. For longer laser pulses, the electron-electron thermalization process starts and ends within the pulse duration. On the other hand, a second typology of the thermalization process can occur: electron-lattice (phonon) scattering. The thermal relaxation of electrons in metals through the electron-phonon interaction was studied by Allen [7,84]: he developed a model which considers the rate of change of electron and phonon distributions the laser irradiation due to the scattering events, which connects the time evolution of the electronic system temperature T_e_ to the lattice temperature T_L_ by the rate equation [7,84] ∂T_e_/∂t = (T_L_ − T_e_)/τ_ep_. In this equation, the term τ_ep_ represents a characteristic electron-phonon coupling time depending on T_e_, on a coupling constant (without physical units) characteristic of the material, and on the material Debye frequency [7,84]. Considering reasonable values for T_e_ (~1000 K), for the coupling constant (~0.5), and for the Debye frequency (~10 meV), then typical values for τ_ep_ are in the 0.1–1 ps range: the laser generated energy in the electronic system is transferred to the phonon system more rapidly than the characteristic time for the electron-electron energy transfer (since τ_ep_ ≈ 100–1 000 τ_ee_). The consequence is that in metals under pulsed laser irradiations, the thermal dynamic evolutions of the electron and phonon systems occur within very different time scales so that these dynamics can be described separately by two different (but coupled) heat transport equations (jointly named the two-temperatures model) [7]: C_e_(∂T_e_/∂t) = ∇(k_e_∇T_e_) − H(T_e_,T_L_) − S(t) for the electrons system and C_L_(∂T_L_/∂t) = H(T_e_,T_L_) for the phonons system. In these equations, Ce and CL are, respectively, the electronic and lattice specific heats, S(t) is the laser power absorbed by the material per unit volume, H(T_e_,T_L_) represents the rate of energy transfer from the electronic system to the lattice, and ∇(k_e_∇T_e_) represents the diffusion term related to the electron energy transfer. A simplified expression for the rate of energy transfer from the electronic system to the lattice is often used [7,85]: H(T_e_,T_L_) = G_ep_(T_L_ − T_e_) with G_ep_ = C_e_/τ_ep_ called the electron-phonon coupling constant. In metals like Cr, Mo, W, and Fe, the electron-phonon relaxation is rapidly giving place to the fast energy transfer from electrons to phonons and, typically, material removal (ablation). In noble metals like Au and Ag, the electron–phonon relaxation is much slower. For example, G_ep_ ~ 42 × 10^16^ W m^−3^ K^−1^ for Cr and G_ep_ ~ 2.3 × 10^16^ W m^−3^ K^−1^ for Au [47,60,86]. In noble metals, therefore, due to the slower energy transfer from the electron sub-system, the lattice can melt and the molten phase can exist for a long time. In Au, for example, the laser-generated energy is transferred to the lattice within 15 ps and the equilibrium between electrons and phonons takes place within a time limit of up to 50 ps [47,87].

**Figure 3:**
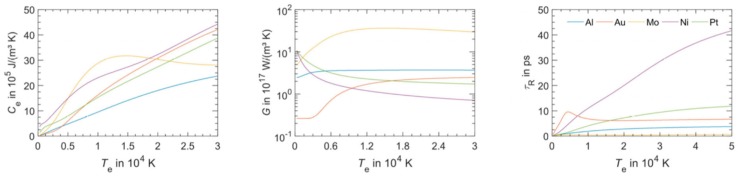
The heat capacity (per unit volume) C_e_, electron-phonon coupling constant G (=G_ep_), the relaxation time to reach a thermal equilibrium between the electron and phonon systems (τ_ep_ = τ_R_) for some selected metals (Al, Au, Mo, Ni, Pt) versus the electronic temperature T_e_. Reproduced with permission from [54]. Copyright Elsevier, 2016

However, in the solution of the two-temperature model equations, the temperature dependence of C_e_ and G_ep_ should be considered. As an example, Figure 3 reports that according to Olbrich et al. [54], the heat capacity (per material unit volume) of electrons (C_e_), the electron-phonon coupling constant (G = G_ep_), and the relaxation time to reach a thermal equilibrium between the electron and phonon systems (τ_R_) for some selected metals (Al, Au, Mo, Ni, Pt) versus the electronic temperature T_e_. On the other hand, considering a laser pulse of duration τ_pulse_, the two-temperatures model equations are useful if τ_p_ is comparable to the lifetime of excited electrons τ_ee_ and to the electron-phonon coupling time τ_ep_ (i.e., for the femtosecond or picosecond laser pulse). In fact, instead, if τ_pulse_ > > τ_ee_, τ_ep_ (as in the case of nanosecond pulsed laser irradiations) then electrons and phonons thermalize within the pulse duration so that T_e_ = T_L_ and their dynamics coincide. To illustrate the difference between these situations qualitatively, the case of Cu can be considered [7] (for which C_e_ = γT_e_ with γ = 10^−4^ J/cm^3^K^2^, C_L_ = 3.4 J/cm^3^K, k_e_ = 4 W/cmK, τ_ep_ ~ 0.3 ps): considering a laser pulse on Cu with τ_pulse_ = 50 fs, then the electrons system and the phonons system are completely independent. Under the laser pulse, the electronic temperature suddenly rises and the nit decreases to the original value within a few τ_ep_. The lattice temperature, instead, is not influenced by the laser irradiation so that it is constant (to the original value before the laser pulse) during all the evolutions of the electron’s temperature. Considering, now, a laser pulse on Cu with τ_pulse_ = 5 ps, then the electronic temperature rises and its time-profile is very similar to the time evolution of the laser pulse (typically gaussian) since τ_ee_ < τ_pulse_. In addition, since the laser-generated energy is transferred from the electrons system to the phonons systems within the pulse duration, the lattice temperature T_L_ increases, reaching a maximum temperature much lower than the maximum temperature reached by the electrons.

**Figure 4:**
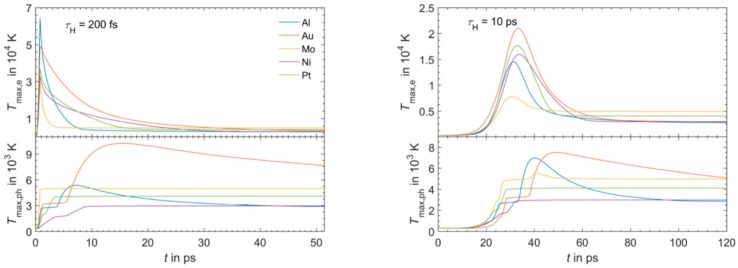
The results of the simulations for the maximum temperature of the electronic system (T_max,e_) and for the maximum temperature of the phonons system (T_max, ph_) as a function of time for Al, Au, Mo, Ni, Pt irradiated by a laser pulse of duration τ_H_ = 200 fs (left) or τ_H_ = 10 ps (right), laser energy of 1 μJ, laser wavelength of 1028 nm. Reproduced with permission from [54]. Copyright Elsevier, 2016.

Finally, considering a laser pulse with τ_pulse_ = 500 ps, then, the electronic and phonons dynamics are practically equal: T_e_ and T_L_ show a very similar time-dependent behaviour so that the lattice is heated in the same way as the electronic system and the two systems are in equilibrium condition. Similar considerations can be drawn for other metals; see Figure 4.

Figure 4 reports the results of calculations performed by Olbrich et al. [54] for the time evolution of the maximum electronic temperature T_max,e_ and of the maximum phonons temperature T_max, ph_ in Al, Au, Mo, Ni, Pt under a pulsed laser irradiation with a pulse duration of τ_H_ = 200 fs (left) or τ_H_ = 10 ps (right), laser energy of 1 μJ, laser wavelength of 1028 nm (and considering, for simplicity, zero reflectance for all the metals, only energy diffusion and no vaporization). From these plots, we can observe, for example, that for all the investigated materials, T_max,e_ is higher for τ_H_ = 200 fs than for τ_H_ = 10 ps since the laser-generated energy is completely transferred from the electrons to the phonons during the laser irradiation at τ_H_ = 10 ps. In addition, we can observe that the highest T_max,e_, for τ_H_ = 200 fs, is reached in Al since it has the lower heat capacity (see Figure 3). On the other hand, the irradiation for τ_H_ = 10 ps causes the highest T_max,e_ in Au since Au possess the higher electron-phonon relaxation time (see Figure 3). This fact justifies the retarded occurrence for Au (with respect to the other investigated metals) of T_max, ph_, after about 50 ps from the laser pulse. Furthermore, for Au, the maximum value for T_max, ph_ is reached later than the maximum value for T_max,e_ due to the high value of the electron-phonon relaxation time τ_R_.

Now, generally speaking, the specific phenomena taking place (and determining the subsequent material modifications) in a metal when it is irradiated by a laser pulse depend, obviously, on the amount of deposited laser energy and its spatial and temporal distributions [7,88]. As previously stated, lasers with a wavelength in the near IR-near UV region interact only with the free electrons of a metal [7] gaining energy from the electric field and being accelerated. These oscillating electrons also re-emit energy, determining the typical high reflectivity of metals. Furthermore, as stated, the description of the interaction of laser with matter can be simplified if the pulse duration is long compared to the typical scattering times (picoseconds): in this case, the classical Drude theory [7] can be used for the description. Using this model in particular, the optical properties of the metal can be described by the dielectric index ε = ε_1_ + iε_2_ with ε_1_ = n^2^ − κ^2^ = 1 − [(ω_p_^2^τ_D_^2^)/1 + ω^2^τ_D_^2^] and ε_2_ = 2nκ = (ω_p_^2^τ_D_)/[ω(1 + ω_p_^2^τ_D_^2^)] being n and κ, respectively, the so-called refractive index and extinction coefficients, ω = 2πc/λ (λ the laser wavelength), τ_D_ being the mean time between two electronic collisions, and ω_p_ = √Ne^2^/mε_0_ (called the plasma frequency) with N the free electron density, e the electron charge, m the electron mass, ε_0_ the vacuum dielectric constant. Within this approximation, the metal reflectivity R and the absorption coefficient α are related to n and κ by R = [(n−1)^2^ + κ^2^]/[(n + 1)^2^ + κ^2^] and α = 4πκ/λ and, furthermore, the plasma frequency is related to the electrical conductivity σ_el_ of the metal by σ_el_ = ω_p_^2^τ_D_ε_0_. In particular, in the IR spectral range (optical wavelengths), the previous equation can be further simplified due to ω < < 1/τ_D_ so that R ≈ 1–2 √2ωε_0_/σ_el_ and α≈√2ωσ_el_/c^2^ε_0_ resulting typically in R ≈ 90%–99% and α^−1^ ≈ 10 nm for ω < ω_p_.

The laser energy absorbed by the metal is then spatially distributed due to heat conduction: in this regard, the thermal properties of the metal and of the supporting substrate play a significant role on the metal modifications. For a laser pulse of duration up to tens of ns, the thermal diffusion length is [7] z_th_ = √τ_pulse_k_e_/C_L_ < 1 μm, k_e_ being the metal heat conductivity and C_L_ being the metal specific heat. In order to describe the heat diffusion in the metal by a simplified one-dimensional heat diffusion equation, the laser spot size must be larger than z_th_ and, in this case, the simplified equation takes the form [7] C_L_(T)[∂T(z,t)/∂t] = ∂/∂z[k_e_(T)(∂T(z,t)/∂z)] + S(z,t) with T(z,t) being the temperature and the depth z and time t; S(z,t) = I_abs_(z,t) + ΔU(z,t) is the energy absorbed by the material from the laser (I_abs_) summed to the possible material internal heat sinks (ΔU) due to phase transformations. In particular, I_abs_ can be related to the metal reflectivity R, to the absorption coefficient α, and to the laser irradiance I(t) (laser energy per unit area and unit time) [7] by I_abs_ = I(t)α(1 − R)exp(−αz).

It is interesting to illustrate some simple consequences of the material heating and cooling stages [89], assuming, as a first rough approximation, the temperature-independent values for the material optical and thermal properties and a laser pulse with a rectangular temporal profile of duration τ_p_. The heating process involves two characteristic lengths, the absorption length α^−1^ and the heat diffusion length l_th_ = √2Dτ_p_, D being the heat diffusivity. In the case of α^−1^ < l_th_, the heat source is restricted to the material surface and the material temperature increase is [89] ΔT ≈ [I_0_(1-R)/k_e_](Dτ_p_/2)^1/2^ with I_0_ = I(t) being the laser irradiance. So, in this case, the energy density required to increase the surface temperature, for example, to the melting temperature, is proportional to the square root of the pulse duration and is independent on the absorption coefficient. The heating and cooling rates are both characterized by τ_p_. The heating rate, in particular, is given by (ΔT/τ_p_) = [I_0_(1 − R)]/[ρC_L_(2Dτ_p_)^1/2^] with ρ being the material density. The heating rate is then inversely proportional to (τ_p_)^1/2^. On the other hand, in the case α^−1^ > l_th_, the temperature increase at depth z is [89] ΔT≈[I_0_(1 − R)ατ_p_exp(−αz)]/ρC_L_ which furnishes a heating rate ΔT/τ_p_, which is independent of the duration of the laser pulse and which exponentially decreases with the depth in the material.

To complete this brief starting overview, we can consider, in addition, that if during the heating stage the material reaches temperatures higher than the melting temperature, then a significant material evaporation can occur so that an appropriate equation for the evaporation flux should be added in the calculations [7]. Furthermore, at laser molten metal surfaces, many mechanisms contribute to the material transport phenomena being the most important one connected to the temperature dependence of the material surface tension.

In the next sections, the effects of nanoseconds, picosecond, femtosecond pulsed laser irradiations on thin metal films deposited on substrates will be experimentally presented, highlighting, on the basis of the general considerations exposed in the present section and on the basis of further specific considerations, the microscopic involved processes, the mechanisms and parameters. In this sense, these processes will be analyzed in view of their potential exploitation as nanostructuring tools for metal films towards the controlled fabrication of metal nanostructures on the substrates for various technological applications.

## 3. Nanostructuration of Thin Metal Films by Nanosecond Pulsed Laser Irradiations

Nanosecond pulsed laser irradiations of thin metal films on substrates is, nowadays, usually used to induce a molten-state dewetting process of the metal films resulting in the formation of nanoscale size metal droplets which can be used, for example, as plasmonic systems in several optical, catalytic, and sensing applications [26,27,28,29,30,31,32,33,34,35,36,37,38,39,40,41,42,43,44,45,46,47,48,49,50,51,52,53,68,69,70,71,72]. Several studies focused on the study of the microscopic thermodynamic and kinetic mechanisms involved in the dewetting process so as to reach a strict control on the dewetted nanoparticles, morphology, size, surface density, etc. [26,27,28,29,30,31,32,33,34,35,36,37,38,39,40,41,42,43,44,45,46,47,48,49,50,51,52,53,68,69,70,71,72].

**Figure 5:**
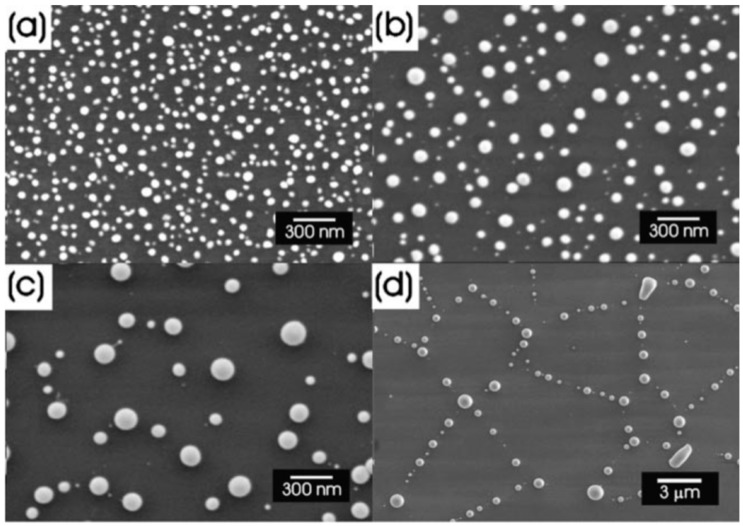
The Scanning Electron Microscopy (SEM) images of Ni films with different thicknesses (**a**) 6.5-nm, (**b**) 8.2 nm, (**c**) 11.5 nm, (**d**) 15 nm) on 320-nm SiO_2_ and irradiated by a 25 ns-pulsed laser at a wavelength of 248 nm with a fluence of 200 mJ/cm^2^ (**a**), 160 mJ/cm^2^ (**b**), 140 mJ/cm^2^ (**c**), 220 mJ/cm^2^ (**d**). Reproduced with permission from [26]. Copyright American Institute of Physics, 2004.

**Figure 6:**
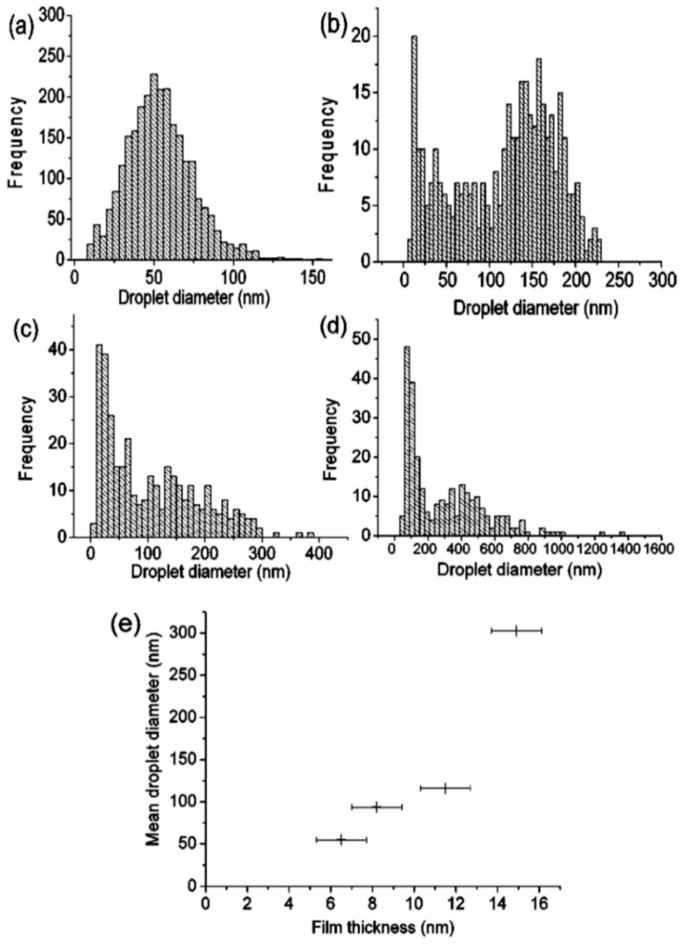
(**a**–**d**) The size distributions corresponding to the Ni nanoparticles showed in Figure 5. (**e**) The plot of the mean diameter of the nanoparticles versus the initial film thickness. Reproduced with permission from [26]. Copyright American Institute of Physics, 2004.

**Figure 7:**
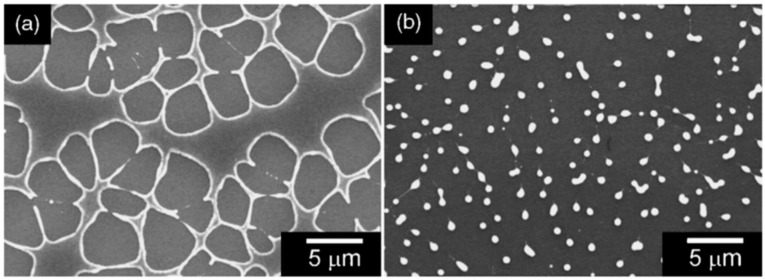
The SEM images of 20-nm-thick Mo films (on 235 nm SiO_2_ thermal grown on Si) treated by laser irradiations (248-nm wavelength, 25-ns pulse duration) at (**a**) a laser fluence slightly below the critical value for complete dewetting (<660 mJ/cm^2^) and (**b**) at a laser fluence slightly above this critical value (>660 mJ/cm^2^). Reproduced with permission from [34]. Copyright Elsevier, 2007.

Henley et al. [26] used nanosecond pulsed laser irradiations for nanostructuring Ni films deposited on SiO_2_/Si substrates (a pulse duration of 25 ns, repetition rate of 10 Hz, fluence in the 100–3 00 mJ/cm^2^ range, wavelength of 248 nm). In particular, they observed that the irradiation processes result in the formation, from the continuous Ni films, of nanoscale sized hemispherical droplets whose mean diameter is controlled by the starting thickness of the Ni film. For these experiments, the authors used, as supporting substrates for the Ni films, some SiO_2_/Si slides with different values of oxide thickness values: 235 and 320 nm of thermal oxide on Si or samples with only the native oxide coating the Si. Figure 5 presents the representative Scanning Electron Microscopy (SEM) micrographs of Ni nanoparticles obtained by the laser irradiations of the Ni films with different initial thicknesses, grown on 320 nm-thick SiO_2_ on Si substrates. It is clear that after the laser irradiations, the Ni film breaks up into nanoscale-size droplets with circular sections. In this regard, the minimum fluence required to induce the film rupture with the consequent formation of the nanoparticles is a function of the initial film thickness and also a function of the thickness of the oxide layer. At fluences lower than this minimum fluence value, the films were observed to perforate, but the break up into discrete droplets was incomplete. So, in this case, significant ablations of the Ni above 280 mJ/cm^2^ took place while, at intermediate fluences, the Ni droplets’ size was unaffected by the laser fluence and by the oxide thickness. Interestingly, the fluence required to form nanoparticles from the films grown on the thinner (235 nm-thick) SiO_2_ substrates was higher than for the corresponding Ni film on the 320 nm-thick SiO_2_ layers. Furthermore, for Ni films on the Si substrates with only the native oxide layer, no nanostructuring was observed, indicating that the threshold fluence for dewetting was higher than the ablation threshold of the film: this behaviour is, clearly, due to the higher thermal conductivity of Si (150 Wm^−1^ K^−1^) than that of SiO_2_ (1.34 Wm^−1^ K^−1^). Clearly, the underlying SiO_2_ layer, with its low thermal conductivity, better confines the laser generated-heat in the metal film than the Si substrate. The lower thermal conductivity of the substrate allows, then, for the reaching of higher temperatures in the metal films, often higher than the metal films’ melting temperature, resulting in the molten-state dewetting of the films. Figure 6a–d presents some representative size distributions of the Ni nanoparticles: Figure 6a shows a mono-modal distribution, Figure 6b–d show bi-modal distributions. The reason for this difference is as follows: at fluences lower than the critical one for the complete film dewetting, only partial film perforation and contraction occurs. These arise by molten film retraction and the retraction of the molten film continues away from the center of the formed hole. When the size and density of the holes are high, the holes can coalescence, leaving molten metal filaments. In this stage, the starting continuous film evolved so as to be structured in large particles connected by a web of filaments. At slightly higher fluences, these filaments, which are thermodynamically unstable, can split into smaller droplets. To summarize, Figure 6e presents the correlation of a mean diameter of the Ni nanoparticles to the initial film thickness showing an increase of the mean nanoparticles’ size, increasing the thickness of the deposited film. Thus, the film thickness can be used to control the size distribution of the Ni nanoparticles. Henley et al. extended their studies to several other metals (Au, Ag, Mo, Ti, Zn) deposited on various substrates (SiO_2_, ITO) [27,28,34] in order to determine what materials and laser parameters are required to produce nanoparticles and to draw insights on the structuring mechanisms. Figure 7 reports SEM images of 20-nm-thick Mo films deposited on 235 nm-thick SiO_2_/Si and laser irradiated (248 nm wavelength, 25 ns pulse duration) at (a) a laser fluence slightly below the critical value for complete dewetting (<660 mJ/cm^2^) and (b) at a laser fluence slightly above this critical value (>660 mJ/cm^2^).

**Figure 8:**
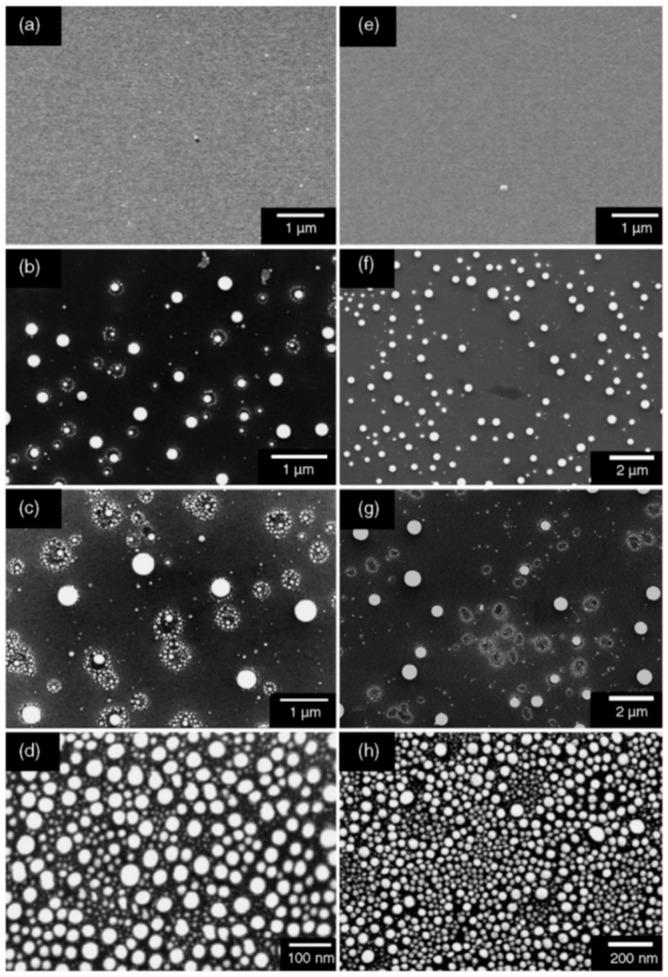
The SEM images of (**a**) 20 nm-thick Au as-deposited on 230 nm SiO_2_/Si, and, then, the 20 nm-thick Au film laser-processed with (**b**) a 125 mJ/cm^2^ fluence, (**c**) 250 mJ/cm^2^ fluence, (**d**) and 430 mJ/cm^2^ fluence. In addition, SEM images of (**e**) 15 nm-thick Ag as-deposited on 230 nm SiO_2_/Si, and, then, the 15 nm-thick Ag film laser-processed with (**f**) 150 mJ/cm^2^ fluence, (**g**) 3000 mJ/cm^2^ fluence, and (**h**) 400 mJ/cm^2^ fluence. Reproduced with permission from [34]. Copyright Elsevier, 2007.

**Figure 9:**
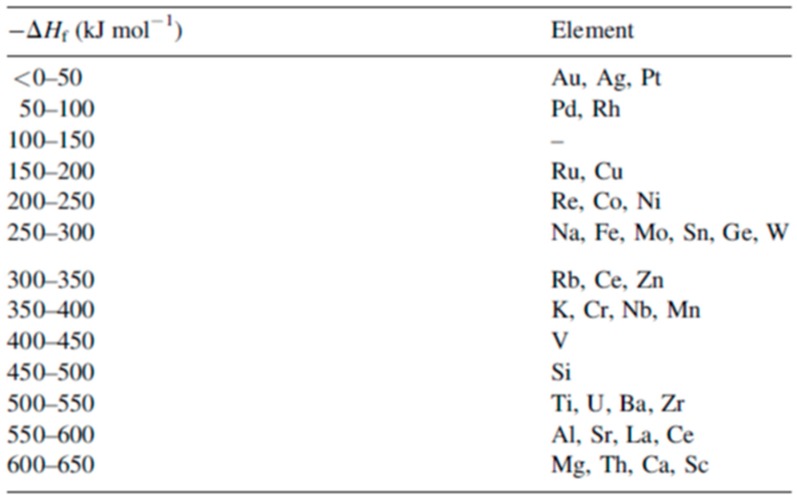
The enthalpy of formation −ΔH_f_ of the oxide per mole of O for some metals. Reproduced with permission from [34]. Copyright Elsevier, 2007.

To complete the nanostructuring process of the film in droplets, a much higher fluence than that for Ni films was required since Mo has a much higher melting point (1455 °C for Ni, 2623 °C for Mo). Just below the rupture threshold, the molten films perforate and film retreating process starts with the formation of the characteristic holes and rims at the hole edges (see Figure 7a). The starting perforations in the molten film occur at thickness inhomogeneities in the film. When the size and density of the perforations is high, holes coalescence occurs so as to give origin to molten metal wires which, being thermodynamically unstable, decay in droplets due to the Rayleigh instability in order to minimize the total surface energy of the system; see Figure 7b [27,34,45]. In addition, Figure 8 shows a series of SEM images for laser-processed (248 nm wavelength, 25 ns pulse duration) Au and Ag films on 235 nm-thick SiO_2_/Si. In particular, the figure presents SEM images of (a) 20 nm-thick Au as-deposited on 230 nm SiO_2_/Si, and, then, the 20 nm-thick Au film laser-processed with (b) 125 mJ/cm^2^ fluence, (c) 250 mJ/cm^2^ fluence, (d) and 430 mJ/cm^2^ fluence. In addition, the figure also reports SEM images of (e) 15 nm-thick Ag as-deposited on 230 nm SiO_2_/Si and the 15 nm-thick Ag film laser-processed with (b) 150 mJ/cm^2^ fluence, (c) 3000 mJ/cm^2^ fluence, (d) and 400 mJ/cm^2^ fluence. In these cases, the film evolution (perforation and break-up into islands) is similar for Au and Ag and, generally similar to that observed for Ni and Mo. However, for Au and Ag, well-above the threshold (see Figure 8c,g) smaller particles are observed around the larger ones: they arise from the nanoparticles boiling due to the relative lower boiling temperature for Au and Ag in comparison to Ni and Mo (2700 °C for Au, 2162 °C for Ag, 2913 °C for Ni, 4639 °C for Mo). In same conditions, different results were obtained for the Ti and Zn films on SiO_2_ after the laser irradiations [27,34]: in these cases, in correspondence of any used laser fluence, no nanoparticles were obtained. This is a strong indication that the interfacial metal-substrate interaction is of paramount importance for the nanoparticles’ production. In this regard, Figure 9 reports a comparison of the enthalpy of formation of the oxides, ΔH_f_, for various metals, including those used by Henley et al. The data for Au, Ag, Ni and Mo (those for which the dewetting process occurs upon laser irradiation) are all in the top half of the table, with ΔH_f_ < 300 kJ/mol, i.e., they are non-wetting metals. On the contrary, Zn and Ti better wet the substrate since ΔH_f_ > 300 kJ/mol. Thus, the non-wetting nature of the metals on the substrate appears as a fundamental condition for the laser-induced dewetting process.

**Figure 10:**
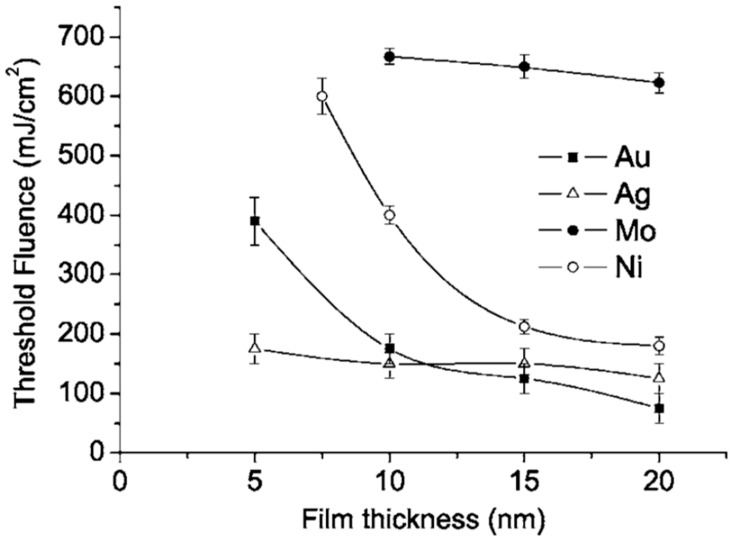
The plot of the experimentally observed melting fluence for Ag, Au, Mo, and Ni thin films as a function of the film thickness. Reproduced with permission from [27]. Copyright American Physical Society, 2005.

To further investigate the nanostructuring mechanisms, Henley et al. measured the laser fluence threshold for melting Ni, Mo, Au and Ag films versus the film thickness; see Figure 10 [27]. It is clear that higher laser fluences were required to dewet thinner Ni films indicating that the heat conduction from the film to the substrate is significant. In addition, as indicated by the SEM images, just below the break-up threshold, the films perforate at thickness inhomogeneities in the film since the fluence required for melting is lower for thicker films. At fluences higher than the threshold for breakup, no change in the Ni droplet size distribution was observed when the film thickness is fixed, while the mean nanoparticles size was found to increase by increasing the initial thickness of the deposited film. As stated, similar results were obtained for Mo, Au and Ag deposited films.

**Figure 11:**
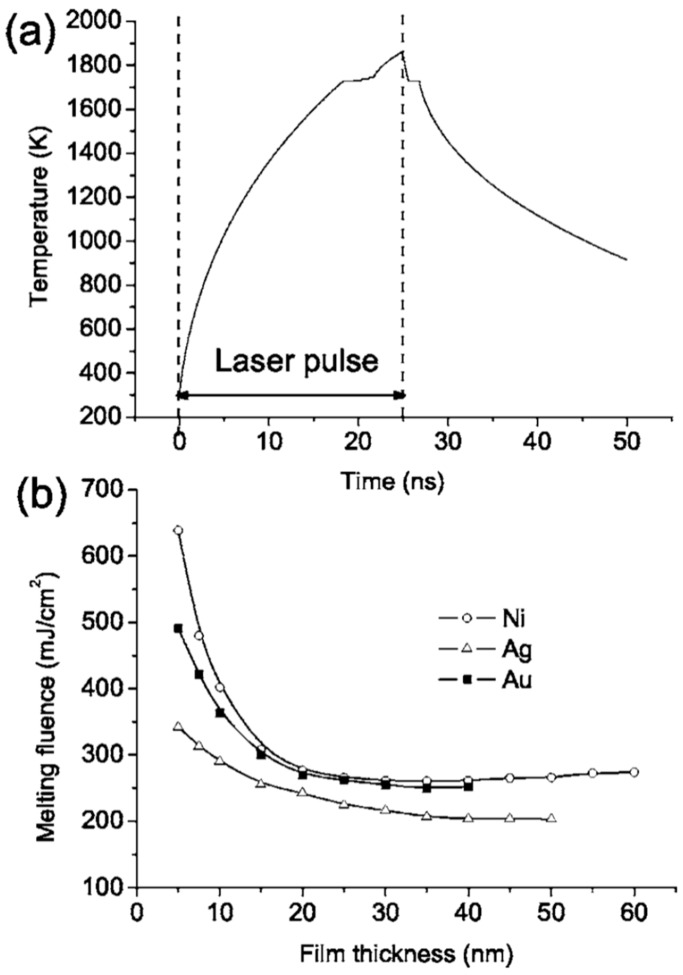
(**a**) The simulated temperature temporal profile for the surface layer of a 20 nm-thick Ni thin film processed by a 25-ns pulsed laser at 330 mJ/cm^2^. (**b**) The plot of the simulated fluence required to melt Ni, Au, and Ag films of different thicknesses. Reproduced with permission from [27]. Copyright American Physical Society, 2005.

However, the authors also observed that, by cross-sectional SEM images, the contact angle of the dewetted nanoparticles increase by decreasing the wetting nature of the metal on the SiO_2_, i.e., by decreasing ΔH_f_ [27]: 120° for Au nanoparticles on SiO_2_, 118° for Ag nanoparticles on SiO_2_, 105° for Ni nanoparticles on SiO_2_, and 78° for Mo nanoparticles on SiO_2_. For Ti and Zn no breakup into nanoparticles was observed even if we have a lower melting temperature and lower thermal conductivity. Thus, for the break-up of the film, the critical parameter is the metal-substrate interaction, as expressed by the wetting/non-wetting nature related to the ΔH_f_ value (even if ΔH_f_ is not the only parameter affecting the wetting/non-wetting behaviour). To analyze the melting of the metal film, Henly et al. [27] considered that with the pulse duration of a few tens of nanoseconds, the temperature change in the film can be described by the one-dimensional heat conduction equation Cρ(∂T/∂t) = I(z,t)α + [(∂/∂z)(k(∂T/∂z))] with C being the metal heat capacity, ρ being the metal density, T being the temperature at depth z and time t, I being the laser power density, α being the metal absorption coefficient, and k being the metal thermal conductivity. In addition, the total heat Q per unit area deposited in a thin film of thickness d, irradiated by a single laser pulse of duration τ_p_ supposed, for sake of simplicity, to have a top-hat temporal profile, can be represented by Q = Iτ_p_(1 − R)[1 − exp(−αd)] with R (the film reflectivity). Thus, by neglecting the heat conduction, the temperature rise in the thin film is ΔT∝Q/d. Considered that when d → 0 then [(1 − exp(−αd))/d] → α and neglecting heat conduction, then ΔT is significant only when α is large and a thicker film require higher fluences to melt. However, heat conduction cannot be neglected: the rate of heat diffusion from the metal film into the substrate is greater as the film thickness decreases since the temperature gradient across the film increases by decreasing the film thickness. Considering heat conduction, thus, when d < (1/α), a part of the substrate, determined by the thermal diffusion depth of the substrate, is heated. As a consequence, the laser fluence needed for the melting of the film increases with decreasing d. On the other hand, when d > (1/α), then, the heat conduction into the substrate is negligible and the lower fluence is required to melt the film. On the basis of these considerations, according to Henley et al. [27], Figure 11a reports a simulation of the temperature time evolution for a 20 nm-thick Ni film on the SiO_2_ irradiated by a 25-ns laser pulse with a fluence of 330 mJ/cm^2^. According to this calculation, the film temperature rapidly increases during the pulse duration and reaches a level higher than the material melting temperature. At the end of the laser pulse, the film rapidly cools within 100 ns. The simulation shows that rapid cooling through heat conduction into the substrate limits the maximum temperature at a given fluence. The results of this type of simulations also allowed us to calculate the fluence required to melt Ni, Au, and Ag films of different thicknesses—see Figure 11b [27]—and these values agree well with the experimental ones (Figure 10).

**Figure 12:**
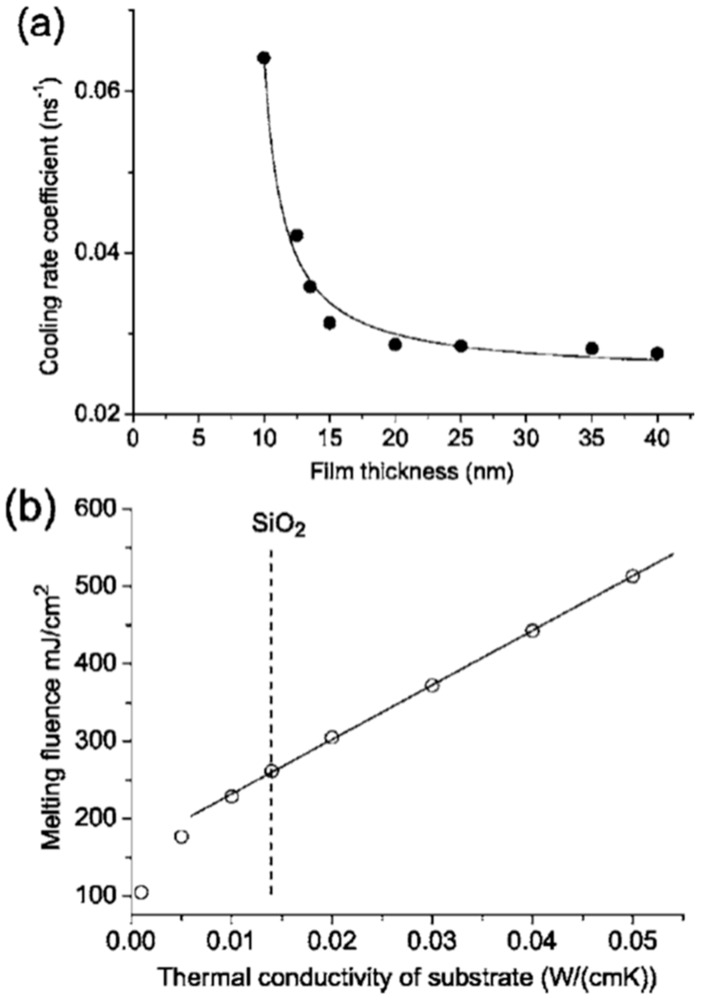
(**a**) The simulated cooling rate coefficient for Ni films of different thicknesses, initially at the melting temperature. (**b**) The plot of the calculated melting fluence for a 30 nm-thick Ni thin film as a function of the room temperature thermal conductivity of the substrate. The plots refer to a 25 ns pulse and to a laser fluence of 330 mJ/cm^2^. Reproduced with permission from [27]. Copyright American Physical Society, 2005.

As stated, the thermal conductivity of the substrate and the initial film thickness were both critical parameters in determining the threshold fluence for the film melting, dewetting and nanostructuring. Specifically, in this regard, Henley et al. [27] also simulated the effect of the thermal conductivity of the substrate on the melting fluence. Figure 12a presents the calculated cooling rate coefficient versus the film thickness for the cooling of the Ni layer on SiO_2–_, which is initially at the melting temperature; it is interesting to note the increase of the cooling rate by decreasing the film thickness. The effect of the substrate thermal conductivity is calculated in Figure 12b: the plot shows the calculated melting fluence for a 30-nm-thick Ni film versus the room temperature thermal conductivity of the substrate. The result shows, as expected, a linear increase of the melting fluence by increasing the substrate thermal conductivity.

When the fluence is higher than the threshold for melting, the film dewetting process can occur if the metal film does not wet the substrate. It should be considered, also, that real films present a natural surface roughness which is often quantified by the RMS (Root Mean Square) parameter. Generally, for thick films, the surface roughness is expected to be lower than that for the thinner films. At thickness inhomogeneities in the film, the fluence required for melting will change and for this reason, the film RMS is an important parameter to be considered in the dewetting process since the natural surface roughness results in a local change in the threshold fluence for melting and determining the local hot spots at thicker regions. The melting and dewetting starts from these regions. The dewetting process starts with molten film perforation and the molten film around these perforations draws away from the perforations originating in the holes. According to Favazza et al. [30,32,33,35,36], the molten metal retraction velocity can be estimated by v = √2|S|/ρd with ρ being the metal liquid density, d being the film thickness and S = γ_S_ − γ_F_ + γ_F/S_ being the spreading coefficient, γ_S_ being the substrate surface energy, γ_F_ being the film surface energy, and γ_F/S_ being the film-substrate interface energy. When the size and density of the holes is high, then, the retreating molten film between two holes can coalesce into liquid nanowires which are thermodynamically unstable and they decay in nanometer-scale droplets by the Rayleigh instability process. This last stage was, in particular, studied by Ruffino et al. [45]. In this work, the authors deposited a 5 nm-thick Au film on the SiO_2_/Si substrate (with SiO_2_ being the native layer on the Si surface) and processed the system by irradiating the Au surface by one laser pulse at a wavelength of 532 nm, a pulse duration of 12 ns and a laser fluence increasing from 0.5 J/cm^2^ to 1.5 J/cm^2^.

**Figure 13:**
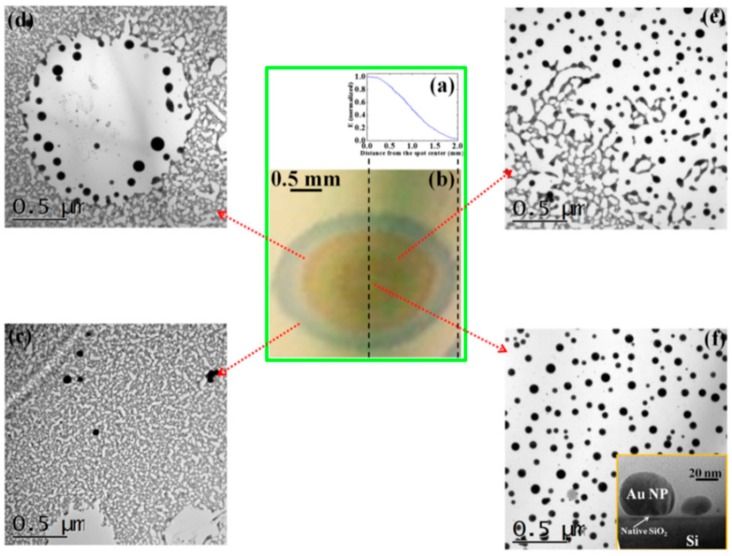
(**a**) The gaussian intensity profile for the laser used by Ruffino et al. [45] (laser wavelength = 532 nm, pulse duration = 12 ns). (**b**) Optical photograph of the laser spot on the Au film/SiO_2_ substrate laser-processed by 1 J/cm^2^. (**c**–**f**) Plan-view transmission electron microscopy images taken in the sample irradiated by 1000 mJ/cm^2^ at increasing distances from the center of the laser spot: (**c**) >600 μm, (**d**) between 600 and 300 μm, (**e**) at about 300 mm, (**f**) <300 mm. The inset in (f) shows a cross-view transmission electron microscopy image to highlight the shape of the formed nanoparticles. Reproduced with permission from [45]. Copyright Elsevier, 2012.

The authors [45] observed that the laser spatial intensity profile is Gaussian (as reported in Figure 13a). Due to this gaussian profile, the fixed value for the laser fluence is a circular area of 600 μm in diameter around the center of the laser spot. Outside from this circular region, the laser fluence decreases according to the intensity Gaussian profile till reaching zero at the laser spot edge. Figure 13b reports an optical photograph of the resulting laser spot on the Au film produced by a laser pulse of fluence, 1 J/cm^2^: different colored regions can be identified as characterized by a decreasing laser intensity, increasing the distance from the center (corresponding to the higher value for the laser intensity).

**Figure 14:**
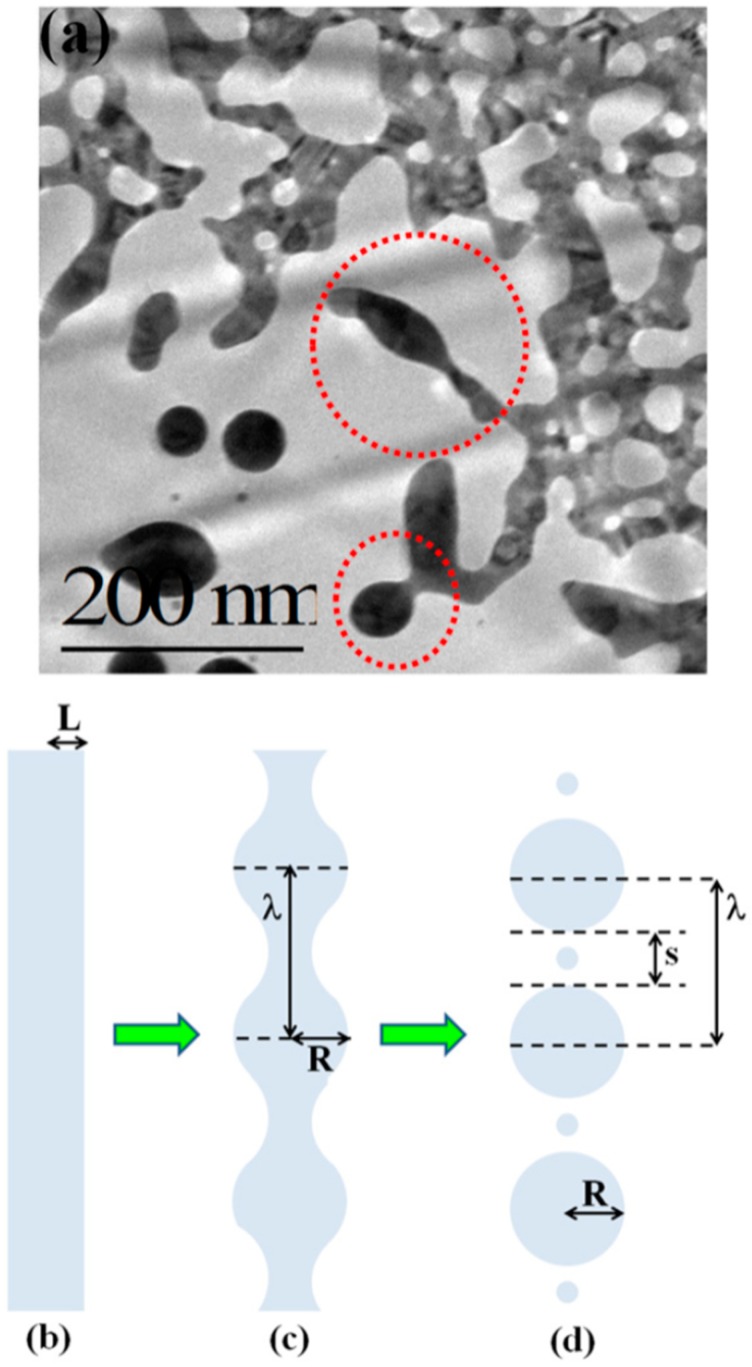
(**a**) The enlarged plan-view Transmission Electron Microscopy (TEM) image taken at about 300 mm from the center of the spot to highlight the formation of nanoparticles from wires. (**b**–**d**) The scheme of the decomposition of an infinite liquid cylinder into an ensemble of particles via a Rayleigh instability. Reproduced with permission from [45]. Copyright Elsevier, 2012.

Then, Figure 13c–f present the Transmission Electron Microscopy (TEM) images acquired in these different regions of the laser spot, i.e., increasing the laser fluence from the spot edge to the center of the spot: (c) >600 μm, (d) between 600 and 300 μm, (e) at about 300 μm, (f) >300 μm. For a low laser fluence (spot edge), the Au film is almost unaltered (Figure 13c). At enough high laser fluence, the film melts and dewets into nanoparticles (Figure 13d). The dewetting process can be identified: in this region, the nucleation of holes is evident. The Au liquid retraction velocity was estimated as v ~ 250 m/s. The coalescence process of the holes giving origin to metal filaments can be recognized in Figure 13e. These thermodynamically unstable filaments split into nanoparticles due to the Rayleigh instability, as can be recognized in Figure 14a. Regarding the Rayleigh instability process, the problem of capillary instabilities driven by surface energy minimization was studied by Lord Rayleigh [44,45,90], finding that a free non-viscous liquid cylinder is unstable to perturbations with wavelengths λ > 2πL, L being the cylinder radius (Figure 14b). So, the unstable cylinder spontaneously splits into liquid droplets which, then, after cooling, solidify (Figure 14c,d). Figure 14b–d shows a schematic picture of the process. It shows sinusoidal thermal perturbations in a liquid infinite cylinder. Increasing time, the fastest growing wavelength will overcome the slower ones and will determine the size of the resulting droplets. In fact, this wave establishes a positive and negative curvature in the cylinder, producing differences in the cylinder radius along its axis. These differences result in a pressure gradient further promoting the growth of the perturbation. On the other hand, the pressure gradient (pinched regions have higher pressure than the bulging regions) produces a fluid flux causing, finally, the pinched areas to rupture and the transformation of the bulged regions into the spherical particles. The calculations show that the perturbations with a wavelength λ = 9.016L dominate the process. Nichols and Mullins [44,45,91,92,93] adapted the original work by Lord Rayleigh to the case of the instability of solid circular cylinders without any contact with other materials [91].

**Figure 15:**
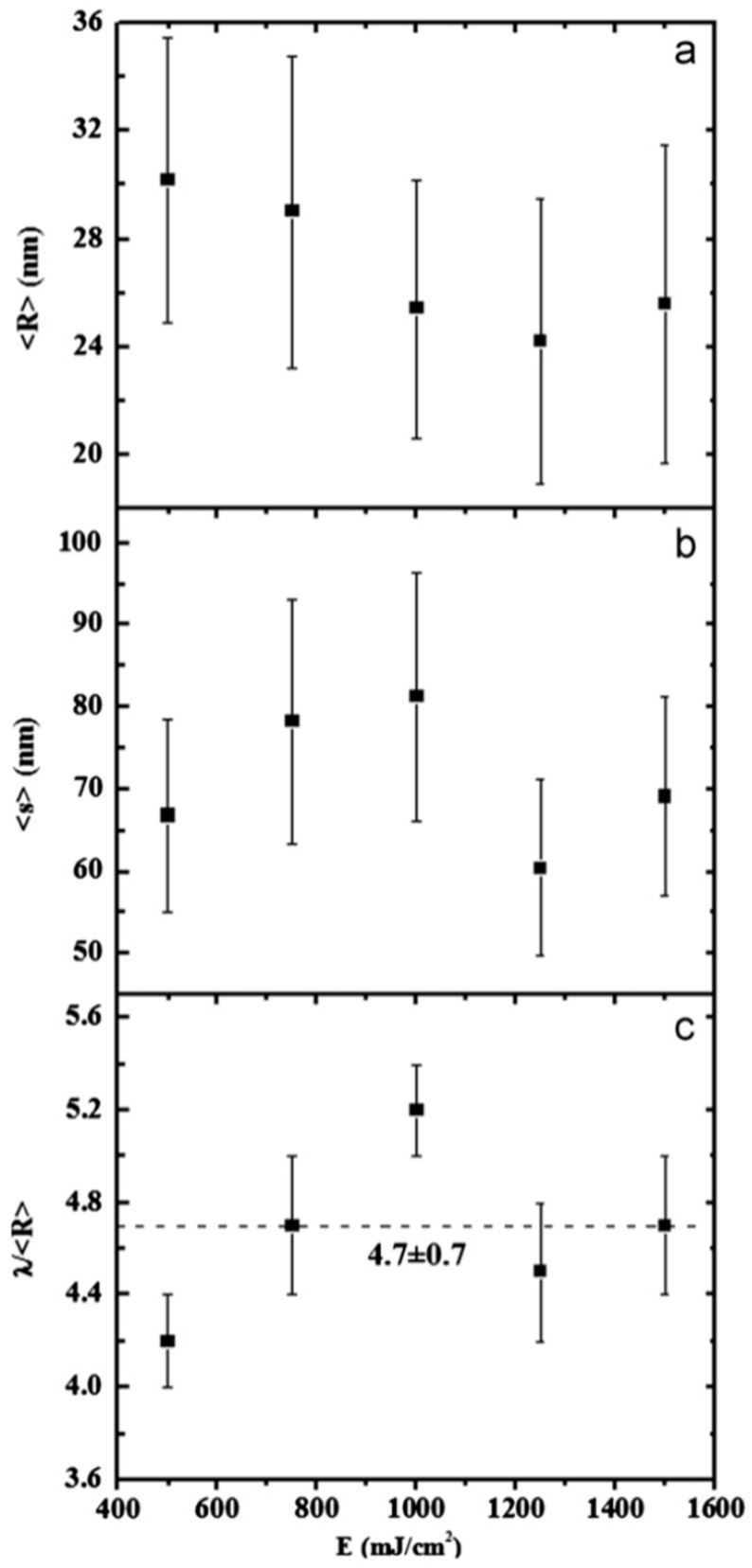
The evolution of the average Au nanoparticles radius < R > (**a**) and average surface-to-surface distance < s > (**b**) versus the laser fluence E. (**c**) The evolution of the ratio (λ/< R >) = (< s > + 2 < R >/< R >) versus the laser fluence E. Reproduced with permission from [45]. Copyright Elsevier, 2012.

In this case, the authors found that the wavelength of the dominant perturbations depends on the specific mass-transport mechanism. For surface diffusion, the dominant perturbations are those having λ = 8.89L [91,92,93]. The calculations show, in addition, that the diameter of the split droplet is [91,92,93] D = 3.78L. Thus, for the droplets originating from the cylinder decay, the theory predicts λ/D = 4.7. Ruffino et al. [45] interpreted λ as the average center-to-center distance between the dewetted nanoparticles, which was quantified by the TEM images as the average nanoparticles’ diameter < D >. So, they reported the average nanoparticles < D >, the average surface-to-surface nanoparticles distance < s >, and the ratio (λ/< R >) = (< s > + 2 < R >/< R >) versus the laser fluence E (Figure 15). In particular, the experimental data in Figure 15c show that (λ/< R >) = 4.7 ± 0.7 independent on the laser fluence in agreement with the predicted value. This is a strong indication for the Rayleigh instability phenomenon as the leading pathway for the dewetting process.

**Figure 16:**
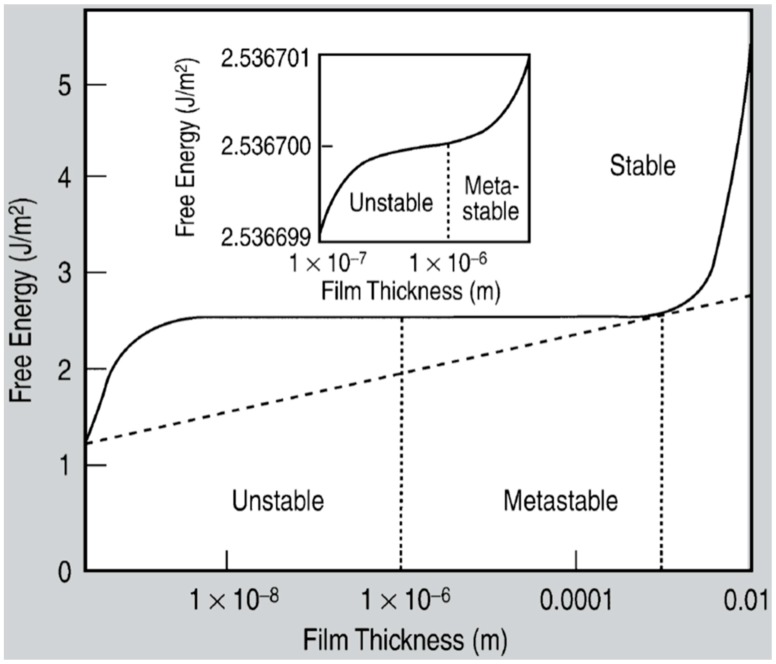
The free energy curve of a metallic film deposited on a non-metallic substrate. Three distinct stability regions can be identified for the film on the basis of the film thickness, named the unstable, metastable, stable thickness regimes. Typically, metal films are unstable in the thickness range 0–1 μm, metastable in the thickness range 1 μm–1 mm, while films with a thickness larger than 1 mm are stable. The inset is a magnified image showing the inflexion point that differentiates the unstable and metastable regions. Reproduced with permission from [37]. Copyright Springer, 2008.

**Figure 17:**
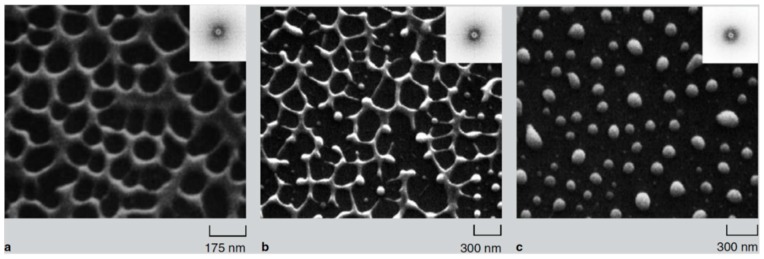
The SEM micrographs presenting the characteristic steps of the morphological evolution of a dewetting 3.5 nm-thick Fe film after pulsed laser irradiation (a wavelength of 266 nm, pulse duration of 9 ns, repletion rate of 50 Hz, a fluence higher than the threshold for melting): (**a**) 5 pulses, (**b**) 500 pulses, (**c**) 10,000 pulses. The fast Fourier transform in the inset of each of the morphological steps depict the short-range spatial order present during each stage of dewetting. Reproduced with permission from [37]. Copyright Springer, 2008.

**Figure 18:**
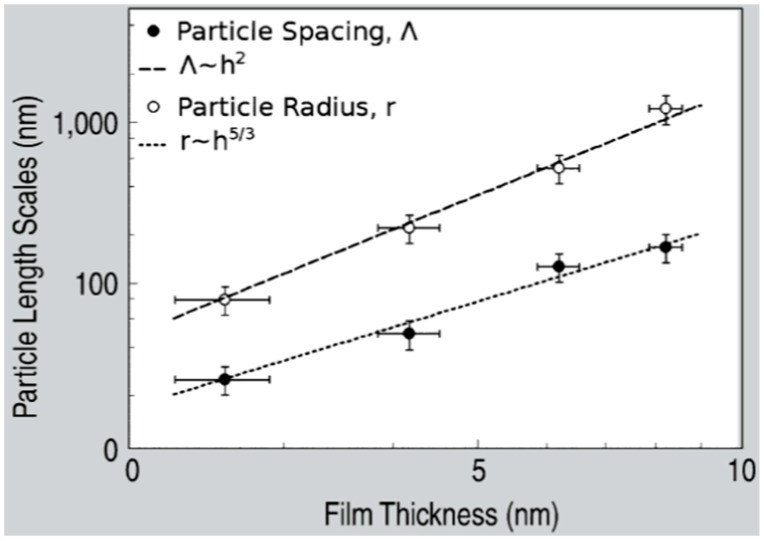
The plot (Log-Log scales) of the Fe nanoparticle size (r) and spacing (Λ) versus the initial thickness of the deposited Fe film. Dots are experimental data while the lines are the fit of the experimental data by r∝d^5/3^ and Λ∝d^2^ (in the figure legend, the film thickness d is indicated by h). Reproduced with permission from [37]. Copyright Springer, 2008.

The laser-induced dewetting process of thin metal films on substrates is largely regarded as an effective method for the spontaneous formation of two-dimensional arrays of nanoparticles by self-organization [25,26,27,28,29,30,31,32,33,34,35,36,37,38,39,40,41,42,43,44,45,46,47,48,49,50,51,52,53]. As early stated, the driving force for the spontaneous dewetting of continuous films in droplets is the minimization of the total surface energy of the system which, at constant volume, is lower for a system of spherical droplets on a flat surface than for the continuous film-substrate system [37]. For film-substrate systems showing this tendency, the plot of the system-free-energy versus the film thickness shows a very similar behaviour to the composition-dependent behavior in two-phases systems characterized by spinodal phase segregation (Figure 16) [37]. Thus, such systems are, often, referred to dewet by spinodal dewetting [37,78,79]. The total free energy of a substrate-film system can be expressed as [37] G(d) = G_surf_ + G_int_ + G_vol_ + G_ext_, which is dependent on the thickness d of the film. In this expression, G_surf_ is the surface energy of the film (in contact with vacuum), i.e., the film surface tension γ_F_; G_int_ represents the film substrate interface energy, i.e., the film-substrate interfacial tension γ_F/S_; G_vol_ is the volume free energy which takes a specific functional form corresponding to the specific system under analysis; G_ext_ = (1/2)ρgd^2^ is the gravitational energy of the film (ρ: the film density), which is, usually, negligible for nanoscale-thick films with respect to the other terms. Regarding, in particular, metal films on inert substrates (as typically on SiO_2_) G_vol_ is, mainly, due to the film intermolecular dispersion forces which are actually the van der Waals interactions between non-polar atoms [37]. In this case, the theory shows that [37] G_vol_ = A/h^2^ A being the Hamacker coefficient representing the sign and the magnitude of interaction between the film and the substrate and between the film and vacuum. In the case of metal films on insulating substrates (as SiO_2_) in a gaseous or vacuum environment, the resulting free energy is attractive. Furthermore, if the thickness of these films is, typically, in the 1–100 nm range, G_xet_ is negligible, and, observing Figure 16, the derivative of the G (d) curve is <0, indicating a thermodynamically unstable regime for the film. In this unstable regime, if material diffusion can occur, the film spontaneously tends to break-up by the first step of the nucleation of holes in the film due to the amplification of the film thickness inhomogeneities. Interestingly, however, in the successive evolution of the dewetting process (leading to the formation of droplets), the characteristic length scales arise since the dewetting dynamics will be dominated by the fastest growing length scale Λ [37,94,95,96] as seen when the process evolves by the Rayleigh instability mechanism. Theoretical calculations [37,96,97,98,99] shows that the characteristic length Λ scales with the film thickness d as Λ∝d^2^ which was experimentally verified by several studies confirming a spinodal-like self-organization process for liquid films [37]. On the other hand, when the film thickness is d > 100 nm (metastable region; see Figure 16), the dewetting process starts with the formation of holes by homogeneous nucleation in random spatial positions so that the resulting film morphology lacks the characteristic length scales. To study the characteristics of morphology and the pattern of spinodal-like dewetting films, Krishna et al. [37] deposited Fe films on the SiO_2_/Si substrate, increasing the film thickness, however, also maintaining the film thickness below 10 nm. Then, these films were laser processed by laser pulses with a wavelength of 266 nm, a pulse duration of 9 ns, a repletion rate of 50 Hz, with a fluence higher than the threshold for melting and increasing the number of pulses. Some representative resulting morphologies of 3.5 nm-thick Fe film processed by 5 (a), 500 (b), 10000 (c) pulses are reported in Figure 17. As recognizable by the figure, after 5 pulses, the dewetting morphology is characterized by a cellular web of polygons (Figure 17a). By increasing the number of pulses, the metal retracted to the edge of the holes, resulting in an array of coalescing polygonal holes (Figure 17b). Further increasing the number of pulses leads to the formation of nanoparticles preferentially at the junctions of the polygons, as evident in Figure 17b,c. At every observed stage, a characteristic length scale is present, as evidenced by the annular form of the power spectrum of the spatial correlations in the intensity variation within each pattern (presented as an insert in each image in Figure 17). For patterns consisting of polygons, the characteristic length scale represents the mean distance between the centers of the polygons; for the nanoparticles, it represents the interparticle spacing. In this last case, the short-range spatial order indicates the spinodal-like nature of the dewetting process. For spinodal-like dewetting, the theory predicts for the nanoparticles’ radius r and interparticles’ spacing Λ evolutions with the film thickness (d) relations as r∝d^5/3^ and Λ^2^ [33,37,98,100,101]. The authors, then, verified these predictions by plotting (Figure 18) the experimentally-extracted mean radius r and spacing Λ for the Fe nanoparticles versus the starting thickness of the deposited Fe film, finding excellent accordance. Similar results were found for other metals. Trice et al. [33] were able to exploit hydrodynamic pattern formation and dewetting, resulting from the pulsed-laser-induced melting of nanoscale-thick Co films to produce two-dimensional spatially ordered metal nanoparticle arrays on SiO_2_/Si substrates.

In particular, they investigated the pattern formation for the Co film with a thickness equal or lower than 7 nm, which is lower than the Co absorption length for the used laser pulse (a wavelength of 266 nm, repletion rate of 50 Hz, pulse duration of 9 ns) which was evaluated in about 11 nm.

The pattern formation was investigated as a function of the laser energy density and number of pulses. The pattern formation, as usual, was only observed to occur for a laser energy density higher than a critical value (threshold energy for melting) dependent on the Co film thickness. The authors, in addition, developed a model [33] which predicts that spontaneous perturbations on the metal film thickness (i.e., natural roughness) would result in intrinsic thermal gradients ∂T/∂h, with h being the film thickness. A critical thickness h_c_ (≈9 nm) is evaluated by the model so that (∂T/∂h) > 0 for h < h_c_ and (∂T/∂h) < 0 for h > h_c_. Experimentally, the spacing between the dewetted nanoparticles and the particle diameter were found to increase as h^2^ and h^5/3^, respectively. Overall, the dewetting process is caused by the hydrodynamic instability arising when attractive intermolecular forces (as van der Waals forces) between the atoms forming the film become larger than the film-substrate of interfacial tension which provides the stabilizing effect. Under such conditions, an amplification of the film thickness fluctuations spontaneously occurs, eventually leading to film break-up and the formation of particles with a well-defined spatial order.

**Figure 19:**
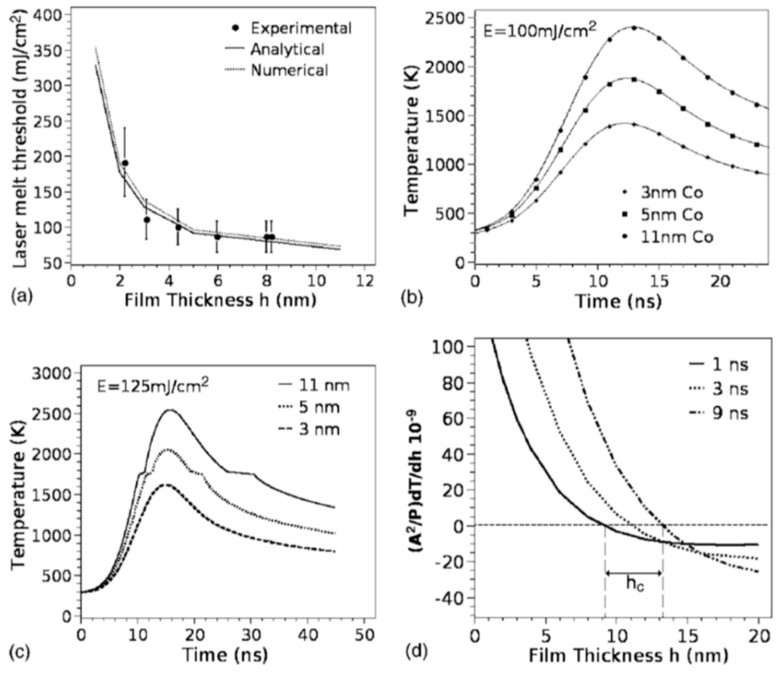
(**a**) The plot of the laser energy density threshold for melting Co films on SiO_2_ versus the film thickness. The plot shows the comparison of experimental measurement (solid circles) with calculations. (**b**) The calculated temporal profiles’ temperature obtained (using temperature independent parameters) for Co films of different thicknesses on SiO_2_ under irradiation with 100 mJ/cm^2^. (**c**) The calculated temporal profiles’ temperature obtained for Co films of different thicknesses on SiO_2_ (under 125 mJ/cm^2^) in the model including the phase change and temperature-dependent parameters. (**d**) The thermal gradient ∂T/∂h calculated from the thermal model whose magnitude and sign were dependent on the film thickness and time to melt (1, 3, or 9 ns) during the film heating. Reproduced with permission from [33]. Copyright American Physical Society, 2007.

**Figure 20:**
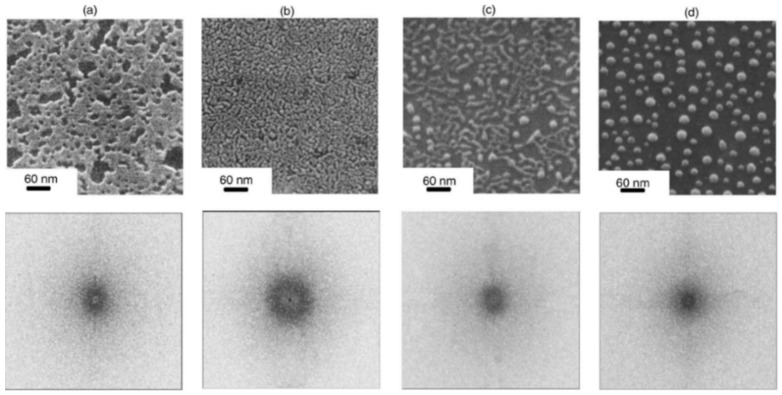
Top row: the SEM images showing the dewetting pattern evolution for a 2 nm-thick Co film irradiated (pulse duration 9 ns) at 200 mJ/cm^2^ as a function of the number of pulses. The bottom row shows the power spectrum images corresponding to the SEM images in the top row. All the power spectra have an annular structure, indicating a band of spatial frequencies and implying a short-range spatial order. In particular: (**a**) 10 pulses, (**b**) 100 pulses, (**c**) 1000 pulses, and (**d**) 10 500 pulses. Reproduced with permission from [33]. Copyright American Physical Society, 2007.

**Figure 21:**
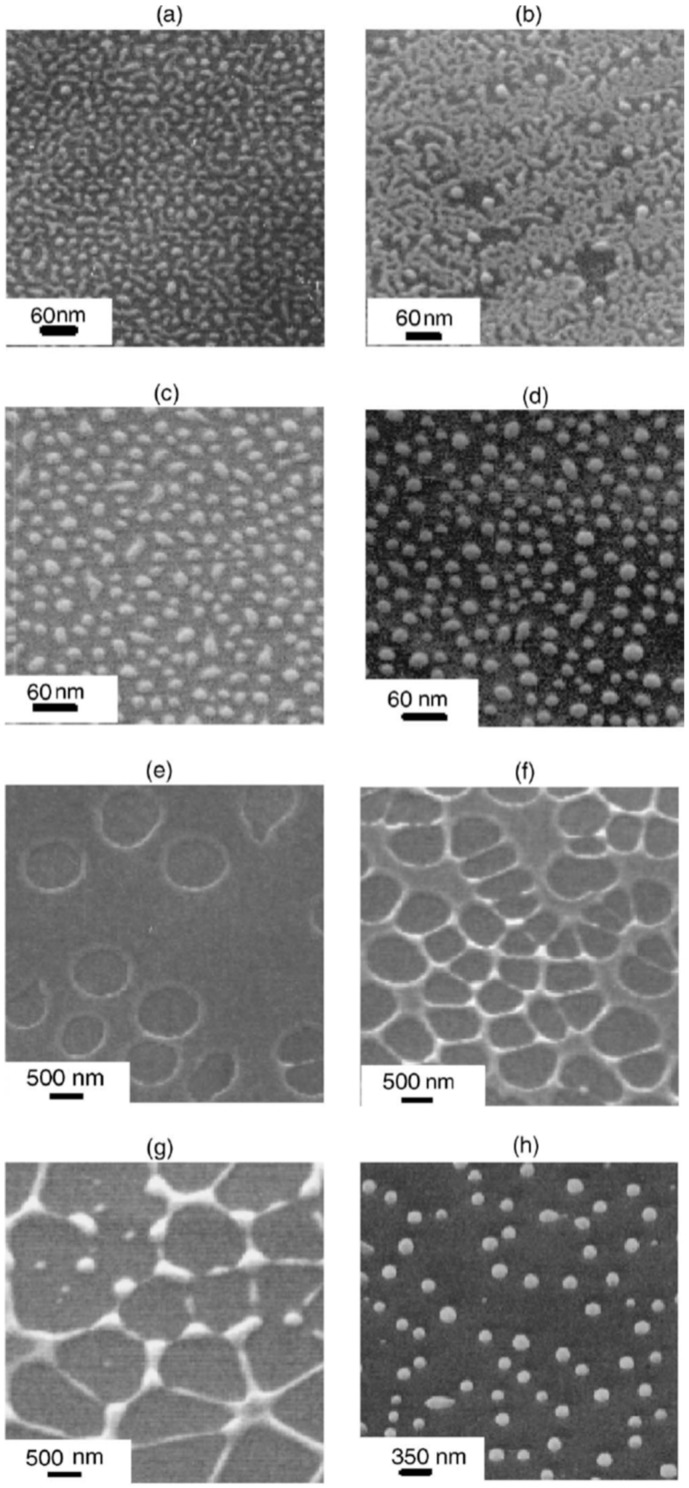
The SEM images of (**a**–**d**) a 2-nm-thick Co film after 100 pulses each of (**a**) 190 mJ/cm^2^, (**b**) 200 mJ/cm^2^, (**c**) 220 mJ/cm^2^, (**d**) 250 mJ/cm^2^; (**e**–**f**) 4.4 nm-thick Co films after irradiation with a fluence of 93 mJ/cm^2^ but increasing the number of pulses as (**e**) 10 pulses, (**f**) 100 pulses, (**g**) 1000 pulses, (**h**) 10500 pulses. Reproduced with permission from [33]. Copyright American Physical Society, 2007.

According to the theoretical model developed by the authors and their experimental data [33], Figure 19 reports (a) the evolution of the laser energy density threshold for melting Co films on SiO_2_ versus the film thickness. The plot shows the comparison of experimentally measured values (solid circles) with calculations; (b) the calculated temporal profiles temperature obtained (using temperature independent parameters) for Co films of different thicknesses on SiO_2_ under irradiation with 100 mJ/cm^2^; (c) the calculated temporal profiles’ temperature obtained for Co films of different thicknesses on SiO_2_ (under 125 mJ/cm^2^) including the phase change and temperature-dependent parameters in the model; (d) the thermal gradient ∂T/∂h predicted from the thermal model whose magnitude and sign were dependent on the film thickness and time to melt (1, 3, or 9 ns) during the film heating. From the experimental point of view, the authors [33] conducted a detailed study on the dewetting morphology for various laser energies as a function of the laser number of pulses. They found that for Co films with a thickness in the range of 3–7 nm (Figure 20 and Figure 21), the typical patterns are formed by separated holes at the early stages of irradiation, followed by a cellular network at the later stages, and finally, nanoparticles which continue to remain stable upon further irradiation.

On the other hand, for films with a thickness lower than 3 nm (Figure 20 and Figure 21), the morphology appears as discrete holes followed by a bicontinuous structure and followed by a final state characterized, again, by nanoparticles. The general theory of the dewetting process [33,102] predicts three main underlaying mechanisms: (1) the homogeneous nucleation and growth in which holes are formed randomly in the location and time on the film surface, therefore, no characteristic length is present in this type of dewetting [103]; (2) heterogeneous nucleation and growth due to defects, impurities, or more general film heterogeneities and, in this case, the early stages of dewetting could establish a characteristic length scale in the dewetting pattern due to the ordered nucleation sites; (3) thin film hydrodynamic instabilities (as in the case of spinodal dewetting unstable systems and, in this case, the resulting patterns present a well-defined length scale in the holes spacing and size. In this regard, Figure 20 reports the morphology of a 2 nm-thick Co film as a function of the increasing number of laser pulses at a fluence of 200 mJ/cm^2^. Separated holes are visible after a low number of pulses (Figure 20a) with the pattern evolving to a bicontinuous structure (Figure 20c,d) and, finally, into nanoparticles (Figure 20d). A comparison of the density of features in Figure 20c,d indicates that the nanoparticles originate from wires. Figure 21a–d shows the pattern morphology after 100 pulses as a function of laser fluence for the 2 nm-thick Co film. The general characteristics of the morphology were similar to those observed as a function of the pulses number, as shown in Figure 20. Figure 21e–h presents the morphology of a 4.4 nm-thick Co film as a function of the number of laser pulses at a fluence of 93 mJ/cm^2^. Discrete holes are visible after the lowest number of pulses (Figure 21e), with the pattern evolving to a cellular web (Figure 21f) as the number of holes increases. Further increasing the number of pulses causes the retraction of the metal towards the edge of the holes so that the pattern evolves to large polygonal structures with evidence for particle formation preferentially at the vertices of the polygons. This is more evident in Figure 21g. After a high number of pulses, stable (against continued irradiation) nanoparticles were observed (Figure 21h).

**Figure 22:**
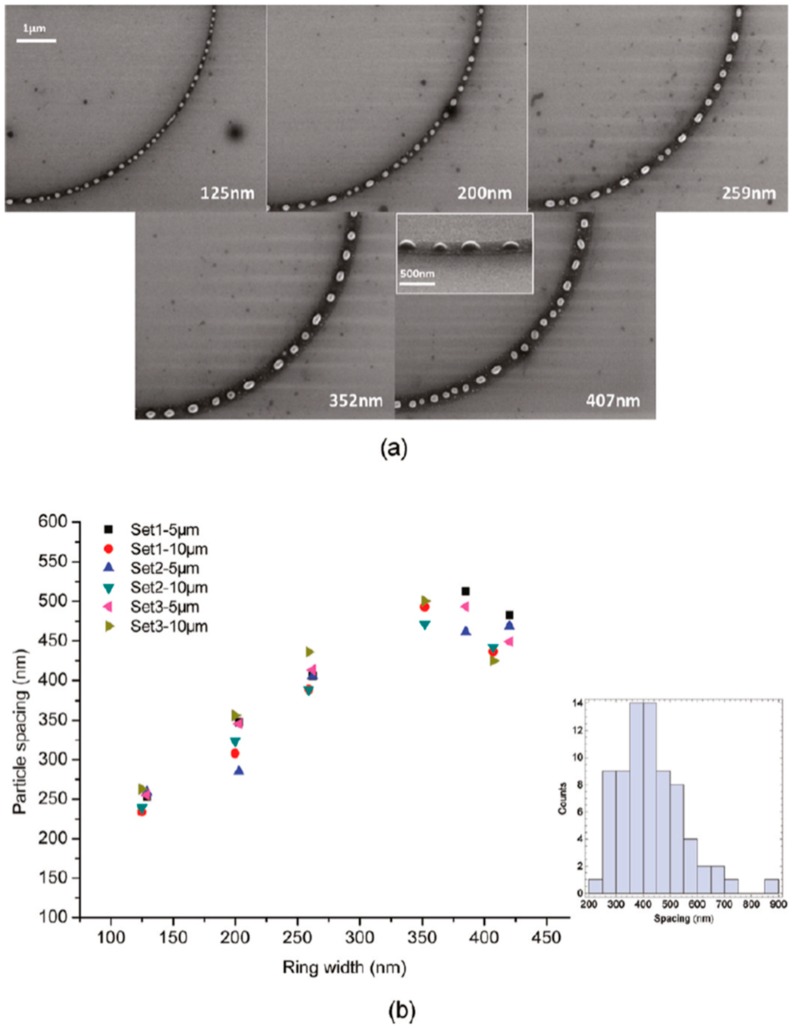
(**a**) The SEM images of the 7.8 nm-thick Cu ring with a radius of 5 μm and a variable width (125 nm, 200 nm, 259 nm, 352 nm, 407 nm, as indicated in the corresponding images) after 5 pulses (248 nm wavelength, 18 ns pulse duration, 160 mJ/cm^2^ fluence). Inset: a 60° tilted SEM image of a portion of the corresponding ring. (**b**) The plot of the mean Cu nanoparticles spacing for fifteen 5 μm-radius and 15 fifteen 10 μm-radius rings as a function of the measured widths. The inset shows the histogram of the droplet spacing (lower right) for 407 nm wide rings of 5 μm radius). Reproduced with permission from [42]. Copyright American Chemical Society, 2011.

**Figure 23:**
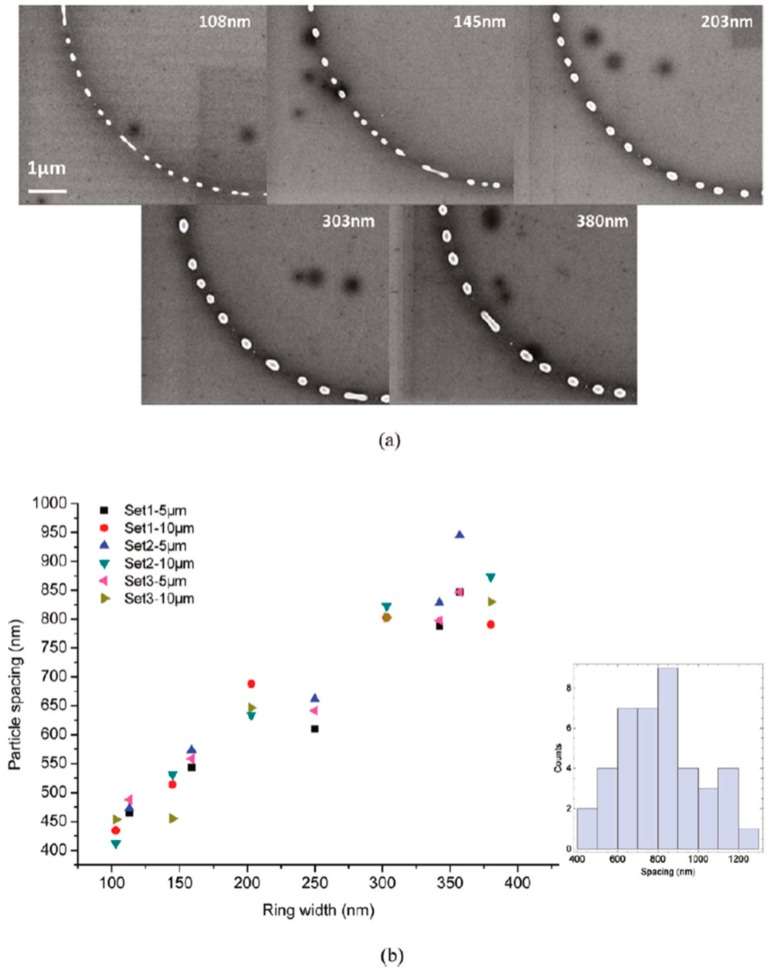
(**a**) The SEM images of the 15 nm-thick Cu ring with a radius of 5 μm and a variable width (108 nm, 145 nm, 203 nm, 303 nm, 380 nm, as indicated in the corresponding images) after 5 pulses (248 nm wavelength, 18 ns pulse duration, 160 mJ/cm^2^ fluence. (**b**) The plot of the mean Cu nanoparticles spacing for 15 fifteen 5 μm-radius and 15 fifteen 10-μm radius rings as a function of the measured widths. The inset shows the histogram of the droplet spacing (lower right) for 380 nm wide rings of 5 μm radius.). Reproduced with permission from [42]. Copyright American Chemical Society, 2011.

**Figure 24:**
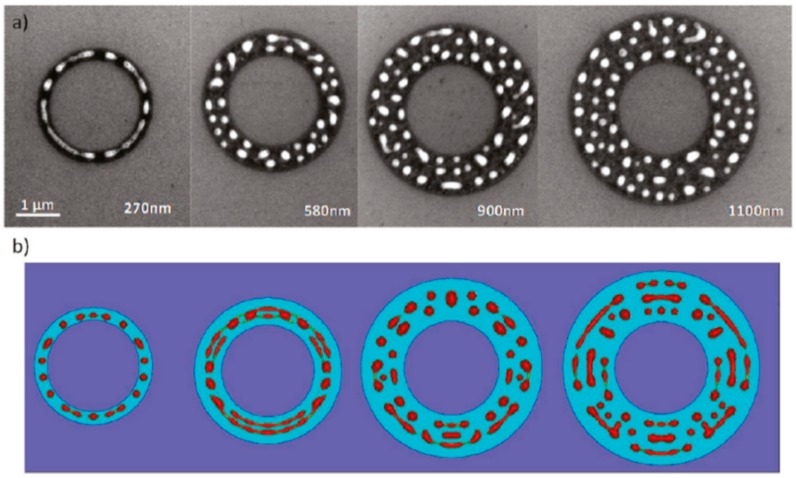
(**a**) The SEM images of the 7.8 nm-thick, 1 μm radius copper rings of variable ring widths (270, 580, 900, 1100 nm). (**b**) Snapshots of nonlinear 2D simulations of these rings in (**a**) at t = 100 ns (the light blue background shows the original ring. Reproduced with permission from [42]. Copyright American Chemical Society, 2011.

**Figure 25:**
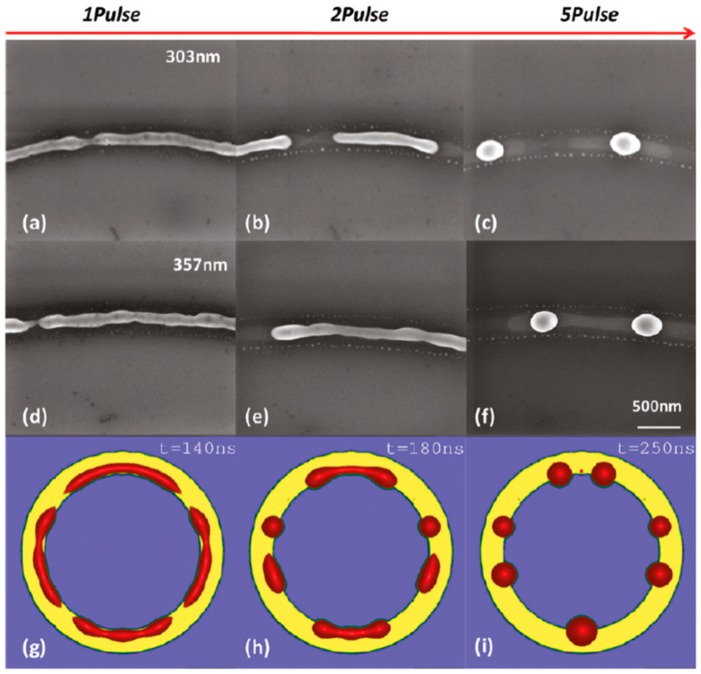
The SEM images of the 15 nm-thick rings ((**a**–**c**) 303 nm wide and (**d**–**f**) 357 nm wide) laser processed increasing the number of pulses and illustrating the circumferential mass transport competing with the instability of growth and leading to larger than predicted length scales. (**g**–**i**) 2D simulations of a 350 nm wide ring at different liquid lifetimes which illustrates that the fastest growing modes pinch off and subsequently coarsen the original instability length scale. Reproduced with permission from [42]. Copyright American Chemical Society, 2011.

Wu et al. [42] exploited the laser-induced dewetting approach for the nanostructuration of patterned metal films on substrates, i.e., to produce, on the surface, two-dimensional arrays of metal nanoparticles with specific spatial arrangements. In particular, they produced, on 100 nm-thick SiO_2_ deposited on Si, nanoscale-thick Cu rings by the electron beam lithography approach. Cu rings with two thicknesses (7.8 and 15 nm), two radii (5 and 10 μm), and variable ring widths (ranging from 103 to 420 nm) were patterned. These continuous Cu rings were melted by laser irradiations using five pulses, a laser wavelength of 248 nm, a pulse duration of 18 ns, and a laser fluence of 160 mJ/cm^2^ (higher than the threshold for melting). Figure 22 and Figure 23 show the formation of ordered rings of Cu nanoparticles whose spacings (and sizes) increase smoothly and monotonically by increasing the ring width. Such an increase of the average spacing with the ring width should be determined by a ring evolution dominated by the Rayleigh instability (i.e., the contraction of the ring followed by a breakup in droplets) [104]. However, the authors, crossing experimental and theoretical investigations, found some confirmations and some discrepancies for the Rayleigh instability as the leading driving phenomenon. In fact, the authors developed models to evaluate the typical time scales for different processes (thin film instability, Rayleigh instability) and the influence of these time scales on ring dewetting was analyzed for different initial thicknesses of the ring with the results summarized in the following:

a) 7.8 nm-thick ring: for the width of the ring lower than or equal to 350 nm, the mean spacing between the formed Cu nanoparticles increases by increasing the ring width (see Figure 22) and basically follows the length scale as expected from the Rayleigh instability mechanism. However, for a ring width larger than 350 nm, the data in Figure 22 suggest a saturation regime followed by the particles spacing decreasing the bu, further increasing the ring width. However, the theoretical calculations show that for the 100 nm wide ring, the time for the molten ring to reach the equilibrium shape is ~2 ns, which is less than the thin film instability time calculated in 6.5 ns. So, the dynamics of the molten ring formation is faster than the thin film instability and the subsequent molten ring is expected to decay according to the Rayleigh instability. For rings with a width of 300 and 500 nm, the calculated time scale for thin-film instability is shorter than the calculated retraction time. However, the difference is not very large and, therefore, the Rayleigh instability can be considered the dominant mechanism determining the distance between the Cu droplets. To further extend the conclusions for the 7.8 nm-thick rings, the authors [42] also fabricated rings with a width ranging from 270 nm to 1100 nm and a radius of 1 μm (see Figure 24). In this case, the authors observed a transition from a single rivulet to several concentric rings of nanoparticles, as recognizable both from the experimental SEM images and the two-dimensional (2D) simulations in Figure 24. In particular, the 2D numerical simulations shown in Figure 24b indicate that the original ring evolves into two rings which subsequently decay in droplets following the Rayleigh mechanism.
b) 15 nm-thick rings: in this case, the rings are predicted by the author’s model to decay due to the Rayleigh instability and this prediction is in general agreement with the experimental trend observed in Figure 25. However, the experimental average spacing for the obtained nanoparticles is larger than the expected one considering only the Rayleigh mechanism. Figure 25, in particular, reports a comparison of the experimental observations and 2D numerical calculations to draw further information on the involved phenomena. In the experimental part (Figure 25a–f), the instability evolution can be observed to increase the number of laser pulses by increasing the ring width (303 and 357 nm). In the simulation row (Figure 25g–i), the results of the 2D simulations carried out for the ring width of 350 nm and radius of 1 μm radius are reported. The distance between the drops in the experiments (see Figure 25c–f) appears large compared to the wavelength of the fastest growing perturbations which can be seen in Figure 25a,d.

This is due to the fact that the starting ring initially breaks into sections at a few locations (four in the example shown in Figure 25h), leading to rivulets longer than the wavelength of the perturbation growing at the highest rate. These rivulets, if long enough, consequently decay into droplets. However, if the rivulets are not sufficiently long (about twice the maximum wavelength of the growing perturbations’ maximum), they may decay just into a single particle.

Overall, for both the thin and thick rings, the distance between the particles is larger than the one predicted by the Rayleigh mechanism. This difference between the experimental results and the prediction of the Rayleigh model is particularly significant for thicker rings and this difference is imputated by the authors to nonlinear effects combined with the fast-circumferential transport.

These results by Wu et al. [42], then, throw new light on the previous results by Henley et al. [27] and Trice and al. [33]: even if hydrodynamic film instabilities are the main reason for the film dewetting, the results of Wu et al. [42] show, also, the necessity, in particular conditions (thicker rings) to invoke additional (nonlinear) mechanisms affecting the metal films’ dewetting process.

**Figure 26:**
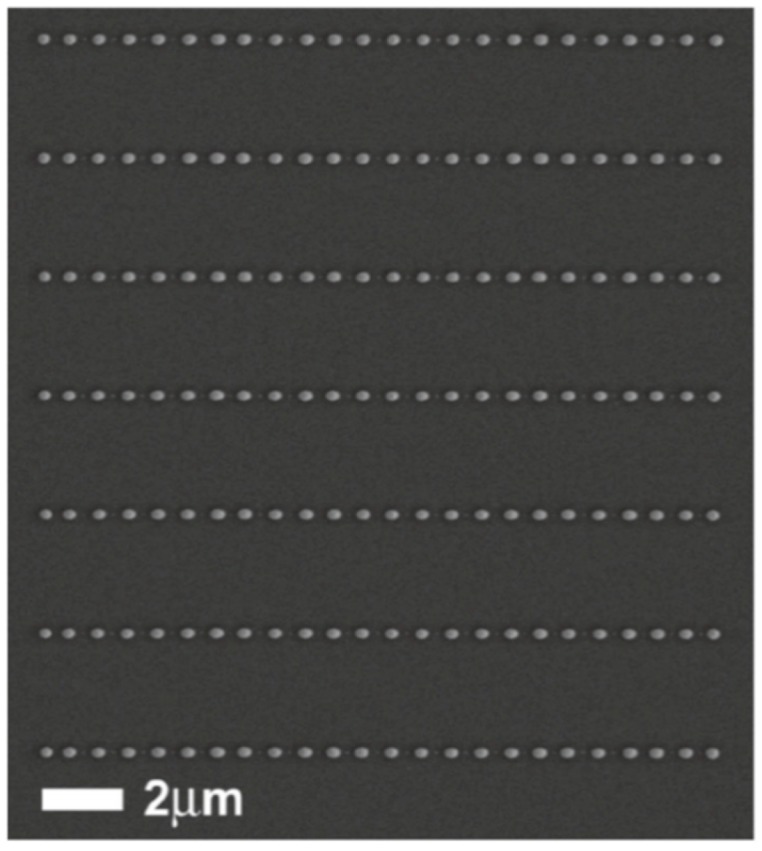
The array of Ni nanoparticles (on Si substrate) arranged in seven lines and obtained by pulsed laser irradiations (wavelength of 248 nm, pulse duration of 18 ns, fluence of 400 mJ/cm^2^, five pulses) of seven nanoscale-thick Ni patterned on the substrate by electron beam lithography. Reproduced with permission from [43]. Copyright American Chemical Society, 2011.

**Figure 27:**
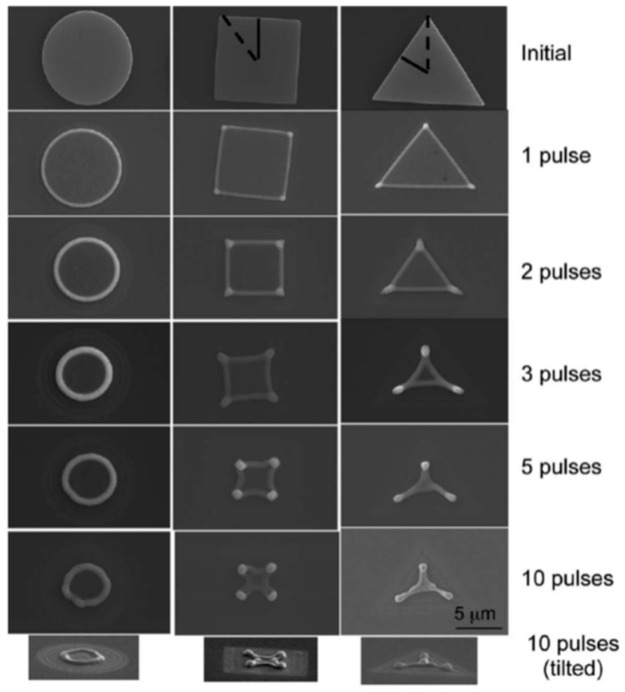
The SEM images of a pulsed laser (wavelength of 248 nm, pulse duration of 25 ns, fluence of 420 mJ/cm^2^) treated thin Ni patterns on the Si substrate. The top images are the initial thin film circle, square and triangle. Subsequent SEM images in each column show the patterns’ evolution after 1, 2, 3, 5, and 10 laser pulses. The bottom image is a tilted view of the pattern after 10 laser pulses. The dashed lines on the top square and triangle illustrate an axis of the lateral contraction from the vertices and the solid lines, indicating the axes from the center of the edges. Reproduced with permission from [105]. Copyright American Institute of Physics, 2008.

From an experimental point of view, similar approaches to that proposes by Wu et al. were widely used to produce arrays of metal nanoparticles on surfaces with desired spatial configurations as lines of Ni nanoparticles (see Figure 26) obtained by Fowlkes et al. [43] or micro- and nano-patterned complex-morphology Ni structures (see Figure 27) by exploiting the laser-induced dewetting process of patterned films at various intermediate stages as obtained by Rack et al. [105].

## 4. Nanostructuration of Thin Metal Films by Picosecond Pulsed Laser Irradiations

Picosecond-pulsed laser irradiation of metals allows the spatial confined removal (ablation) of the material with high accuracy due to the reduced heat affected region compared to nanosecond-pulsed laser irradiation and this peculiarity can be exploited to produce micro- and nano-sized features on metal targets or deposited metal films.

**Figure 28:**
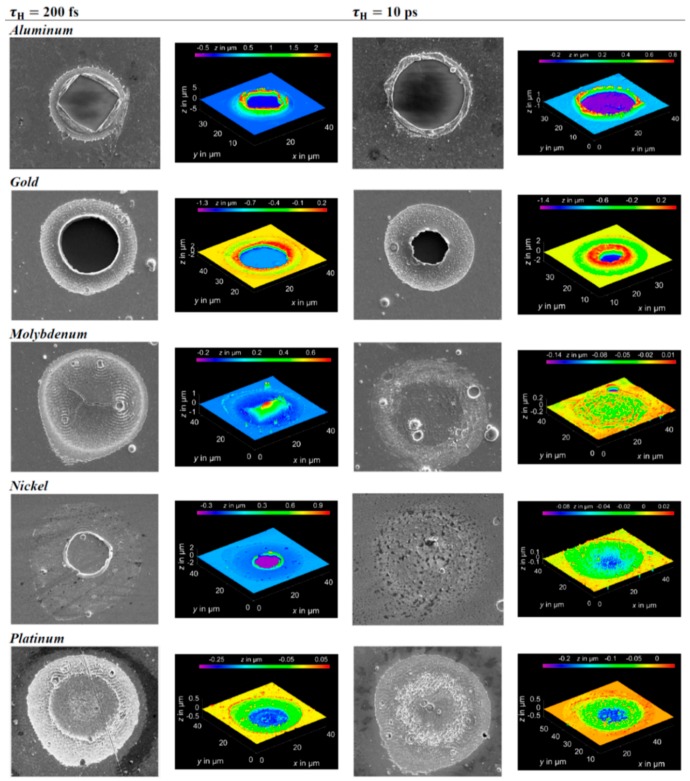
The typical ablation structures obtained for Al (**first row**), Au (**second row**), Mo (**third row**), Ni (**fourth row**), and Pt (**fifth row**) thin films (**deposited on glass**), processed by one laser pulse of energy 53 μJ, with a wavelength of 1028 nm, a pulse duration of 200 fs (**two columns on left**) or 10 ps (**two columns on right**). For each fixed pulse duration, the first column reports the reflection mode of the optical microscopy images, the second column reports the confocal microscopy images. Reproduced with permission from [54]. Copyright Elsevier, 2016.

As an example, Olbrich et al. [54] studied the ablation process of Au, Pt, Al, Ni, Mo films (deposited on glass with a thickness in the 300–2000 nm range) as induced by a single laser pulse with a wavelength of 1028 nm and pulse duration variable in the 200 fs–15 ps range. As we generally discussed in Section 2, as a laser pulse incides on a metal surface, the laser light can be partially reflected and transmitted throughout the surface. The laser intensity decreases within the metal due to absorption of the laser-generated energy by the electrons of the metal and resulting in an excited non-equilibrium state. The hot electrons diffuse within the material and the electron-electron scattering events provide a transfer energy pathway. After the electron relaxation time, the standard Fermi distribution describes the energy distribution for the electrons and the temperature evolution of the electrons and phonons system can be described by the two-temperatures model. In the theoretical and experimental studies by Olbrich et al. [54], the metals Au, Pt, Al, Ni and Mo were chosen since they present very different thermophysical properties, especially their electron-phonon relaxation time; see Figure 3. From a theoretical point of view, the calculations performed by Olbrich et al. [54] (see Figure 4), considering a pulse laser irradiation with a pulse duration of τ_H_ = 200 fs (Figure 4 left) or τ_H_ = 10 ps (Figure 4 right) and a laser energy of 1 μJ and a laser wavelength of 1028 nm, show that for Au, Pt, Al, Ni, and Mo, T_max,e_ (the maximum electronic temperature) is higher for τ_H_ = 200 fs than for τ_H_ = 10 ps. In addition, the highest T_max,e_, for τ_H_ = 200 fs, is reached in Al. On the other hand, the irradiation for τ_H_ = 10 ps causes the highest T_max,e_ in Au and, in any case for Au, the maximum value for T_max, ph_ (the maximum phonons’ temperature) is reached later than the maximum value of T_max,e_. These calculations were supported and compared to the experimental results. In particular, the authors [54] irradiated Au, Pt, Al, Ni, and Mo thin films by a single laser pulse, changing the laser energy for two fixed pulse durations (200 fs and 10 ps) and studied the resulting film morphology modifications. Some results are summarized in Figure 28 which reports on the images showing the morphological effects of the irradiations on all the investigated metals, fixing a laser energy of 53 μJ and for the two different pulse durations. For each pulse duration, the left images report reflection-mode optical microscopy images and the right images report the corresponding confocal microscopy images. In particular, the authors observed in the cases of Au and Al, that the whole film is ablated and the metal structures remaining on the surface and originating from the ablation process are higher at the edges of the modified ones, indicating a delamination of the film around these regions. A possible explanation for the formation of these structures is presented by the authors [54] in the spallation process of a microbump [106,107]. The morphology evidenced by the confocal microscopy images acquired for Pt, Ni, Mo processed by the 200-fs laser pulse appears due to a non-circular intensity distribution of the laser radiation and on the outer edge. In these cases, the ablation depth of this morphology is measured in about 10–20 nm.

The results in Figure 28 evidence, also, a dependency of the morphology of the produced features on the pulse duration (which is confirmed also by the dependence, for each metal, of the laser ablation threshold on the pulse duration which, generally, decreases by increasing the pulse duration apart for Au [54]). Interestingly, the authors observed that the calculated laser energy thresholds for metal melting are lower than that experimentally derived. This difference was ascribed to the fact that small surface layers of molten metal are delaminated from the thin film due to the induced shockwave resulting from the ultrafast expansion of the material under the picosecond laser pulse [108].

An important aspect related to the material irradiation by picosecond-pulsed laser relies in the formation of periodic surface structures. In this sense, for example, the characteristics of picosecond-pulsed laser-induced surface structure on metals (Cu) were studied by Maragkaki et al. [109] referring, in this case, to a Cu bulk target instead of a thin film. However, the obtained results are interesting in view of the interpretation of results for deposited metal thin films.

**Figure 29:**
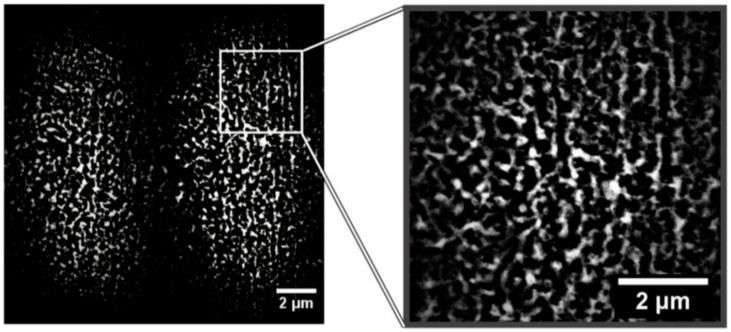
The laser-induced periodic surface structures obtained on the Cu (bulk) target after one laser pulse with a wavelength of 355 nm, a pulse duration of 7 ns, a fluence of 0.4 J/cm^2^. Two irradiation spots with centers separated by about 10 μm can be recognized in the left image. The laser-induced periodic surface structures visible in the right image show an average period of about 300 nm. Reprinted with permission from Reference [109], Elsevier, 2017.

**Figure 30:**
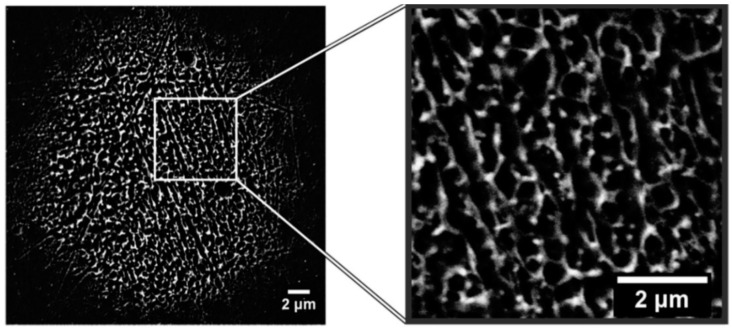
The laser-induced periodic surface structures obtained on Cu (bulk) target after one laser pulse with wavelength = 1064 nm, pulse duration = 7 ns, fluence = 5.5 J/cm^2^. The irradiation spot can be recognized in the left image. The laser-induced periodic surface structures visible in the right image show an average period of about 580 nm. Reproduced with permission from [109]. Copyright Elsevier, 2017.

**Figure 31:**
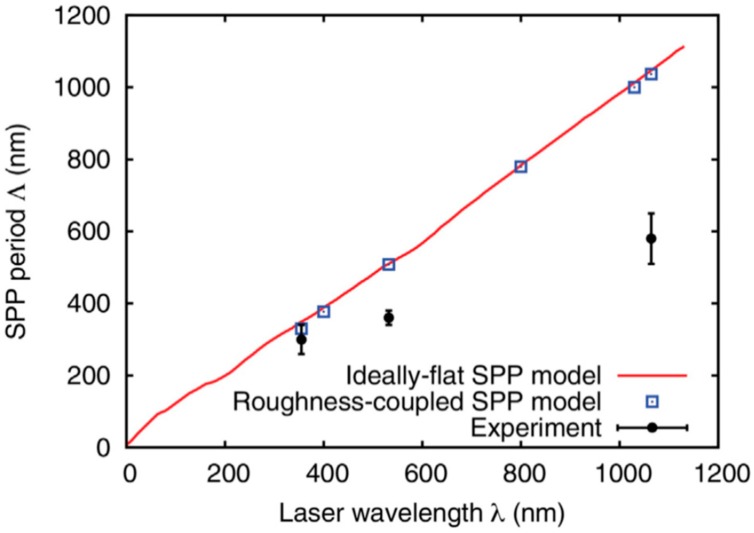
The period of the laser-induced periodic surface structures obtained on Cu (bulk): black dots indicate the experimental values (obtained by a single pulse of the 7 ns-pulsed laser with energy = 5.5 J/cm^2^), the red line indicates the prediction of the model, taking into account the surface plasmon polaritons on a flat metal surface, with the blue squares indicate the predictions of the model, taking into account the realistic surface roughness of the metals surface. Reproduced with permission from [109]. Copyright Elsevier, 2017.

In particular, the authors [109] studied the mechanisms of the laser-induced periodic surface structures’ formation on the Cu surface (chosen due to its higher electrical and thermal conductivity, higher melting temperature, lower rates of diffusivity, and higher strengths than other standard metals). They focused the studies on the change of the average period for the laser-induced periodic surface structures as a function of the wavelength of the incident laser radiation using a 7 ps-pulsed laser. Interestingly, they compared the experimental results with the predictions obtained supposing that the surface-scattered laser waves are the main driving force for the laser-induced periodic surface structures’ formation, finding, however, a considerable disagreement. On the basis of this finding, they suggest, instead, that hydrodynamic mechanisms can be regarded as the main mechanism for the periodic surface structure formation and the compatible with the experimentally-observed pattern periodicity [109]. According to Maragkaki et al. [109], the main theories describing the laser-induced periodic surface structures’ formation are (1) the theory ascribing the laser-induced periodic surface structures’ formation to the interference of the incident laser with the surface scattered wave [110,111]; (2) the self-organization processes on the surface [112,113,114,115,116]. In particular, for metallic surfaces, surface scattered radiation waves are described by surface plasmon polaritons [117] evolving in localized plasmons as the surface roughness increases [118]. In this view, surface roughness act as a coupling means for the incident laser wave with the surface (an effect described by the surface-scattered wave model [110]). However, several experimental evidences [113,114] suggest that, in many situations, the plasmonic-based mechanism cannot solely describe the origin of laser-induced periodic surface structures’ formation without considering additional physical processes. For example, Raman measurements showed that the laser-induced periodic surface structures’ formation takes place in the molten phase [114], indicating the occurrence of the hydrodynamic process in the pattern generation. For multi-pulse irradiation regimes, it was argued that the plasmonic stage, which governs the laser-induced periodic surface structures’ orientation, does not necessarily determine the periodicity of the final pattern due to the contribution of thermocapillary effects [119]. The hydrodynamic processes can be more and more important in the formation of the periodic surface structures at high laser fluence for which the metal melting and ablation depths are larger [120,121]. From an experimental point of view, Maragkaki et al. [109] produced the laser-induced periodic surface structures on a Cu (bulk target) polished surface at three different wavelengths (355, 532, 1064 nm) of a laser pulse with a duration of 7 ns. The formation and evolution of the surface structures were studied by a scanning electron microscopy analysis. As examples, Figure 29 and Figure 30 report the SEM images of the laser-induced periodic surface structures produced by a single laser pulse at different wavelengths (355 nm in Figure 29 and 1064 nm in Figure 30). From these images, the authors evaluated the experimental period Λ for the produced surface structures; the black dots in Figure 31.

According to Maragkaki et al., in the framework of the plasmonic model, the spatial period on the flat metallic surfaces is dependent on the laser wavelength λ by Λ = λ/[Re(√ε/1 + ε)] with ε being the wavelength-dependent dielectric constant of the metal [109]. Figure 31 reports the prediction of this equation by the full red line. The calculations based on a more advanced model, which considers the shape and filling factors to describe randomly rough surfaces, lead to the blue squares in Figure 31 (assuming reasonable values for the shape and filling factors). Figure 31 clearly shows the disagreement between the experimental data and the predictions of the models based on the surface plasmon polaritons, i.e., the surface plasmon polaritons cannot be the only reason for the formation of the laser-induced periodic surface structures. Maragkaki et al. [109], then, suggested a possible explanation for the laser-induced periodic surface structures’ formation based on a three-steps model involving hydrodynamic effects: (a) in the first step, the interference of the incident laser radiation with the surface scattered waves leads to a periodically modulated electron temperature in the metal; (b) in the second step, the evolution of the amplitude modulation in the electron temperature evolves occurs and, due to the electron-phonon thermalization, leads to an amplitude modulation in the lattice temperature [122]; (c) in the third step, the molten metal hydrodynamic processes act as a means to relocate materials along the surface, resulting in the laser-induced periodic surface structures’ pattern [112,113,114]. In particular, in this proposed model, metal redistribution induced by hydrodynamic instability should be dependent on many factors, including molten layer depth, melt viscosity and surface tension. These factors can provide the reasons for the deviation of the laser-induced periodic surface structures period from the initial interference pattern dictated on the surface by the interference of the incident and scattered laser waves.

Now, we return to the case of nanostructuration of the deposited thin metal films: Huynh et al. [56] performed similar experiments by Magkaki et al. [109], however, by irradiating Cu films deposited on glass and silicon substrates. In particular, in this case, the authors deposited 500 nm-thick Cu films on glass or Si substrates by magnetron sputtering and processed the films by a 42 ps-pulsed laser with a wavelength of 266 nm, a fluence ranging from 20 to 500 mJ/cm^2^ and the number of pulses ranging from 10 to 10000 and reaching the highly controlled production of periodic surface nano-patterns useful for several applications [123,124]. The authors studied the surface morphologies of the irradiated regions by scanning electron microscopy to understand the mechanism of the laser-induced periodic surface structures’ formation. Overall, the experimental results of Huynh et al. [56] for Cu films, allowed them to identify two distinct types of laser-induced periodic surface structures on the basis of the combination of laser fluence, the number of laser shots and the chemical nature of the substrate: (1) low spatial frequency laser-induced periodic surface structures (periodically spaced by Λ ≈ 260 nm and oriented perpendicularly to the laser polarization); (2) high spatial frequency laser-induced periodic surface structures (periodically spaced by Λ ≈ 130 nm and oriented parallelly to the laser polarization). In addition, a regime of spatially ordered spikes’ production was observed for a number of pulses higher than 1000. The model proposed by the authors for the spikes and laser-induced periodic surface structures’ formation is, notably, based on the theory of the Rayleigh instability [56].

**Figure 32:**
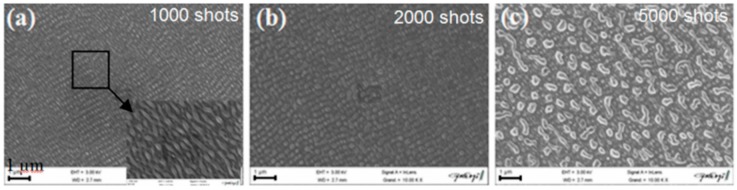
The formation of regular spikes on the Cu film surface (deposited on glass surface) after laser irradiations (42 ps-pulsed lasers with a wavelength of 266 nm and a fluence of 24 mJ/cm^2^) with (**a**) 1000, (**b**) 2000 and (**c**) 5000 shots. The SEM images are acquired at the center of the laser spot on the sample. Reproduced with permission from [56]. Copyright Elsevier, 2014.

**Figure 33:**
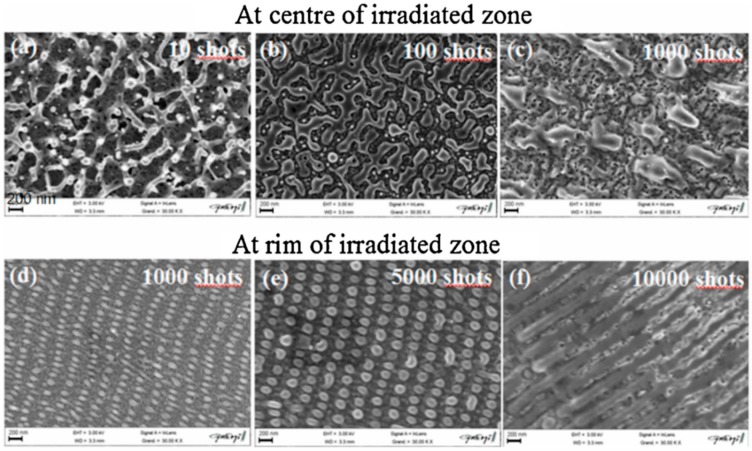
The spatial ordered array of spikes on the Cu film surface (deposited on glass) induced by the laser irradiations (42 ps-pulsed laser with wavelength of 266 nm and fluence of 199 mJ/cm^2^) with different numbers of laser pulses: (**a**) 10, (**b**) 100, (**c**) 1000, (**d**) 1000, (**e**) 5000, (**f**) 10000. In particular, the images in the first row are acquired at the center of the irradiated region, where the laser fluence is 199 mJ/cm^2^, while the images in the second row are acquired at the edge of the irradiated region where the laser energy is lower than 199 mJ/cm^2^. The images in the first row are different due to the increase of the number of the laser pulses (from 10 to 1000). The images in the second row are different due to the increase of the number of pulses which increases from 1000 to 10,000. Reproduced with permission from [56]. Copyright Elsevier, 2014.

As an example, Figure 32 reports SEM images showing the Cu surface morphology following the laser irradiation with a fluence of 24 mJ/cm^2^ (below the Cu film melting threshold) and increasing the pulse number from 1000 to 5000. As recognizable by these images, after 1000 pulses (Figure 32a), long spikes formed with a spatial period Λ ≈ 100 nm. Increasing the number of pulses to 2000, these long spikes evolved in circular spikes (by shrinking) with a diameter of about 200 nm on the grooves (Figure 32b). The distance between two neighboring grooves is about 270 nm. By increasing the number of pulses to 5000, the diameter of circular spikes increases to about 300 nm (Figure 32c). Figure 33 reports, furthermore, the SEM images showing the Cu surface morphology following the laser irradiation with a fluence of 199 mJ/cm^2^ (above the Cu film melting threshold) and increasing the pulses number from 10 to 10,000. In this case, the SEM images were acquired both in the center of the laser spot (first row in the figure) where the laser fluence is 199 mJ/cm^2^ and at the edge of the laser spot (second row in the figure) where the laser energy is lower than 199 mJ/cm^2^ due to the Gaussian shape of the laser intensity profile.

**Figure 34:**
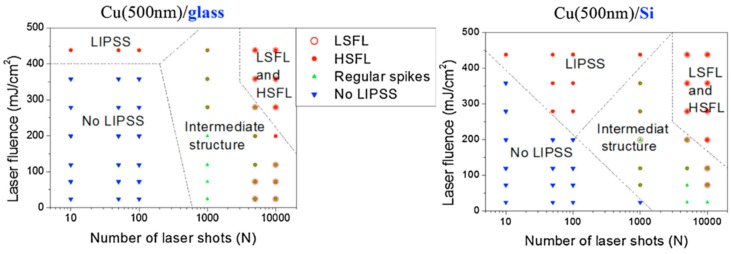
The diagrams summarizing the combined effect of number of laser pulses (N) and laser fluence in terms of the characteristic morphological structures obtained on the surface of the Cu film deposited on glass or silicon substrates processed at a wavelength of 266 nm and at a pulse duration of 42 ps. The acronyms are Laser-Induced Periodic Surface Structures (LIPSS), High Spatial Frequency LIPSS (HSFL), Low Spatial Frequency LIPSS (LSFL). Reproduced with permission from [56]. Copyright Elsevier, 2014.

Small spikes were formed at a laser fluence of 199 mJ/cm^2^ with the size increasing by increasing the number of laser pulses from 10 to 1000 (Figure 33a–c). For a number of pulses higher than 1000, the complete ablation of the Cu film from the supporting substrates was observed. On the other hand, at the edge of the irradiated region, for the number of the laser pulses in the 10–1000 range, no visible surface changes were observed due to the lower laser fluence. By increasing the number of pulses from 1000 to 10,000, the spatially ordered array of spikes and laser-induced periodic surface structures were produced with different shapes and periods (Figure 33d–f): after 1000 pulses, long spikes, characterized by a spatial period of about 110 nm on the grooves, are formed (Figure 33d). The distance between neighboring grooves is about 270 nm. At 5000 pulses, the long spikes evolve into circular spikes with a diameter of about 200 nm on the grooves (Figure 33e). After 10000 pulses, the formation of the low spatial frequency laser-induced periodic surface structures with a period of about 260 nm occur (Figure 33f). Then, by increasing the fluence to 438 mJ/cm^2^, the Cu thin films were ablated from the substrate at the center of the laser-processed just after less than 10 pulses. On the basis of the experimental results, Huynh et al. [56] constructed two-dimensional maps of the relationship among laser-induced periodic surface structures’ formation, laser fluence and number of laser shots on the Cu thin film on the two supporting substrates; see Figure 34. Concerning the Cu film on the glass substrate, the following key features can be identified: (a) for fluences lower than 400 mJ/cm^2^ and pulse numbers lower than 1000, the laser-induced periodic surface structures were not formed; (b) for fluences higher than 400 mJ/cm^2^ and the number of pulses lower than 1000, the laser-induced periodic surface structures were mainly grown; (c) for the number of laser pulses in the 1000–5000 range, an intermediate structure typology occurred, characterized by the combination of low spatial frequency laser-induced periodic surface structures, high spatial frequency laser-induced periodic surface structures and spatially ordered spikes; (d) at fluences higher than 200 mJ/cm^2^ and the number of pulses higher than 1000, a combination of high and low spatial frequency laser-induced periodic surface structures was formed. It is worth to note that the results obtained for the Cu film supported on the Si substrate are very similar to those obtained for the Cu film on the glass substrate, particularly at a high number of pulses, indicating that the substrate effect becomes practically negligible when the number of laser shots in increased, typically to over 2000.

As generally discussed in Section 2, the process of laser-metal interaction is, roughly, described by three steps: absorption of the laser energy by free electrons, absorbed energy redistribution (thermalization) through electron–photon coupling and, finally, ablation of materials by evaporation/sublimation or melt ejection. The thermalization time is dictated by the electron-phonon coupling and is typically in the range of 1–5 ps. The mechanisms of laser ablation are established by the laser pulse duration. Huynh et al. [56] used a 42 ps-pulsed laser so that the pulse duration is one order of magnitude greater than the thermalization time. Thus, the authors assume that the absorbed laser energy leads, primarily, to Cu melting. As a consequence, the formation of laser-induced periodic surface structures and spikes should be mainly dominated by capillary wave propagation mechanisms [125]. So, by increasing the temperature, the resulting gradient in the surface tension establishes a shear force determining the flow of the molten metal toward cold regions in order to minimize its energy and surface deformation. During the resolidification of the liquid film, the surface capillary waves (with the same periodicity of the intensity field in the molten metal) activates the relaxation of the deformation. These capillary waves lead to laser-induced periodic surface structures’ formation. More specifically, the laser-induced periodic surface structures’ formation can be attributed to the Rayleigh instability: this interpretation assumes that the ridges of laser-induced periodic surface structures are considered thin jets of liquid cylinders. This thin jet of liquid is more and more unstable to perturbations by increasing the number of laser pulses. So, the modification of the jet shape is driven by the reduction of the total surface energy. Then, the capillary force drives fluid away from the throat until it transforms into droplets. The models proposed by Maragkaki et al. [109] and Huynh et al. [56] to justify the formation of the surface periodic structures are based on hydrodynamic (Rayleigh) instabilities. However, it is clear [79,80,81,82,83] that even if this could be an important phenomenon driving the formation of the periodic surface structures, it alone cannot completely explain the formation mechanism. In fact, if the formation mechanism would be dominated solely by the hydrodynamic instability, the finally obtained patterns should not be spatially oriented according to the laser polarization (once thermalized, electrons have lost any memory of the incident laser polarization), contrary to the general obtained experimental results [79,80,81,82,83]. It is clear, then, that the formation of the laser-induced periodic surface structures is driven by a combination of hydrodynamic instabilities and, at least surface-scattered electromagnetic waves [83].

From a technological point of view, we mention that laser-induced periodic surface structures attract technological interest since they show water-repellent properties, diffusive reflection, and friction change.

To complete this section, we mention that the interference of picosecond-pulsed lasers can be exploited for the direct controlled structuration of metal films. In this framework, for example, Voisiat et al. [55] reported results of the laser beam interference ablation of thin metal films (Al, Ag, Au, Cu, Cr with thickness in the 100–500 5 00 nm range) on glass using a 1064 nm-wavelength, 60ps-pulsed laser.

**Figure 35:**
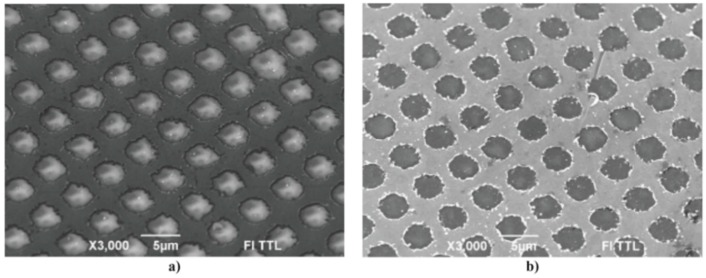
The SEM images of interference patterns produced in Ag film (100 nm-thick) on glass with a single laser pulse (**a**) and Au film (100 nm-thick) with 3 laser pulses (**b**) using four interfering beams without a phase difference (wavelength of 1064 nm, energy = 0.7 mJ, period of the holes = 5 μm). Reproduced with permission from [55]. Copyright Elsevier, 2011.

The laser beam was divided into four beams by diffractive means and, then, an imaging system was used to focus the beams on the same area on the metal film surface so as to produce the interference pattern on the metal film surface. By controlling the process parameters (beams phase difference, laser energy), several classes of periodical patterns were formed; see Figure 35 for a specific example. These experiments clearly show that laser patterning using interference of several beams is a powerful approach in producing sub-wavelength features on metal surfaces arranged in two-dimensional spatial-organized arrays. The periodical structure can be controlled by changing, for example, the incidence angle of the beams, the wavelength, the phase difference between the beams, the polarization and energy of the laser. One-dimensional and three-dimensional arrays can also be produced by interfering several laser beams [126,127,128,129]. In the example reported in Figure 35, images of the ablation patterns obtained by Voisiat et al. [55] in Ag film (100 nm-thick) on glass with a single laser pulse (Figure 35a) and Au film (100 nm-thick) with 3 laser pulses (Figure 35b) using four interfering beams without a phase difference (wavelength of 1064 nm, energy = 0.7 mJ, period of the holes = 5 μm). Generally, the resulting shape of the structures obtained by the metal ablation is dependent on the phase difference between the interfering laser beams pairs and on the laser fluence. In fact, the metal is removed from a substrate in the regions where the local laser fluence overcomes the threshold value for ablation.

## 5. Nanostructuration of Thin Metal Films by Femtosecond Pulsed Laser Irradiation

**Figure 36:**
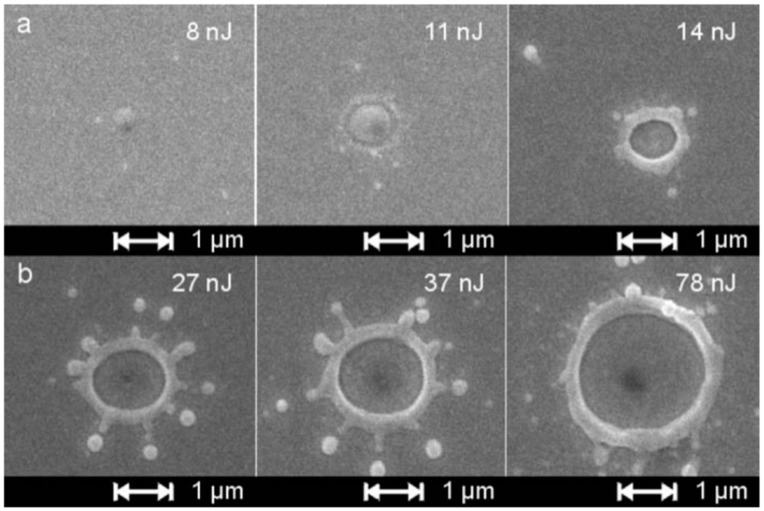
The SEM images showing the results of the single-pulse laser ablation (laser of wavelength 800 nm, pulse duration of 30 fs) of a 100 nm-thick Cr film on glass substrate, increasing the laser energy. The sequences in (**a**) and (**b**) are separated by a critical value for the laser energy and above this value Cr droplets ejection is observed. Reproduced with permission from [57]. Copyright Springer, 2003.

In the last years, several detailed investigations on the exploitation of femtosecond-pulsed lasers for nanostructuring metallic films on substrates were reported, in particular towards the fabrication of two- and three-dimensional nanostructures with a tuned shape, size, and spatial order [57,58,59,60,61,62,63,64,65,66,67]. In 2003, Korte et al. [57] reported on the use of femtosecond lasers for nanofabrication of metal structures by processing deposited metal films with a characteristic size on the order of several hundred nanometers (100–500 nm); see Figure 36 as an example.

Generally, when femtosecond laser radiation impinges onto a solid material, the photo- and thermal emission of electrons from the material can occur [57]. Whereas photoelectric emission is the dominant electron emission effect at low electron temperatures, electron thermionic emission is the dominant one at high electron temperatures. Theoretical calculations and experimental results show that for metals processed by a 100 fs-pulsed laser, the threshold fluence of 1mJ/cm^2^ establishes the transition from multiphoton to thermionic emission [57,130,131]. This last process takes place due to the extension of the high-energy tail of the electron distribution above the vacuum level. In this condition, the fs-pulsed laser irradiation of metal allows a situation in which the electron system temperature is much higher than the phonon system temperature, resulting in a transient decoupling of the electron and lattice subsystems. Thus, the femtosecond-pulsed laser irradiations of metals provides the possibility to transfer the laser energy directly into the electron subsystem, leaving the lattice thermally unaltered. Another important fact characterizes the femtosecond-pulsed irradiation of metals [57]: usually, a diffusive behaviour characterizes the electron heat distribution so that a heat penetration depth can be defined, which has a square root dependence on time. However, when the metal is irradiated by a fs-pulsed laser, a strong electron–lattice non-equilibrium condition is realized, resulting in a linear dependence (or even more complicated) of the heat penetration depth with time [132,133].

**Figure 37:**
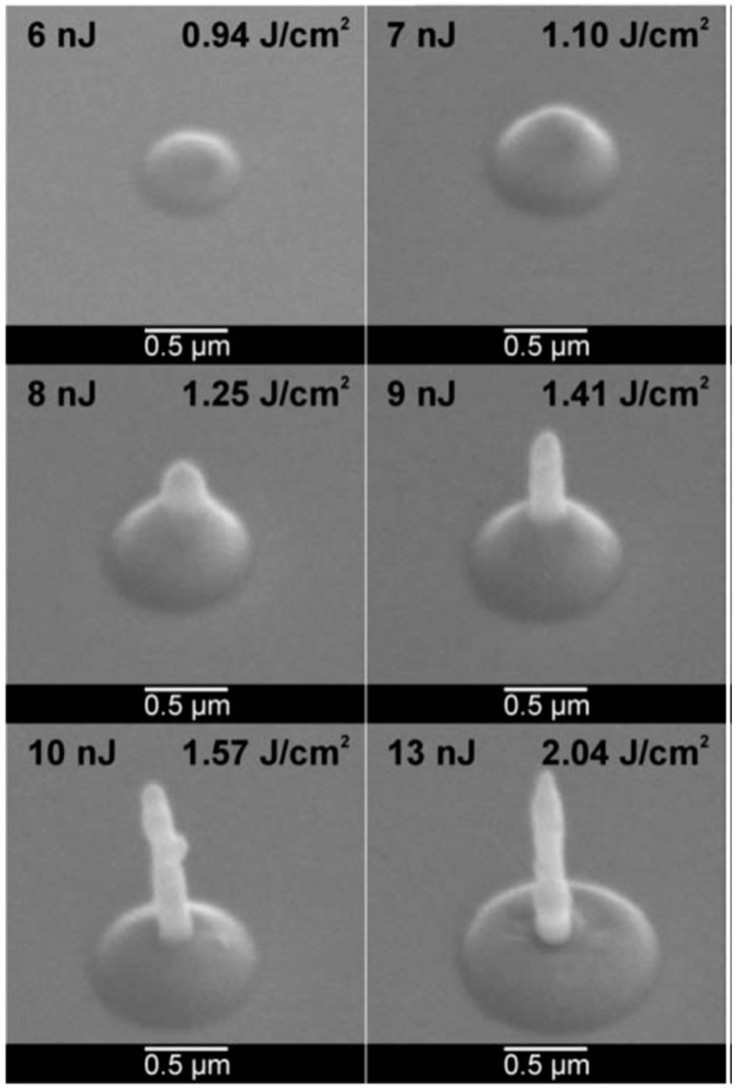
The SEM images showing the results of the single-pulse laser ablation (laser of wavelength 800 nm, pulse duration of 30 fs) of a 60 nm-thick Cr film on quartz glass substrate, increasing the laser energy. Reproduced with permission from [58]. Copyright Springer, 2004.

**Figure 38:**
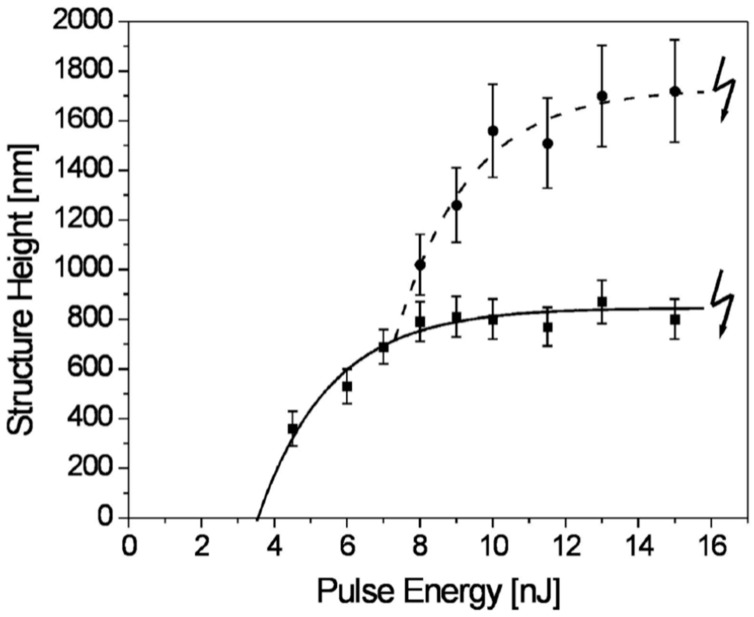
The height of the Au microbumps (solid curve) and nanojet (dashed curve) versus the laser pulse energy (30 fs pulsed laser with 800 nm wavelength). The broken arrows indicate the fact that by starting from 16 nJ of laser energy, the Au modification process evolves to an unstable condition resulting in the destruction of the microbubbles and the nanojets. Reprinted with permission from Reference [58], Springer, 2004 Reproduced with permission from [58]. Copyright Springer, 2004.

**Figure 39:**
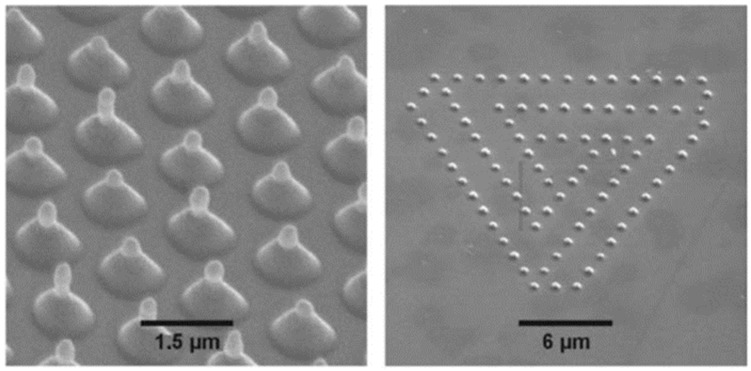
The SEM images showing two cases of spatially organized structures fabricated in a 60 nm-thick Au film on quartz by femtosecond laser pulses (30 fs pulsed laser with an 800 nm wavelength). Reproduced with permission from [58]. Copyright Springer, 2004.

From an experimental point of view, Korte et al. [57] analyzed, first of all, the possibility to fabricate sub-wavelength structures in metal films deposited on substrates. As an example, Figure 36 reports SEM images of surface structures, produced by single laser pulse, with different pulse energies, on a 100 nm-thick Cr layer deposited on a glass substrate (laser wavelength of 800 nm, pulse duration of 30 fs). On the basis of the laser energy, three different structuring regimes can be identified [57]: at low laser energies (first regime, Figure 36a), sub-micrometric morphological modifications of the Cr film are obtained. In this regime, the metal ablation process occurs in combination with the formation of a small bubble. At higher laser energies (second regime, Figure 36b), a hole is obtained with a sub-wavelength-sized ring and multiple droplets around. By further increasing the laser energy, the size of the hole increases. For laser fluence equal or higher than the substrate ablation threshold (third regime), sub-micron structuring of the substrate can occur. Such an evolution is typical for transition metals (Cr, Mo, W, Fe) and is determined by the electron–phonon relaxation. For these materials, the transfer of energy to the lattice proceeds much faster than for noble metals [134]: as an example, the electron-phonon coupling constant is G_ep_ ~ 42 × 10^16^ W m^−3^ K^−1^ for Cr and G_ep_ ~ 2.3 × 10^16^ W m^−3^ K^−1^ for Au [47,60,86]. Thus, as can be recognized in Figure 36, by using a fs-pulsed laser on metallic films, the surface features with typical size overcoming the diffraction limit can be produced by fixing the laser fluence slightly above the metal film ablation threshold. While in transition metals (Cr, Mo, W, Fe) the structuring mechanism under fs-pulsed laser is due to the direct laser ablation (due to the rapid electron–phonon relaxation, which is responsible for the fast energy transfer to the lattice and material removal), in noble metals (Au, Ag) under an fs-pulsed laser, the structuring mechanism is due to an ablation process affected by metal flow dynamics in the molten phase due to a much slower electron–phonon relaxation (i.e., slower energy transfer from the electron system to the lattice). In fact, in noble metals such as Au and Ag, the lattice melts and the molten phase exists much longer. This effect changes the ablation dynamics. In this regard, for example, further studies by Korte et al. [58] demonstrated the possibility to produce microbumps and nanojets on Au films deposited on the quartz substrate and processed by femtosecond laser pulses (Figure 37). As an example, Figure 37 presents SEM images of the resulting modification of a 60 nm-thick Au film on the quartz substrate processed by a single pulse of a 30 fs-pulsed laser with a wavelength of 800 nm and increasing the laser energy. The sizes of the microbumps and nanojets depend on the laser pulse energy. By tuning the laser energy, Au jets with a planar size of 100 nm and a height higher than 1 μm can be fabricated; see Figure 37. Nanojets always appear on a bump-like structure. Sharp laser energy thresholds characterize the formation of the micro-bumps and of the nanojets [58]. For the case of Au (Figure 37), first, the surface bump rises for a laser fluence higher than 0.5 J/cm^2^ and then the nanojets appear for a fluence higher than 1.1 J/cm^2^. Figure 38, according to Korte et al. [48], reports on the height of the bump and nanojet structures versus the laser energy. In the figure, the broken arrows indicate the fact that by starting from 16 nJ of laser energy, the Au modification process evolves to an unstable condition resulting in the destruction of the microbubbles and the nanojets. It is worth to observe in Figure 39 that this technology also provides the possibility to produce a large-area patterning of Au to form large-area periodic arrays of microbumps and nanojets on the Au surface.

Korte et al. [58] discuss the formation of the microbumps and nanojets, observing that their morphology suggests a process similar to that induced by a droplet fall into a glass filled with liquid. In this last case, the waves originated on the surface of the liquid by the impinging drop collide at the center of the drop-liquid collision region and form a liquid jet. Similarly, the Au film is molten by the femtosecond laser pulse and the waves produced on the surface of the molten Au layer collide at the center of the molten area, producing a molten jet which then becomes solid due to the fast solidification process. On the surface of the Au molten layer, strong radial temperature gradients develop so that the Marangoni effect could be likely to occur [58,135,136], i.e., the radial convective flow of the molten metal could take place due to the developed radial surface tension gradients established by the temperature gradients. In the particular case of Au, its surface energy is a decreasing function of temperature so that the molten Au convection motion should occur from the center to the edge of the irradiated area. However, during the laser pulse, the electron temperature is still higher than the Au melting temperature and the continued energy transfer from the electrons to the lattice can establish an inverted condition for which the surface of the molten layer has a lower temperature than the bottom. In this inverted condition, the combined effect of the molten Au convection flow and the continuing energy transfer from electrons to the lattice can initiate the Au vaporization process from the bottom of the molten layer, leading to the formation of the hollow bump in the first row in Figure 37). By further increasing the laser energy, the nanojet structure (i.e., a splash structure) at the center of the irradiated area originates from the collision of the energetic molten Au flows at the center. Then, when the lattice temperature decreases, the solidification of the molten Au starts from the edge of the previously molten region and proceeds towards the center (see second and third row in Figure 37). Such a picture, in some respects, is consistent with the theoretical results by Ivanov and Zhigilei [87]: they computationally analyzed the kinetics and microscopic processes involved in the laser melting and disintegration of thin free-standing Au film (allowing film dilatation in two directions) under a fast laser pulse.

The calculations indicated the existence of strong pressure variations in the molten Au causing the formation of a bubble inside the Au film. The case of Au on a substrate, as experimentally investigated by Koch et al. [59], is different for the presence of the supporting glass substrate which suppresses dilatation in one direction and possibly boosts the film dilatation perpendicular outwards from the free Au surface. During the bubble expansion, in the local region of the bump with the highest temperature and higher expansion rate, the nanojet structure develops and the nanojet increases its height after every bubble pressure increase [59]. However, the exact mechanism for the microbumps and nanojets formation in the metallic films processed by fs-pulsed laser irradiation are, yet, the matter of studies and discussions.

**Figure 40:**
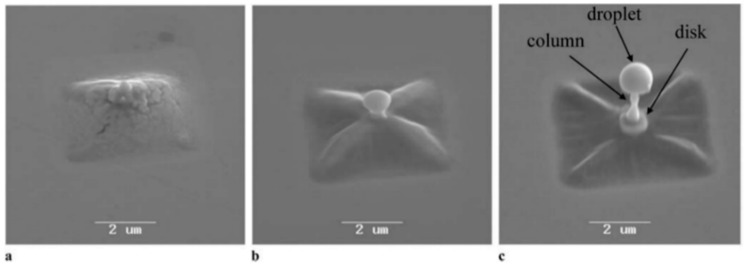
The structures obtained by the irradiation of a 60 nm-thick Au film (on glass substrate) with a single laser pulse (wavelength 800 nm, pulse duration 30 fs) having a square-shaped intensity distribution. The laser fluences are 0.190 J/cm^2^ (**a**), 0.195 J/cm^2^ (**b**), and 0.2 J/cm^2^ (**c**). Reproduced with permission from [63]. Copyright Springer, 2009.

Overall, the main models proposed in the literature for the description of the bubbles and nanojets formation can be summarized as follows: thermoplastic deformation of the solid (non-molten) film [60], Marangoni convection flow of the melted film [58], evaporation of material under the film [61], and photochemical spallation or relaxation of the compressive stresses generated by the fast laser heating and melting of the film [62,65,87]. In this sense, to better clarify the mechanism, further experimental studies by Kuznetsov et al. [63] regarded the femtosecond-pulsed laser (wavelength 800 nm, pulse duration 30 fs) modification of 60 nm-thick Au film on glass substrates using pulses characterized by different microintensity distributions so as to obtain a wide range of microstructures with controlled nano-shapes. As an example, Figure 40 shows SEM images of the structures fabricated on the Au film surface by a single laser pulse with a square-shaped intensity distribution; at 0.190 J/cm^2^ fluence (Figure 40a), a pyramidal shape bump is produced on the Au surface; at 0.195 J/cm^2^ fluence (Figure 40b), the Au concentrates in the central squared area, forming a cross with a jet in the middle having a particle on the top. The height of the jet and the size of this particle increase by increasing the fluence to 0.200 J/cm^2^ (Figure 40c), while the film becomes thinner at the peripheries of the irradiated region. The presence of the particle on the top of the jet is consistent with a process occurring for Au in the molten state.

**Figure 41:**
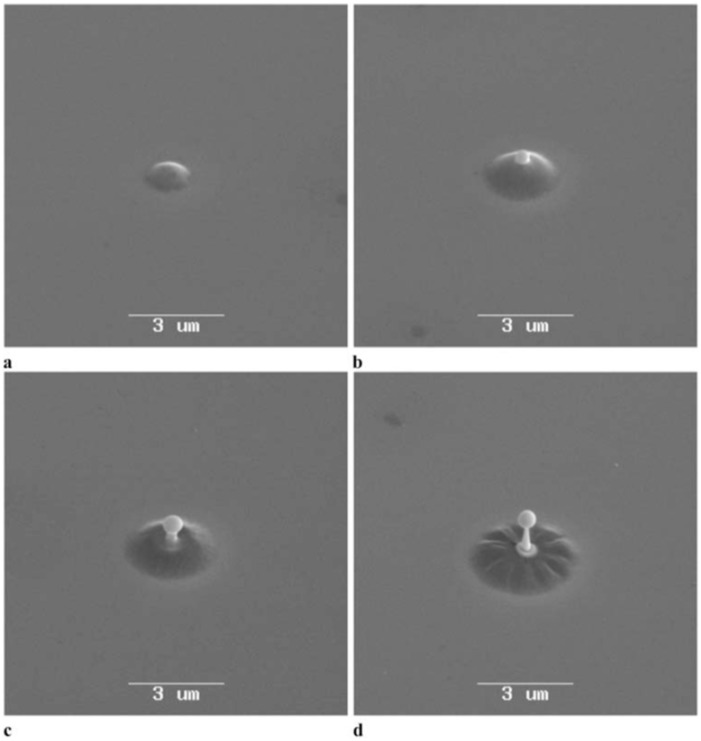
The structures obtained by irradiation of a 60 nm-thick Au film (on glass) with a single laser pulse (wavelength 800 nm, pulse duration 30 fs) with an energy of 40 nJ (**a**), 46 nJ (**b**), 58 nJ (**c**), 78 nJ (**d**). In this case, an achromatic lens was used to focus the gaussian laser beam, having a diameter of 8 mm, on the Au film surface. Reproduced with permission from [63]. Copyright Springer, 2009.

**Figure 42:**
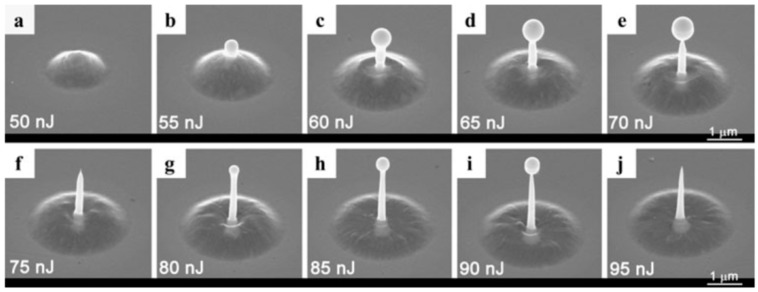
The structures obtained by irradiation of a 60 nm-thick Au film (on glass) with a single laser pulse (wavelength 800 nm, pulse duration 30 fs). In this case, the gaussian laser beam with the diameter of 8 mm has been focused on the sample surface with a 20 mm achromatic lens. The laser pulse energies are indicated in the images and increases from (**a**) to (**j**). Reproduced with permission from [66]. Copyright Springer, 2012.

An important feature which can be clearly recognized is the redistribution of the material from the edges to the center of the irradiated area, a process which was only roughly observed in earlier experiments by Korte et al. [58] and Koch et al. [59], with a focus on Gaussian laser pulses. Kuznetsov et al. [63] obtained similar results for Au performing irradiations with a gaussian shape beam having a large focus diameter [63]; see Figure 41. It can be recognized in Figure 41d that, in this case, the redistribution of Au from the edges to the center of the irradiated area also occurs at higher fluences and that the jet structures in Figure 40c and Figure 41d present three similar key features: the particle on the top, the column in the middle, the ring at the bottom. The redistribution of the Au from the edges to the center of the irradiated region is most likely due to the melted material dynamics. A strong indication for the process occurring in the metal molten state is the presence of the droplet on the top of the jet. In the same conditions (60 nm-thick Au film on glass substrate, laser wavelength = 80 nm, pulse duration = 30 fs), Kuznetsov et al. [66] further extended the experimental work by increasing the laser energy—see Figure 42—using a laser beam having gaussian shape intensity. At the laser energy of 50 nJ, the Au microbump is obtained (Figure 42a). By slightly increasing the energy (55 nJ), the nanojet structure appears at the center of the microbump (Figure 42b). The height of this jet increases with the energy and a particle is formed on the top of the jet (Figure 42c–e). When the laser energy is higher than the threshold value of 75 nJ, the particle is detached from the Au film surface (Figure 42f). Further increasing the laser pulse energy leads to the formation of a second particle on the top of the same jet and its detachment from the Au film surface at the energy threshold of 95 nJ occurs (Figure 42g–j). The lateral size of the bump increases with the laser energy and its walls become thinner, revealing the jet structure underneath the bump. These experimental analyses by Kuznetsov et al. [66] support the mechanism for the bumps’ formation which occurs during the metal molten phase followed by the metal solidification and this is in agreement with molecular dynamic simulations by Ivanov et al. [62,65,87]. According to these simulative models, the molten Au is detached from the substrate surface and accelerated in the direction perpendicular to the substrate surface. These simulations show that the laser irradiation induces the heating and melting processes of the metal films and the following metal thermal expansion gives origin to the mechanical stresses in the metal film and the consequent relaxation of the developed compressive stresses, finally, determining the molten Au perpendicular detachment from the substrate. In fact, the density of molten Au is lower than that of the solid Au [137] so that the melting results in the Au expansion.

**Figure 43:**
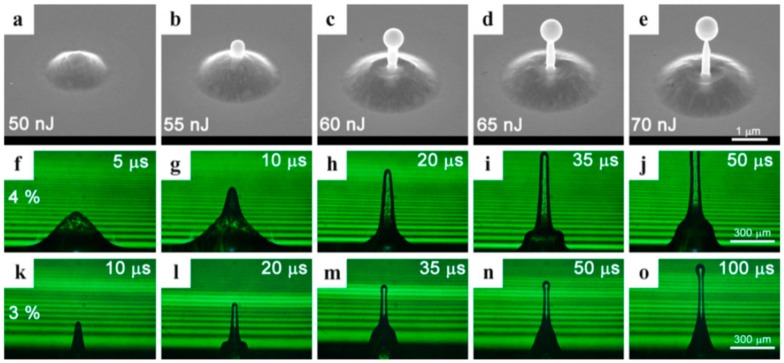
(**a**–**e**) The tilted SEM images of structures produced on a 60 nm-thick Au film surface by a single 30 fs-pulsed laser pulse. (**f**–**o**) Time-resolved images of liquid jets formed on a surface of two different liquids after irradiation by a 9 ns-pulsed laser pulse with a 40 μm focus diameter. Images (**f**–**j**) correspond to a laser pulse energy of 21 μJ and a 4% alginate solution. Images (**k**–**o**) correspond to a laser pulse energy of 14 μJ and a 3% alginate solution. Reproduced with permission from [66]. Copyright Springer, 2012.

**Figure 44:**
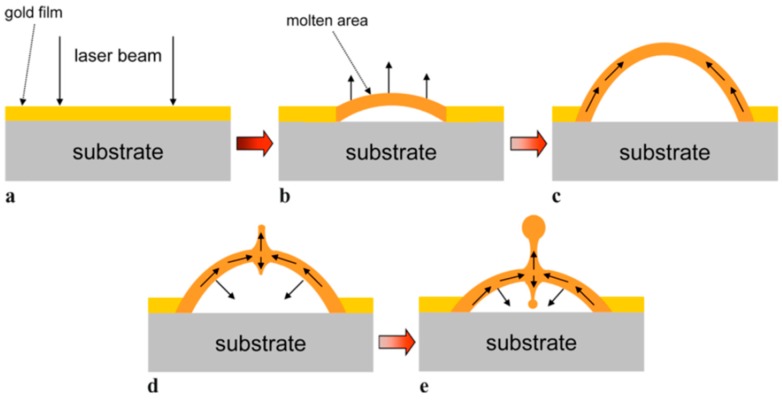
The sequence of pictures illustrating the mechanisms responsible for femtosecond laser-induced formation of structures on a thin Au film on a substrate: the figures from (**a**) to (**e**) picture the temporal steps involed in the overall formation process. Reproduced with permission from [66]. Copyright Springer, 2012.

During the rapid solidification of the molten Au, the film detaches from the substrate, creating the bump structure. The increase of the laser energy leads to the hydrodynamic flow of molten Au as established by the surface tension, which causes the evolution of the structure toward the jet protrusion formation on the top of the bump. Kuznetsov et al. [66], in addition, reported a comparison of the images of structures formed on a thin Au film surface by a single femtosecond laser pulse and jets generated on a liquid film surface by a single 9 ns-pulsed laser; see Figure 43 (liquid jets are imaged using a time-resolved imaging technique). As can be recognized in Figure 43, the jets formed in these two cases are morphologically very similar. However, obviously, in the case of the laser irradiation of thin Au films, the structures are obtained after solidification of the molten film irradiated at different laser energies, while in the case of the liquid jets, the images refer to different delay times after the laser pulse. In the case of the irradiation of thin Au films, increasing the laser pulse energy leads to a delayed solidification of the molten Au. The following features in the jet formation are evidenced by Figure 43: (1) first, the formation of a bubble and its growth in size (Figure 43a,f,k), (2) the appearance of a jet on top of the bubble (Figure 43b,g,l) and (3) the jet growth and appearance of a particle (drop) at the jet top (Figure 43c–e,m–o). Kuznetsov et al. [63,66] proposed the following picture to explain the nanobump and microjet formation (see Figure 44): firstly, the metal film is completely melted by the laser radiation in the focal area and detaches from the supporting substrate due to the relaxation of compressive stresses (Figure 44a,b). The behaviour of this melted area is established by the gradients in the surface tension caused by the inhomogeneous laser-induced material temperature distribution. For pure liquid Au, the thermal coefficient ∂γ/∂T (γ the liquid surface tension) is negative and it is the driving force for the Au redistribution from the hot to the cold areas. The movement of the molten Au film upwards creates a zero-pressure bubble underneath but, at the same time, the surface tension decelerates the bubble and induces its collapse. This deceleration is accompanied by the inertial movement of the molten material towards the apex of the bump and the formation of two liquid jets directed in opposite directions: inside and outside the bump (Figure 44c,d). Further, the droplets are formed on the top of these liquid jets due to the strong surface-tension forces (Figure 44e). Finally, since the formation of a jet and several particles under laser irradiation of Au films is governed by hydrodynamic processes inside the laser-molten area, it is reasonable to think that long liquid jets are unstable and can decay into multiple droplets due to the Rayleigh instability.

The formation of similar structures with a similar morphology evolution (and in similar conditions of irradiation) was observed for other metals such as Ag and Cu [66]. The general requirements for the occurring of the processes leading to the formation of the bumps and jets can be summarized as follows: (a) the weak adhesion of the metal film to the substrate allowing the formation of the bump and, then, its collapse (for example, the formation of these structures was not observed for Ti films which better adhere on glass than Au, Ag, Cu [66]); (b) a higher density for the metal in the molten phase than in the solid one, allowing the development of strong compressive stresses inside the film in the melting process which is the fundamental condition for bump formation; (c) a high viscosity of the molten metal since, if the viscosity is not sufficiently high, the bubble is not formed and breaks at the initial step of its formation [66].

**Figure 45:**
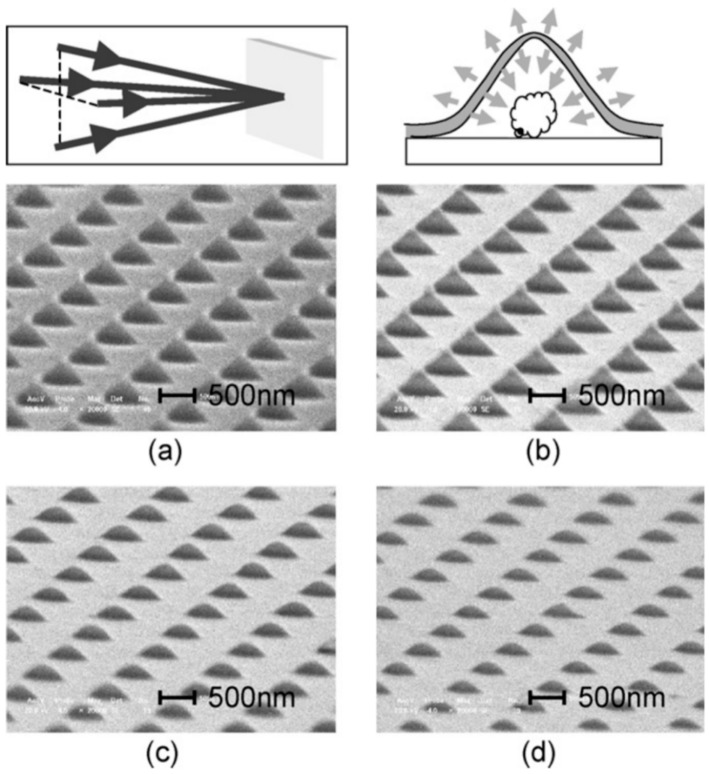
The sequence of SEM images elucidating the formation and evolution of the nanobump array generated on the Au thin film by four interfering femtosecond laser beams. The laser fluence is 87 mJ/cm^2^ with a wavelength of 780 nm, and a pulse duration of 120 fs (**a**), 355 fs (**b**), 741 fs (**c**), 1220 fs (**d**). The top left inset illustrates the beam incidence on the film and top right inset illustrates the formation process of the bump. Reproduced with permission from [61]. Copyright Elsevier, 2007.

**Figure 46:**
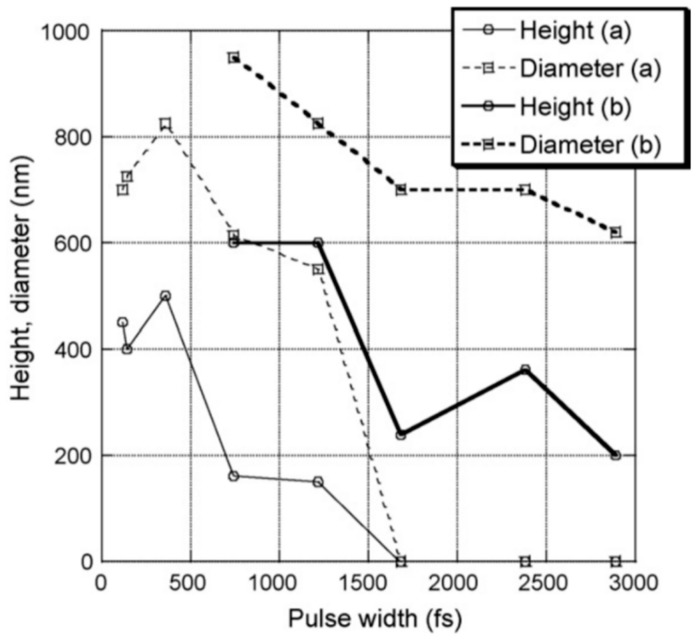
The evolution (as a function of the laser pulse duration) of the height and diameter of the nanobump as a function of the pulse duration at (**a**) 87 mJ/cm^2^ and (**b**) 114 mJ/cm^2^ (laser wavelength of 780 nm). Reproduced with permission from [61]. Copyright Elsevier, 2007.

**Figure 47:**
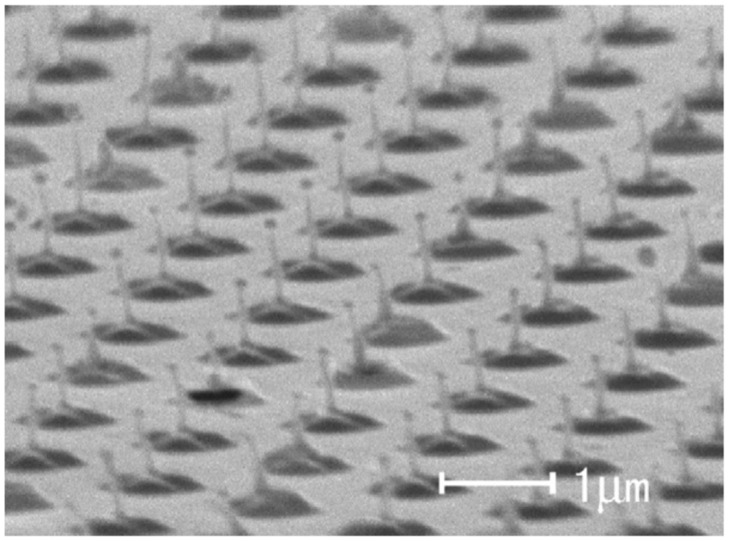
The nanojets array generated at the pulse duration of 2.4 ps and at a fluence of 190 mJ/cm^2^. Reproduced with permission from [61]. Copyright Elsevier, 2007.

Exploiting the above-described approach reviewed, Nakata et al. [61] demonstrated the possibility to generate spatially ordered arrays of conical Au nanobumps by processing thin deposited Au films with an interfering femtosecond laser; see Figure 45. In this case, the authors deposited a 50 nm-thick Au film on quartz glass substrate and processed the film by interfering four laser beams by changing the laser energy and the pulse duration (fixing the laser wavelength of 780 nm). SEM images of the obtained nanobump arrays using a laser fluence of 87 mJ/cm^2^ and increasing the pulse duration are reported in Figure 45. In this case, when the pulse duration is 120 fs or 355 fs, the shape of the structures is conical and the nanobumps almost have the same size. On the other hand, when the pulse duration is 741 fs, the nanobumps’ size is smaller and even smaller for a pulse duration of 1220 fs. Furthermore, the evolution of the height and diameter of the nanobumps, as a function of the pulse duration for the laser fluence of 87 mJ/cm^2^ and of 114 mJ/cm^2^, is reported in Figure 46. As can be recognized from this plot, when the laser fluence is 87 mJ/cm^2^, there are small changes under 350 fs, but they decreased over this pulse duration and no nanobumps are formed when the pulse duration is greater than 1.6 ps. On the other hand, when the fluence is 114 mJ/cm^2^, the film ablates and the nanohole array is generated under 700 fs. The size of the nanobumps decreases by increasing the pulse duration, as is the case at 87 mJ/cm^2^. The shape of a nanojet in the array generated at a longer pulse duration (2.4 ps) and a higher fluence (190 mJ/cm^2^) remembers the typical liquid-like morphology, as shown in Figure 47. In fact, the shape of the nanojets strongly recalls the stop-motion shape of a liquid drop, as already recognized by Kuznetsov et al. [63,66]. As discussed by Nakata et al. [61] for these experiments, electrons of the metal are firstly excited by the femtosecond laser irradiation and thermalized in about 100 fs. Then they diffuse by raising the temperature of the material in some ps. When the fluence is 87 mJ/cm^2^, the size of nanobump decreases at pulse durations longer than 350 fs, which is due to the energy loss due to heat radiation or due to the diffusion of high energy electrons from an excited region to a not-excited region, or through the heat conduction to the substrate.

The loss of energy can be compensated by using higher fluence. For example, the size of the nanobump at 87 mJ/cm^2^ and at 750 fs is almost the same at 114 mJ/cm^2^ and at 2.9 ps. At the long pulse width of 2.4 ps and the high fluence of 190 mJ/cm^2^, they had a liquid-like structure, as shown in Figure 47.

**Figure 48:**
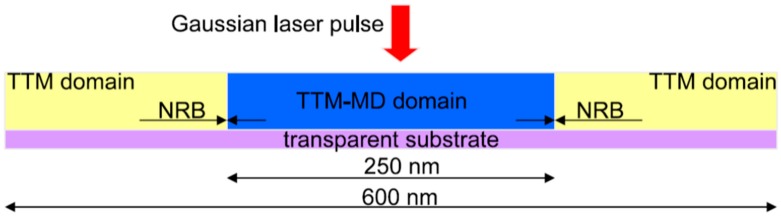
The schematic picture of the computational setup used in molecular dynamics–two temperatures model calculations employed by Ivanov et al. Acronyms: Molecular Dynamics (MD), Two Temperatures Model (TTM), Non-Reflective Boundary (NRB). Reproduced with permission from [62]. Copyright Springer, 2008.

**Figure 49:**
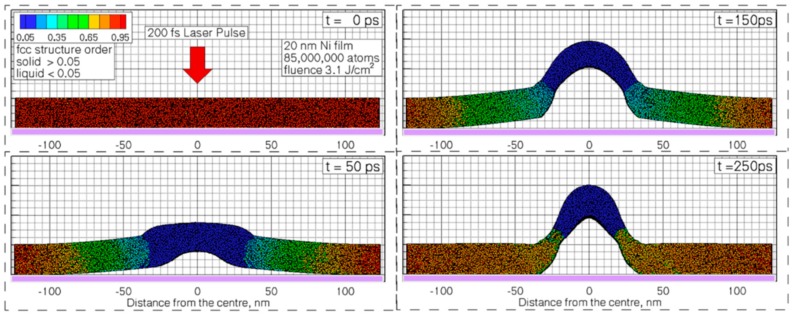
Snapshots from a molecular dynamics-two temperatures model simulation of a 20 nm-thick Ni film deposited onto a transparent substrate and processed by a 200-fs laser pulse focused on a 10 nm spot in the middle of the computational cell. The average fluence absorbed within the beam diameter is 3.1 J/cm^2^. Atoms are colored according to the local order parameter so that red atoms have local crystalline surroundings, blue atoms belong to the liquid and, in the last snapshot, to small crystallites disoriented with respect to the original crystalline structure of the film. Reproduced with permission from [62]. Copyright Springer, 2008.

**Figure 50:**
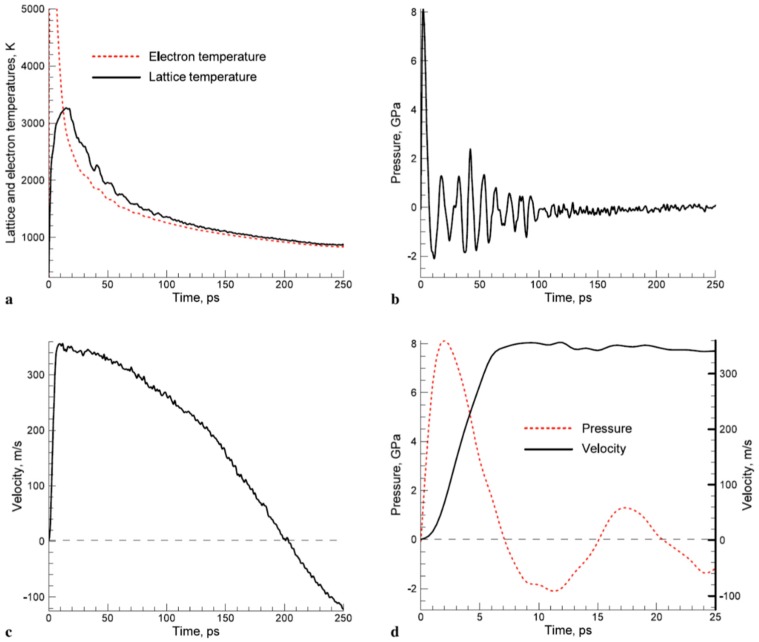
The calculated time evolution of the electron and lattice temperatures (**a**), pressure (**b**), and velocity in the direction normal to the substrate (**c**) averaged over a part of the film within 2 nm from the center of the laser spot. The starting changes of pressure and velocity during the first 25 ps of the simulation are shown with a higher resolution in (**d**). Reproduced with permission from [62]. Copyright Springer, 2008.

Ivanov et al. [62,65,87] extensively studied, by employing several simulation-based approaches, the mechanisms involved in the formation of the nanobumps and nanojets in metal films under femtosecond-pulsed laser irradiations. In particular [62], by employing large-scale molecular dynamics calculations combined with an atomistic-continuum model, they identified the relaxation of the compressive stresses generated by fast laser heating as the main driving force for the separation of the metal film from the substrate and the formation of the nanobump. They argued that the kinetics of the metal transient melting and resolidification occurring under conditions of fast cooling due to the two-dimensional electron heat conduction establishes the shape of the nanobump. According to Ivanov et al. [62], Figure 48 shows a schematic picture of the computational cell used for the simulation: the simulations are performed for a 20 nm-thick Ni film deposited on a transparent substrate and the initial molecular dynamic part of the model is a circular slab that is 250 nm in diameter, where atoms are arranged in the FCC crystal structure with a (001) surface oriented parallel to the substrate. The thermal and elastic properties of the Ni film are defined by the interatomic interaction potential (establishing, also, an adhesion energy of the Ni film to the substrate 10 times weaker than the Ni–Ni cohesive energy, as a typical situation for metal films on non-metal substrates). The molecular dynamic model simulates the non-equilibrium processes of the lattice heating and fast phase transformations with a continuum description of the laser excitation and subsequent relaxation of electrons based on the two-temperatures model. The substrate is kept rigid during the simulation and the energy exchange between the Ni film and the substrate is not allowed. The two-temperature model equations for the electron and lattice temperatures are solved up to more than 300 nm from the center in the radial direction; see Figure 48. A non-reflective boundary condition—see Figure 48—is applied circumferentially around the molecular dynamic computational cell, which describes the propagation of the laser-induced radial pressure wave from the molecular dynamic region to the continuum part of the model. The choice of Ni rather than Au was dictated by computational necessity: the stronger (~one order of magnitude) electron–phonon coupling of Ni and the negligible contribution of the ballistic energy transport resulted in a fast lattice heating and a high degree of laser energy localization near the laser spot. The simulations concern the effect of a laser pulse with a 200-fs pulse duration and a fluence of 3.1 J/cm^2^ focused on the Ni film over a spot 10 nm in size. The visual pictures of the time evolution of the Ni film after the laser pulse are summarized in Figure 49: the atoms in the images are colored according to the local order parameter, with blue color indicating the destruction of the original FCC crystalline order due to the fast melting. The Ni melting starts after 2–3 ps from the laser pulse and the radius of the melted region reaches its maximum size (about 32 nm) after 20 ps. The Ni melting process proceeds simultaneously with the detachment of the central part of the film from the substrate and the generation of a pronounced hollow nanobump due to the rapid bloating of the melted region away from the substrate. The physical processes leading to the nanobump formation can be argued by considering the results of the calculations for the time evolution of the electron and lattice temperatures, pressure, and velocity in the direction normal to the substrate, as reported in Figure 50. Due to the small heat capacity of the electrons, the laser excitation causes a large increase of the electron temperature that reaches about 16,000 K at the end of the pulse (Figure 50a).

The sharp drop of the electron temperature during the first 10 ps of the simulation is due the fast energy transfer from the electrons to the lattice. The fast energy transfer from the electrons to the lattice is reflected in the initial increase of the lattice temperature, which exceeds the melting temperature for Ni (1439 K) and reaches the maximum value of about 3250 K after 15 ps. At longer times, the cooling stage starts due to the electron heat conduction and energy transfer from the lattice to the electrons. The fast-localized heating of the film occurs under conditions of the partial inertial stress confinement [138] when the heating time is shorter than the time needed for the film to expand in response to the thermoelastic stresses. The results of the simulations for the compressive stress developed in the film are shown in Figure 50b,d, indicating the raising of the compressive stress up to 8 GPa during the first several picoseconds of the simulation. The relaxation of the compressive stresses proceeds by expansion in both the radial and normal directions to the substrate. On the rigid substrate, the normal expansion of the Ni film results in the upward acceleration of the film up to a velocity higher than 300 m/s; Figure 50c. The expansion of the film may be treated as an unloading (tensile) wave propagating from the free surface toward the substrate, resulting in the concentration of the tensile stresses at the film-substrate interface and leading to the separation of the film from the substrate. The correlation between the time of the film acceleration and the first peak of the compressive pressure—Figure 50d—indicates that the relaxation of the laser-induced compressive stresses is the reason for the acceleration of the central part of the film. Strong electron temperature gradients established in the radial direction following the initial localized laser energy deposition causes the fast cooling of the electrons and the lattice at later times. After about 50 ps from the laser pulse, the lattice temperature at the edge of the melted region drops below the equilibrium melting temperature, starting a slow process of epitaxial crystallization. It is interesting to observe that no evaporation or significant plastic deformation of the Ni film is observed in the simulations. Then, the area of the nanobump undergoes a transient melting and the hydrodynamic motion of the liquid region of the film prior to the resolidification establishes the shape of the nanobump.

Even if the simulations presented by Ivanov et al. [62] are strictly valid in the established conditions, which cannot be directly compared to real situations, however, they appear as a general guide for the study of the basic mechanisms involved in the nanobumps’ and nanojets’ formation. The simulations, in fact, for example, are strongly limited due to the very small size (10 nm) of the initial energy while the actual diffraction limited fs-pulsed laser spots are at minimum 40–50 times larger. However, scaling arguments can be used to generalize, at least qualitatively, the theoretical results by Ivanov et al. to real situations. So, while the quantitative results showed in Figure 50 could change for real situations, the general conclusions regarding the mechanisms for the metal film detachment from the substrate under the fs-pulsed laser can be regarded as reliable pathways for real cases as well.

**Figure 51:**
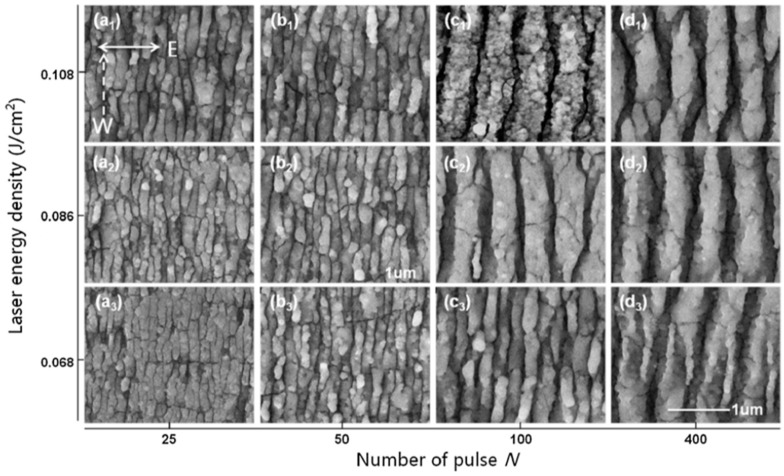
The representative SEM images of the surface of Fe films (about 500 nm-thick) on the Si substrate and processed by laser pulses of wavelength 800 nm, pulse duration of 50 fs and increasing the number of pulses from left to right (25, 50, 100, 400) and increasing the laser fluence from bottom to top (0.068, 0.086, 0.108, J/cm^2^). The number of pulses increases from (**a_1_**) to (**d_1_**), from (**a_2_**) to (**d_2_**), from (**a_3_**) to (**d_3_**), the laser fluence increases from from (**a_3_**) to (**a_1_**), from (**b_3_**) to (**b_1_**), from (**d_3_**) to (**d_1_**). Reproduced with permission from [80]. Copyright Elsevier, 2017.

To conclude this section, we mention that as recently observed [79,80,81,82,83], fs-pulsed laser irradiation of metal films is an effective method to produce periodic surface structures on metal film surfaces (for the sake of completeness, we mention that ns-pulsed laser irradiations, in some conditions, can result in periodic surface structures on metal surfaces [81]). Despite the different pulse durations for the fs-pulsed laser and ps-pulsed laser, generally, the same stages for the formation of the periodic surface structures are observed under similar laser beam parameters. However, some differences are observed depending on the ps and fs regimes. For example [79,80,81,82,83], laser-induced periodic surface structures obtained with a ps-pulsed laser are, typically, larger than those produced with a fs-pulsed laser due to the thermal effect reduction. As an example, Liu et al. [80] produced periodic surface structures on the surface of the Fe films (thickness in the 400–500 nm and deposited on the Si substrate) using laser pulses of wavelength 800 nm and with a pulse duration of 50 fs. Figure 51 reports a summary of the representative scanning electron microscopy images of the surface structures obtained by changing the laser fluence and the number of pulses. In particular, the authors [80] observed the formation of high-spatial frequency periodic surface structures with periods of 150–230 nm for a number of laser pulse lower than 100; on the other hand, they observed the abrupt formation of low-spatial frequency periodic surface structures with periods of 500–640 nm when the number of laser pulses was increased to a specific value which is dependent on the laser fluence. The authors ascribed the formation of laser-induced periodic surface structures, mainly, to surface plasmon polaritons excited by the laser on the Fe film.

## 6. Some Potential Applications for Laser-Nanostructured Metal Films

The production of metal nanostructures on surfaces is of paramount importance in nanotechnology research from the scientific viewpoint and technological applications in areas such as catalysis, photonics, plasmonic, solar cells, single electron and quantum devices, etc. [12,13,14,15,16,17,18,19,20,21,22,23,24,25]. In particular, metal nanoparticles (like Au and Ag) on functional substrates show interesting optical properties. These properties arise from the occurrence of localized surface plasmon resonance effects resulting in structure-dependent transmission/absorption spectra which can be exploited in several devices ranging from plasmonic solar cells to surface-enhanced Raman scattering-based sensors. In these applications, the shape- and size-control of the nanostructures is of paramount importance to control the characteristics of the localized surface plasmon resonance effects for desired applications. The ns-, ps-, and fs-pulsed laser nanostructuration effects of metal films deposited on functional substrates provide the possibility to produce, in a controlled way, functional-designed metal nanostructures. The control of the laser parameters, film thickness, substrate properties open the possibility for the wide-range control of nanostructure characteristics, providing a simple, versatile, cost-effective, high-throughput fabrication approach. In this section, we discuss, shortly, some applications of metal nanostructures produced on surfaces by the ns-, ps-, fs-pulsed laser processing of deposited metal films.

**Figure 52:**
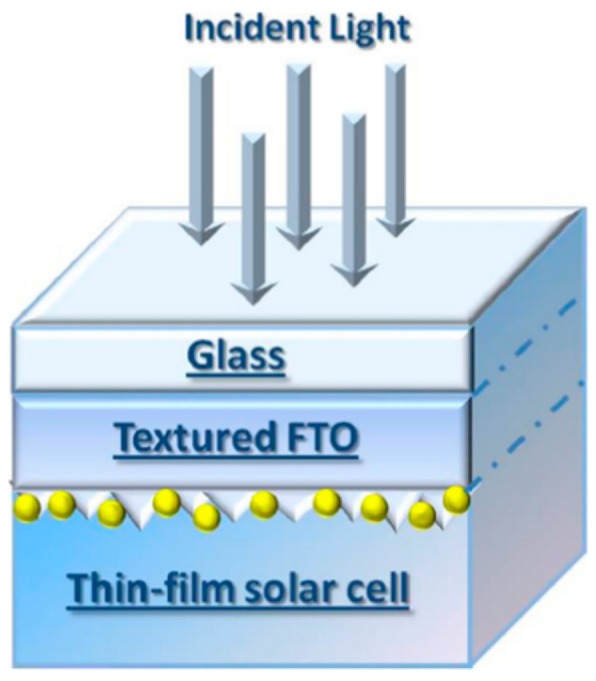
The schematic representation of a plasmonic solar cell prototype. In the glass/Fluorine-doped Tin Oxide (FTO)/Au nanoparticles (yellow dots) multilayer, the large fraction of radiation transmitted by the transparent layer interacts with the nanoparticles used as sub-wavelength scattering elements to couple and trap the sunlight into an absorbing semiconductor thin film by folding the light into the absorber layer. Reproduced with permission from [50]. Copyright Springer, 2014.

Gentile et al. [25,50] used nanosecond-pulsed laser irradiations to process nanoscale-thick Au films deposited on Fluorine-doped Tin Oxide (FTO) to fabricate plasmonic Au nanoparticles to be exploited for the design and production of plasmonic solar cells; see Figure 52.

**Figure 53:**
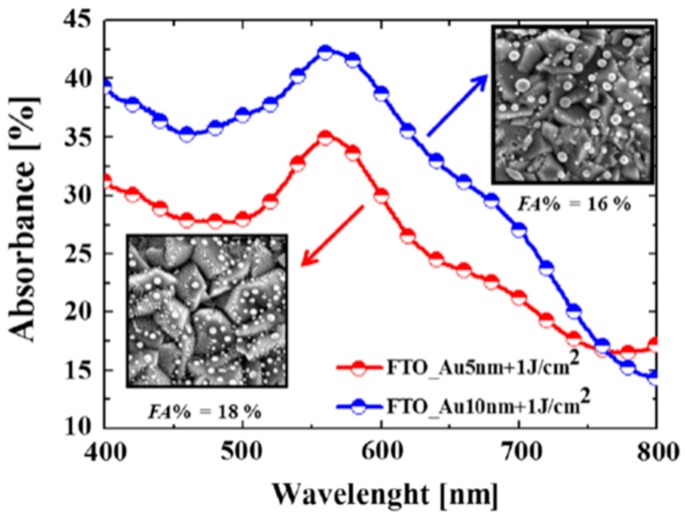
The comparison between the absorbance values of the substrates covered by 5 nm (red curve) or 10 nm of Au (blue curve) and processed by a 12 ns-pulsed laser (1 pulse) with a fluence of 1 J/cm^2^. The insets show the corresponding SEM images of the Au nanoparticles on the fluorine-doped tin oxide surface and the values of the nanoparticles covered area (FA%). Reproduced with permission from [50]. Copyright Springer, 2014.

In this work, the authors deposited 5 nm- or 10 nm-thick Au films on FTO and processed the films by one laser pulse with a duration of 12 ns with a fluence in 0.50–1.0 J/cm^2^. Figure 53 reports, as an example, the representative SEM images (insets) of the Au nanoparticles obtained on the FTO surface by irradiating the 5 nm-thick or the 10 nm-thick deposited Au film with 1 laser pulse of fluence = 1.0 J/cm^2^. In the first case, Au nanoparticles with a mean radius of about 18 nm, surface density of about 1.8 × 10^10^ cm^−2^ and fraction of covered area of about 18% are obtained. In the second case, Au nanoparticles with a mean radius of about 24 nm, surface density of about 9.3 × 10^9^ cm^−2^ and fraction of covered area of about 16% are obtained. The optical properties of these nanoparticles were studied by absorbance measurements, as reported in Figure 53. The absorbance spectra show the characteristic Au nanoparticles’ plasmonic absorbance peak at a wavelength of about 560 nm. The sample with larger Au nanoparticles presents a higher absorption in the analyzed spectral range. This is due to the larger fraction of the radiation diffused in the forward and reverse directions by Au nanoparticles with a radius of about 24 nm than the absorbed component. An inverse condition is, instead, realized for the nanoparticles with a mean radius of 18 nm [50]. These results for the 24 nm-sized Au nanoparticles were exploited for the production of a plasmonic solar cell by growing, on the FTO/Au nanoparticles substrate, a Si-based thin film solar cell [25]. The plasmonic properties of metal nanoparticles such as Au and Ag nanoparticles can be exploited to fabricate sensors based on the Surface-Enhanced Raman Scattering (SERS) effect [23].

**Figure 54:**
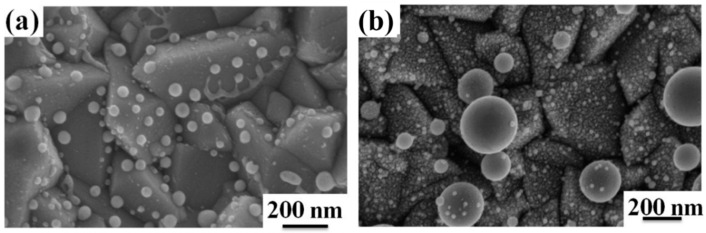
The SEM images of the FTO surface covered by the Pd film after the 0.50 J/cm^2^ laser pulse ((**a**) 3 nm-thick, (**b**) 27.9 nm-thick). Reproduced with permission from [52]. Copyright MDPI, 2019.

**Figure 55:**
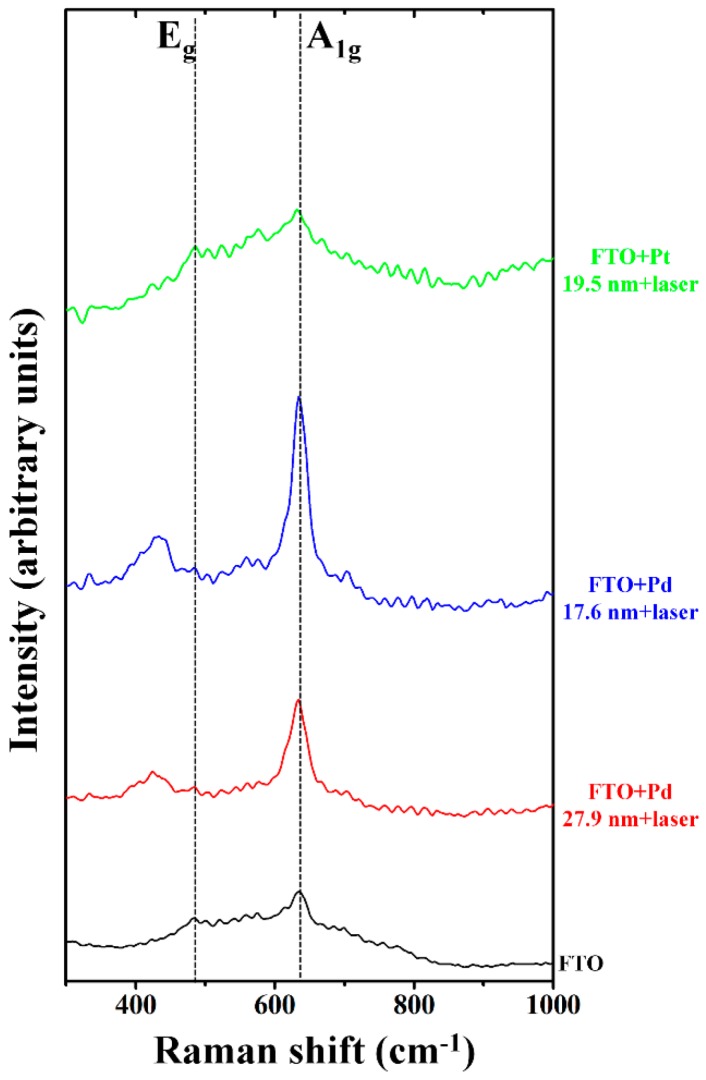
The Raman spectra corresponding to the bare FTO substrate (black), FTO covered by Pd NPs obtained by the laser irradiation of the 27.9 nm-thick Pd film (red) and of the 17.6 nm-thick Pd film (blue), FTO covered by Pt NPs obtained by the laser irradiation of the 19.5 nm-thick Pt film. The SERS effect can be particularly recognized in the blue spectrum. Reproduced with permission from [52]. Copyright MDPI, 2019.

The SERS effect for metal nanoparticles produced on surfaces by the nanosecond laser irradiations of deposited films was, for example, studied by Henley et al. [28] (Ag nanoparticles) and Torrisi et al. [52] (Pd and Pt nanoparticles). Regarding the work of Torrisi et al. [52], Figure 54 reports the representative SEM images of Pd nanoparticles produced on the FTO surface by 1 laser pulse of duration 12 ns, fluence of 0.50 J/cm^2^, and film thickness of 3 nm (a) and 27.9 nm (b). According to the authors’ data, Figure 55 shows in black the Raman spectrum of the bare FTO/glass substrate. There are two fundamental Raman scattering peaks which are the characteristic of the rutile SnO_2_ single crystal. For pure SnO_2_, the band at 625 cm^−1^ corresponds to the A_1g_ vibration mode of SnO_2_, and the band around 478 cm^−1^ to the E_g_ vibration modes of SnO_2_. The figure also shows the Raman spectra of the FTO/glass substrate covered by Pd or Pt nanoparticles: in red, the spectrum of the substrate covered by Pd nanoparticles obtained by the laser irradiations of the 27.9 nm-thick Pd film; in blue, the spectrum of the substrate covered by Pd nanoparticles obtained by the laser irradiations of the 17.6 nm-thick Pd film; in green, the spectrum of the substrate covered by Pt nanoparticles obtained by the laser irradiations of the 19.5 nm-thick Pt film. The general effect is that, compared with bare FTO, the intensity of Pd nanoparticles/FTO samples become stronger.

On the contrary, the presence of the Pt nanoparticles on the FTO surface does not influence the FTO peak intensity, indicating no Pt nanoparticles-FTO interaction. In particular, the Pd nanoparticles’ SERS enhancement may be due to the Pd nanoparticles’ surface plasmon resonance.

**Figure 56:**
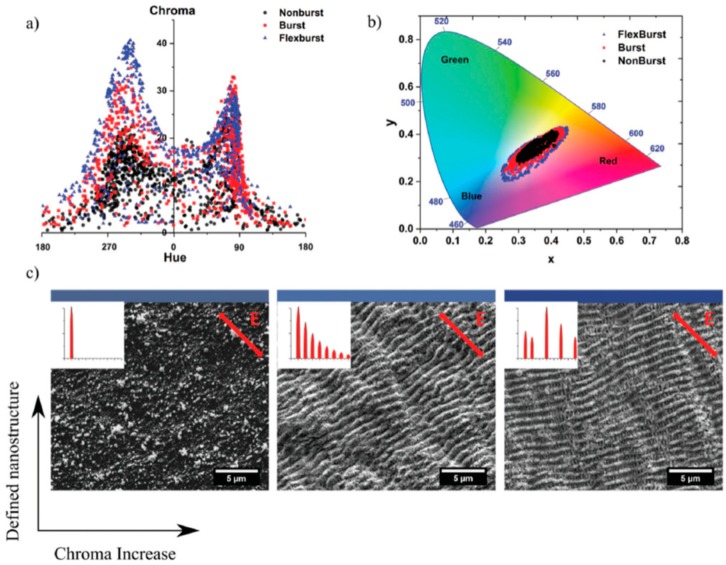
The colors and topography of the obtained Ag surfaces: (**a**) The plot of Chroma versus Hue comparing colors obtained using the nonburst (i.e., 1 burst, black circles), burst (i.e., 2 or more bursts with uncontrolled energy distribution, red squares), and flexburst (i.e., 2 or more bursts with controlled energy distributions, blue triangles) coloring methods. (**b**) Commission Internationale de l’Eclairage (CIE) xy Chromaticity diagram comparing the nonburst (i.e., 1 burst, black circles), burst (i.e., 2 or more bursts with uncontrolled energy distribution, red squares), and flexburst (i.e., 2 or more bursts with controlled energy distributions, blue triangles) coloring methods of (a). (**c**) SEM images of blue surfaces produced using the nonburst (left), burst (middle), and flexburst (right) coloring methods. The Hue is about the same for all squares (H ≈ 295°) whereas the Chroma values are 22.3, 31.2, and 39.44. Significant nanostructures are observed on the surfaces for the cases of burst and flexburst. The relative energy distribution of the burst pulses is shown as the insets along with the orientation of the electric field (E) applied during laser irradiation. Reproduced with permission from [139]. Copyright Wiley, 2018.

Regarding the nanostructuration of metal films by picosecond-pulsed laser irradiations, for example, Guay et al. [139] reported on the fabrication and tuning of three-dimensional topographical features on Ag for the production of plasmonic colors. In particular, the irradiation of Ag surface was carried out in a raster-scanning mode with a laser of wavelength 1064 nm, pulse duration 10 ps, and burst repetition frequency 25 kHz. By using these laser burst, an increase of the Chroma (i.e., color saturation) of the colors up to 100% compared to the nonburst coloring method was observed; see Figure 56a,b.

By adjusting the energy distribution of the laser pulses in a burst, while maintaining the total burst energy constant, significantly different color palettes and topographical structures are produced; see Figure 56c. In particular, from a topographical point of view, the formation of laser-induced periodic-like surface structures, low spatial frequency laser-induced periodic-like surface structures, high spatial frequency laser-induced periodic-like surface structures, and large laser-induced periodic-like surface structures can be recognized (Figure 56c). Figure 56a,b show that compared to nonburst, the burst and flexburst coloring methods enhance the Chroma across the full Hue range by up to about 70% and about 100%, respectively. On a CIE (Commission Internationale de l’Eclairage) diagram, the Chroma increases from the center going outwards. The highest increase in Chroma values is observed to be within the blue and magenta regions. Burst (2 or more bursts with uncontrolled energy distribution) and flexburst (2 or more bursts with controlled energy distribution) produce more bright colors. The surface of the nonburst blue (Figure 56c, left) is flat without characteristic features. Instead, the burst Ag surface presents the combination of well-defined low spatial frequency laser-induced periodic-like surface structures and a high spatial frequency laser-induced periodic-like surface (Figure 56c, center). The flexburst Ag surface presents a well-defined high spatial frequency laser-induced periodic-like surface with periods of about 200 and about 1000 nm, respectively (Figure 56c, right). The Chroma increase is due to stronger electric fields on the structured surfaces than on the non-structured surfaces under optical radiation. In fact, the periodic surface structures enhance the radiation fields, consequently enhancing the resonances and the absorption and resulting in more vivid colors in the far-field. Regarding the nanostructuration of metal films by femtosecond-pulsed laser irradiations, for example, Kuchmizhak et al. [140] reported on the fabrication micro-holes and nanojets in Ag films showing a strong polarization-dependent enhancement of surface-enhanced photoluminescence and surface-enhanced Raman spectroscopy responses from a nanometer-thick covering Rhodamine 6G layer with average enhancement factors of 40 and 2 × 10^6^, respectively, with perspectives in sensing applications. The laser irradiations were performed on 500 nm-thick Ag films deposited on silica glass and using a 200 fs-pulsed laser with a wavelength of 400 nm and a fluence ranging from 1.2 to 8.4 J/cm^2^.

**Figure 57:**
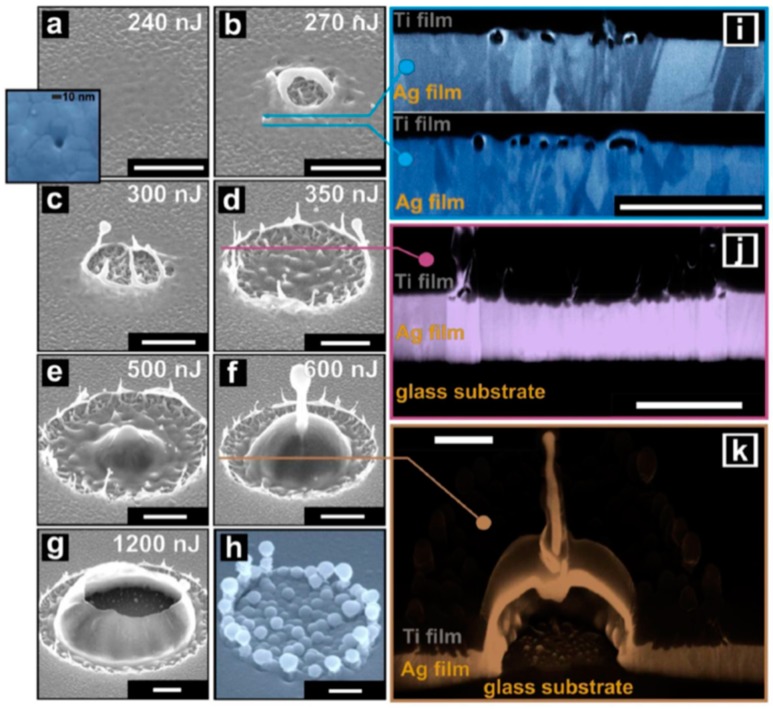
The single-pulse nano-structuration of 500 nm-thick Ag films: (**a**–**g**) tilted-view SEM images showing the topography of the Ag film increasing the laser energy from 240 to 1200 nJ (i.e., from 1.2 to 8.4 J/cm^2^ laser fluence); (**h**) tilted-view false-color SEM image showing the typical single-pulse nanotopography covered by a 500 nm thick Ti protective layer; (**i**−**k**) tilted SEM images of focused-ion-beam cross-sectional cuts of the three main types of the ablative structures presented in (b,d,f) respectively. The scale bar in all images is 1 μm. Reproduced with permission from [140]. Copyright American Chemical Society, 2016.

Figure 57 reports a series of SEM images showing the fluence-dependent morphology change for the 500 nm-thick Ag film irradiated by a single femtosecond laser pulse. Starting from the re-melting, smoothing, and recrystallization within the irradiated region, accompanied by the appearance of 10 nm-diameter pits (Figure 57a and its inset) at a fluence of 1.4 J/cm^2^, the Ag surface morphology evolves towards the emergence of a crater at higher fluences (Figure 57b–d). At such high fluences, the irradiated film shows the formation of the porous sub-surface layer, containing nanosized voids and, at further higher fluences, the subsequent ejection of the upper layer of the molten film, accompanied by the appearance of a number of nanosized tips inside the crater and larger micron-tall tips along the outer rim of the crater. The focused-ion-beam milling prepared cross sections illustrate these two main processes—Figure 57g–i—at the near-threshold fluences, whereas prior to the milling, the ablated areas were protected by the 500 nm-thick Ti layer to avoid the edge curtain effect (Figure 57h). Furthermore, high crystallographic channeling contrast can be recognized in the SEM images (Figure 57i), also indicating the pronounced diminishing of the crystalline grains in the Ag film under and near the ablated area due to fast recrystallization.

**Figure 58:**
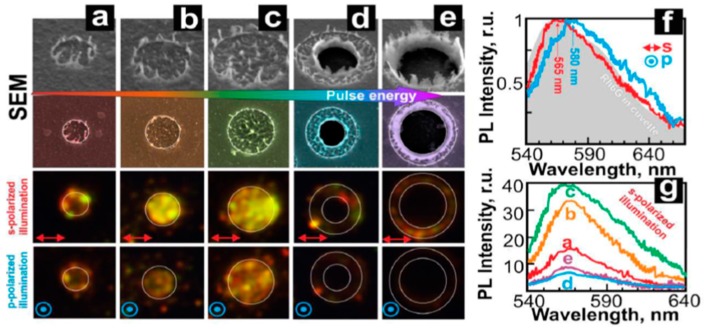
The enhancement of spontaneous photoluminescence from R6G molecules on the single nanotextures. (**a**–**e**) Reference tilted-view and top-view SEM images (two upper-most rows, respectively) of the nano-textured craters and double-scale structures produced at the gradually increasing peak fluence, as well as their surface-enhanced photoluminescence images (two bottom rows) obtained under the lateral oblique excitation (the angle of 75° to the sample normal) of the 10 nm-thick layer of R6G molecules with the s- and p-polarized 532 nm continuous-wave laser source with an average excitation fluence of 6 mW/cm^2^. The dashed white circles in the surface-enhanced photoluminescence images denote the outer dimensions of the craters and through holes, while the blue and red arrows show the polarization direction of the excitation laser source. (**f**) Normalized surface-enhanced photoluminescence spectra of the R6G layer measured from the 4-μm wide single crater under the s- and p-polarized lateral irradiation. The gray area shows the photoluminescence spectrum measured from the R6G ethanol solution in the cuvette. (**g**) Normalized surface-enhanced photoluminescence spectra measured from the R6G layer, covering the single craters presented in (**a**–**e**) under their p-polarized excitation. Each SEPL spectrum was averaged over 50 of the same spectra measured from similar structures and then normalized on the spectrum measured from the R6G layer, covering a non-irradiated (smooth) Ag-film region of the same size Reproduced with permission from [140]. Copyright American Chemical Society, 2016.

The hole formed at the above-threshold fluences (Figure 57j) shows a rough profile and the ejection of a 100 nm-thick molten layer of the film, while some nanovoids can also be recognized along the larger hole edges. In order to illustrate the plasmon-mediated optical response of the produced Ag nanostructures, the authors deposited a 10 nm-thick layer of R6G dye molecules on the Ag nanotextured surfaces. Figure 58 reports a series of polarization-resolved surface-enhanced photoluminescence images obtained for the different Ag nanostructures and R6G marker: they indicate that for all these Ag nanostructures, the larger enhancement in the photoluminescence response is observed under the s-polarized illumination, i.e., when the polarization direction is perpendicular to the major axis of the nanotips. This feature is explained by the more favorable conditions for the resonant excitation of plasmonic oscillations under the s-polarized light illumination, providing more intense plasmon-mediated near-field hot spots. The stronger signal is obtained for the holes with the maximal averaged 40-fold plasmon-mediated enhancement factor for the spontaneous R6G emission as compared with the photoluminescence signal obtained from the flat (unprocessed) Ag film surface (Figure 58g). As a result, such shaped Ag nanostructures can be considered microscale sensitive elements of biosensors with efficient sensing characteristics. Overall, in this discussed example, even if the increased photoluminescence and Raman signal of Rhodamine 6G on top of the Ag laser nanostructured sample appear moderate when compared to that obtained using colloidal prepared noble metal quantum dots, these results are, surely, promising considering further optimization of the structural and morphological properties of the laser-generated metal structures.

**Figure 59:**
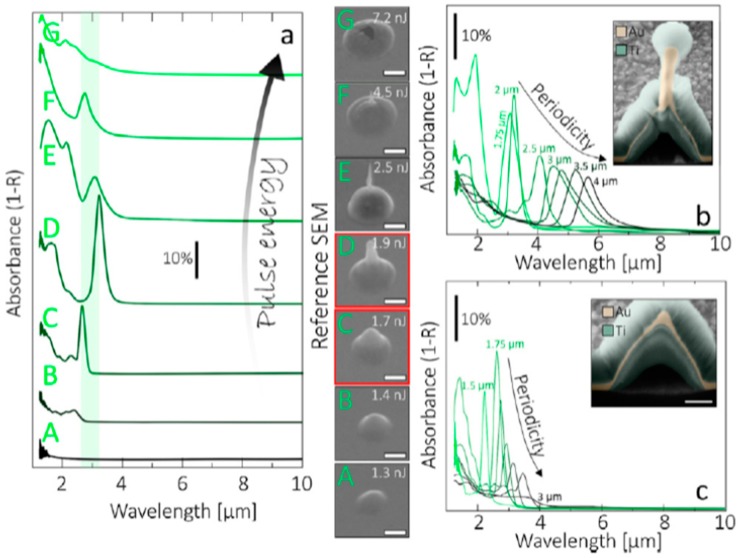
The optical infrared properties of laser-generated arrays of the surface features (nanobumps and microjets) on Au films (50 nm-thick) deposited on silica (laser wavelength of 515 nm, pulse duration of 230 fs). In particular, (**a**) presents normalized absorbance spectra acquired for the arrays formed by various types of surface textures as well as corresponding side-view SEM image showing the geometry evolution of one of the structures of the array. The square-shape arrays are printed to have an identical number of structures (100 × 100) within and a fixed periodicity of 2 μm. The type of structure is varied by tuning the laser energy (as reported in the SEM images). The scale bar of the SEM images corresponds to 400 nm. (**b**,**c**) report the normalized absorbance (1−R) spectra for two fixed types of the surface structures, cone-shape nanobumps and nanojets, in arrays fabricated at various periods. The array period varies from 1.5 to 4 μm and is indicated near each spectrum. The insets demonstrate the cross-section Focused-Ion-Beam cuts showing the real geometry of the structures under study. The scale bar is 200 nm. Noteworthy, the 200-nm thick Ti protective layer was coated above the laser-produced Au textures prior to the FIB milling. In the process of FIB cutting, the redeposition of the Ti material occurs onto the bottom part of the hollow Au structure. Reproduced with permission from [76]. Copyright Elsevier, 2019.

**Figure 60:**
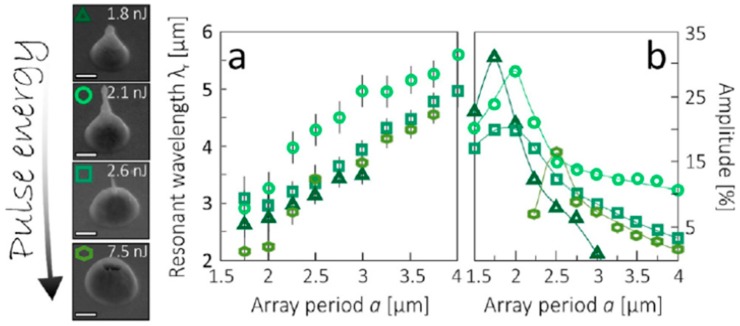
The resonant wavelength λ_r_ (**a**) and resonance modulation amplitude (**b**) versus the array period measured for several types of the laser-generated structures shown on the SEM images (laser wavelength of 515 nm, pulse duration of 230 fs). The scale bar of the SEM images corresponds to 400 nm. Reproduced with permission from [76]. Copyright Elsevier, 2019.

Between the multitude of possible applications for the ns-, ps-, fs-pulsed laser-structured metal films, however, the most technologically promising are, surely, related to plasmonic surfaces and metasurface optics applications [76,77,78]. In this regard, for example, recently, Pavlov et al. [76] reported on the application of femtosecond laser processing for the production, on the surface, of periodic nanoantenna structures with various geometries and periods, printed on pure or alloyed noble metal films over a silica substrate (see Figure 59 and Figure 60). By varying the applied pulse energy, they realized a wide range of possible morphologies, from smooth nanobumps to protruding nanojets with up to 1 μm height, and finally through to microholes. Using several pulse energy levels, they have fabricated periodic nanojet arrays with periods from 1.75 to 4 μm and measured their infra-red (IR) reflection spectra. The resonance frequency and magnitude of the resulting absorbance were found to essentially depend on both the periodicity of the arrays and nanojet geometry. The authors explain these observations by considering the nanojet-assisted plasmon excitation running along the nanojets and along the surface and found a convincing agreement to the experiments. They have also applied the fs-laser nanofabrication approach to a variety of noble metal alloys involving Au, Ag and Pd in various combinations and compositions and showed that nanojets can be printed for such alloys, preserving their chemical composition and its homogeneous volumetric distribution. Regarding their results, as an example, Figure 59 reports the optical infrared properties of laser-generated arrays of the surface features (nanobumps and microjets) on Au films (50 nm-thick) deposited on silica (laser wavelength of 515 nm, pulse duration of 230 fs). In particular, the images in Figure 59a present the normalized absorbance spectra acquired for arrays formed by various types of surface textures as well as the corresponding side-view SEM image showing the geometry evolution of one of the structures of the array. The square-shape arrays are printed in order to have an identical number of structures (100 × 100) within and a fixed periodicity of 2 μm. The type of structure is varied by tuning the laser energy (as reported in the SEM images). The images in Figure 59b,c report the normalized absorbance (1−R) spectra for two fixed types of the surface structures, cone-shape nanobumps and nanojets, in arrays fabricated at various periods. The array period varies from 1.5 to 4 μm. These results clearly show the possibility to largely tune the arrays’ plasmonic properties by tuning the arrays’ geometrical characteristics. This is summarized by Figure 60 where the resonant wavelength λ_r_ (a) and resonance modulation amplitude (b) are reported versus the array period, measured for several types of laser-generated structures shown on the SEM images.

In the same research field, Kudryashov et al. [78] reported on the use of tightly focused, highly spatially multiplexed femtosecond laser pulses, coming at sub-MHz repetition rates, to produce plasmonic films by creating arrays of holes in Ag, Al, Cu, AuPd films.

**Figure 61:**
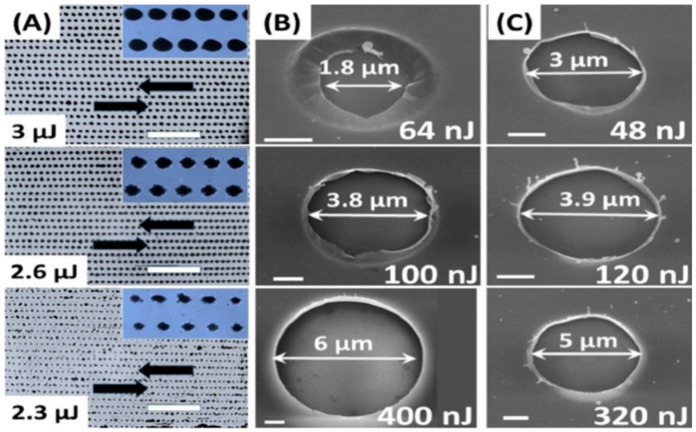
(**A**) The reported optical images of directly laser-patterned 3 × 3 mm^2^ arrays on Ag film (50 nm-thick, on silica) at frequency f = 500 kHz, laser scanning velocity *v* = 7 m/s, filling factor = 80 lines/mm (corresponding to interline separation of 12 μm), at variable pulse energies of 3 (**top**), 2.6 (**middle**) and 2.3 (**bottom**) μJ, using the standard F-Theta objective with the 100-mm focal length (laser wavelength of 1030 nm, pulse duration of 300 fs). (**B**,**C**) The reported of top-view SEM images of separate through microholes in the Ag film at different focusing NA = 0.25 and 0.65, respectively, with the corresponding pulse energies indicated in the bottom corners of the images. Reproduced with permission from [78]. Copyright Elsevier, 2019.

**Figure 62:**
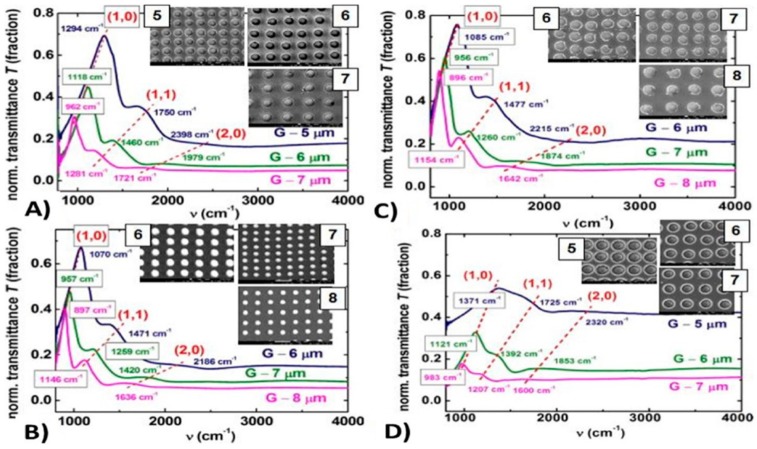
The normalized transmittance spectra of micro-hole gratings (gratings G, fixing the holes diameter to D≈4 μm) on the 50-nm thick Ag (**A**), Al (**B**), Cu (**C**) and Au-Pd alloy (**D**) films on the CaF_2_ substrates with variable periods (shown by the same colors as the corresponding spectra), the colored numbers showing the spectral positions of their (1,0), (1,1) and (2,0)-peaks and the red dashed lines showing their evolution versus the hole array period P. Insets: top-view SEM images of the gratings with periods shown in microns in the frames (scale bars can vary). Reproduced with permission from [78]. Copyright Elsevier, 2019.

In particular, they were able to produce large (~10^5^–10^6^ holes per array) arrays of micro-holes of variable diameters and periods in thin Ag, Cu, Al, AuPd films of different thicknesses (Figure 61). These arrays were, then, characterized in the broad IR range (1.5–25 μm) in terms of plasmonic effects showing an extraordinary optical transmission (Figure 62) and presenting an increasing wavenumber, a smooth transition from the common Bethe-Bouwkamp transmission to its plasmon enhanced analogue, ending up with a common geometrical (wave-guide-like) transmission.

Figure 62 clearly shows the possibility to tune the optical properties (transmittance spectra in this case) of the hole array by changing, for example, the array period.

## 7. Conclusions, Perspectives, and Challenges

**Figure 63:**
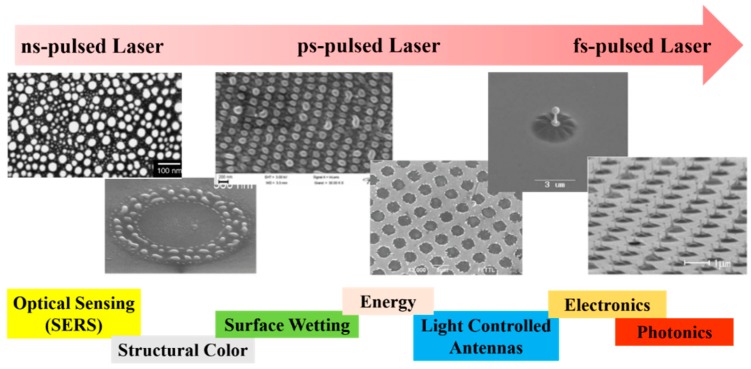
The picture presenting a summary of the metal nano- and micro-structures which can be produced on surfaces by pulsed-laser processing of thin metal films deposited on substrates and the corresponding potential applications.

In this paper we reviewed the basic concepts related to the exploitation of laser-matter interaction as an effective nanofabrication tool. In particular, we focused our attention on the use of nanosecond-, picosecond-, femtosecond-pulsed lasers as a nanofabrication tool for metal nanostructures by processing deposited metal films. The main highlight of the review was the possibility to control the nanostructures characteristics (as size, morphology, shape, structure) by controlling the laser parameters (pulse duration, wavelength, energy), film characteristics (type of metal, thickness), properties of the supporting substrate (thermal conductivity). Due to the many unique properties of the fast and ultrafast laser-fabricated metal nanostructures, we believe that the detailed understanding of the basic physical phenomena governing the nanostructures’ formation can allow the desired control over the properties and applications. The review, therefore, emphasizes the basic microscopic mechanisms and processes and the general physical concepts suitable for the interpretation of the materials’ properties and the structure-property correlations. Besides the basic processes and the general concepts, the review aims at a comprehensive schematization of the main classes of nanostructures which can be produced by also emphasizing the technological applications actually in development worldwide. Figure 63 summarizes some classes of the surface structures which can be produced on metal films processed by ns-, ps-, fs-pulsed laser irradiation and some corresponding applications, spanning from optical sensing and structural color to surface wetting, energy, electronics and photonics.

**Figure 64:**
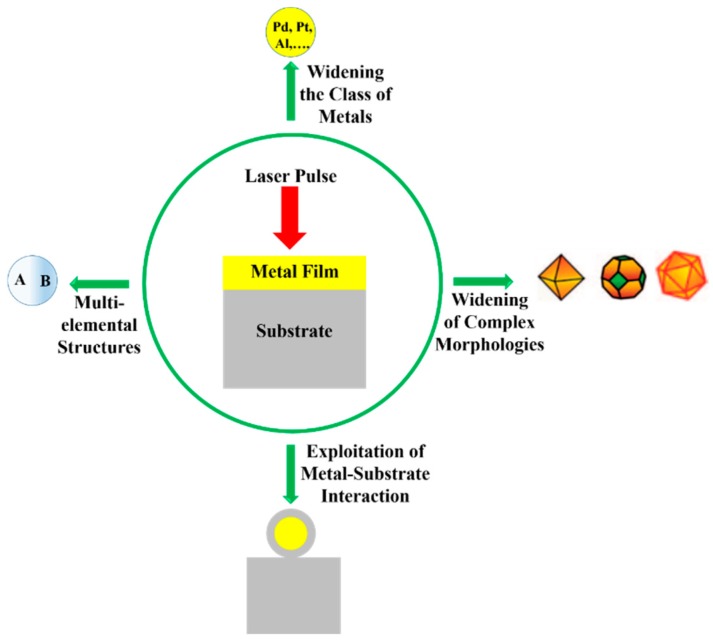
The picture presenting some potential outlooks for pulsed-laser irradiations of deposited metal films in view of nano-fabrication: the production of multi-elemental structures by processing multi-elemental films or by exploiting chemical reactivity of the metal film to the substrate, widening the morphology control on the produced structures, and widening the class of processable metals to less-studied metal (such as Pd, Pt, Al).

The future of the use of nanosecond-, picosecond-, femtosecond-pulsed lasers to structure metal films on the nanoscale strictly depends on the control of the formation mechanisms of the metal nanostructures and on the improvements in combined techniques to produce one-, two-, three-dimensional nano-architectures with a complex morphology and chemical composition. New opportunities will be provided by an unprecedented control over the spatial distribution, orientation, and shape of the metal nanostructures [141,142,143]. Figure 64 summarizes some outlooks for the pulsed-laser use in nanostructuration. In this sense, the use of interfering lasers [31,61] of the pulsed-laser processing of metal films in liquids exploiting the Rayleigh-Taylor phenomenon [144,145] and the pulsed-laser processing of multielement (as alloyed) films or multilayered metallic films [146,147,148,149] will be the key approaches. In particular, in this last case, alloyed and multi-elemental nanostructures can be produced with innovative properties arising from the synergistic combination of the properties of the metallic components. In general, in order to facilitate future applications of laser-fabricated metal nanostructures, there is a need for methods to obtain chemical and shape complex nanoscale architectures which could find forefront applications in catalysis and plasmon-based sensing.

Another interesting point regards the fabrication of new metal nanostructures. To date, the main literature has been focusing on Ag and Au and less studies are devoted to Ni, Fe, Mo, Co, Cu, Pd and Pt. Probably, the widening of the class of deposited metal films which can be nanostructured by pulsed lasers will open new technological perspectives and challenges. In this sense, for example, the nanostructuration effects of pulsed lasers on Al films to produce plasmonic nanostructures could be of high impact so as to reduce the cost with respect to the use of Au and Ag. Similarly, laser-fabricated Pd and Pt nanostructures could be of high interest in areas such as surface catalysis and hydrogen storage.

An additional aspect which needs further investigation and widened regards the metal interaction caused by the laser irradiation with the supporting substrate: further supporting substrates could be used with some degree of reactivity with the specific supported metal so as to give origin to the nanostructures whose chemical composition could be influenced by the substrate material (as in the case of Au, Ag, Co, Pd, Pt on the Si substrate).

## Figures and Tables

**Table 1:** This table summarizes some of the recent literature in which nano-, pico-, femto-second pulsed lasers are used to structure, on the micro- and nano-scale, thin metal films on substrates. The table groups of the literature work on the basis of the laser (nano-, pico-, femto-second pulsed), processed metal films, type of substrate supporting the metal film, and typology of the obtained structures after laser processing.

Laser (Time Pulse, Wavelength)	Metal Film	Substrate	Formed Nanostructures	Reference
25 ns, 248 nm	Au, Ag, Ni, Ti, Zn	SiO_2_	Nanoparticles	[26,27,28]
				
25 ns, 248 nm	Ag	ITO	Nanoparticles	[34]
2				
9 ns, 266 nm	Co	SiO_2_	Nanoparticles	[30,32,33,35,37,39]
				
9 ns, 266 nm	Co	Si	Nanoparticles	[31]
				
9 ns, 266 nm	Fe	SiO_2_	Nanoparticles	[37]
				
5 ns, 266 nm	Au	Al_2_O_3_	Nanoparticles	[38]
				
5 ns, 266 nm	Au	GaN	Nanoparticles	[38]
				
5 ns, 266 nm	Au	SiO_2_	Nanoparticles	[38]
				
9 ns, 266 nm	Ag	SiO_2_	Nanoparticles	[39,40]
				
25 ns, 248 nm	Cu	SiO_2_	Nanoparticles	[41,42,48]
				
18 ns, 248 nm	Ni	Si	Nanoparticles	[43]
				
12 ns, 532 nm	Au	SiO_2_	Nanoparticles	[45]
				
12 ns, 532 nm	Au, Ag	ITO	Nanoparticles	[46]
				
12 ns, 532 nm	Au	Si	Nanoparticles	[47]
				
5 ns, 532 nm	Au	SiO_2_	Nanoparticles	[49]
				
12 ns, 532 nm	Au	FTO	Nanoparticles	[50]
				
7 ns, 1064 nm	Au	SiO_2_	Nanoparticles	[51]
				
12 ns, 532 nm	Pd, Pt	FTO	Nanoparticles	[52]
				
10 ps, 1030 nm	Au, Al, Pt, Ni, Mo	Glass	Microbumps, Microholes	[54]
				
60 ps, 1064 nm	Cr, Cu, Al, Ag, Au	Glass	Nanoholes	[55]
				
42 ps, 266 nm	Cu	Si, glass	Nanospikes	[56]
				
30 fs, 800 nm	Au	Glass, quartz	Microbumps, Nanobumps, Nanojets	[58,59,63,66]
				
120-355-1220 fs, 780 nm	Au	Quartz	Nanobumps	[61]
